# Adaptive wireless millirobotic locomotion into distal vasculature

**DOI:** 10.1038/s41467-022-32059-9

**Published:** 2022-08-01

**Authors:** Tianlu Wang, Halim Ugurlu, Yingbo Yan, Mingtong Li, Meng Li, Anna-Maria Wild, Erdost Yildiz, Martina Schneider, Devin Sheehan, Wenqi Hu, Metin Sitti

**Affiliations:** 1 Physical Intelligence Department, Max Planck Institute for Intelligent Systems, 70569 Stuttgart, Germany; 2 Department of Information Technology and Electrical Engineering, ETH Zurich, 8092 Zurich, Switzerland; 3 Clinic for Neuroradiology, Klinikum Stuttgart, 70174 Stuttgart, Germany; 4 Department of Biophysics, Aydın Adnan Menderes University, Graduate School of Health Sciences, 09010 Aydın, Turkey; 5 School of Medicine and College of Engineering, Koç University, 34450 Istanbul, Turkey

**Keywords:** Mechanical engineering, Biomedical engineering

## Abstract

Microcatheters have enabled diverse minimally invasive endovascular operations and notable health benefits compared with open surgeries. However, with tortuous routes far from the arterial puncture site, the distal vascular regions remain challenging for safe catheter access. Therefore, we propose a wireless stent-shaped magnetic soft robot to be deployed, actively navigated, used for medical functions, and retrieved in the example M4 segment of the middle cerebral artery. We investigate shape-adaptively controlled locomotion in phantoms emulating the physiological conditions here, where the lumen diameter shrinks from 1.5 mm to 1 mm, the radius of curvature of the tortuous lumen gets as small as 3 mm, the lumen bifurcation angle goes up to 120^°^, and the pulsatile flow speed reaches up to 26 cm/s. The robot can also withstand the flow when the magnetic actuation is turned off. These locomotion capabilities are confirmed in porcine arteries ex vivo. Furthermore, variants of the robot could release the tissue plasminogen activator on-demand locally for thrombolysis and function as flow diverters, initiating promising therapies towards acute ischemic stroke, aneurysm, arteriovenous malformation, dural arteriovenous fistulas, and brain tumors. These functions should facilitate the robot’s usage in new distal endovascular operations.

## Abstract (web summary)

Accessibility into the distal vascular systems to treat various diseases remains challenging using medical catheters. Here, Wang et al. demonstrate that a stent-shaped wireless magnetic soft robot enables adaptive locomotion and medical functions into these distal vascular regions.

## Introduction

Accessing the vascular system by microcatheters has offered new opportunities in minimally invasive diagnostic and targeted therapeutic procedures, which have been extensively used clinically in recent decades^1^. However, catheters have limited safe access into the distal vascular routes due to various factors^2–5^. First, the longer access route from the arterial puncture sites (radial artery or femoral artery), the vessel tortuosity, and the smaller vessels with thinner vessel walls increase the challenges of safely navigating and pushing the catheter into these regions without causing injuries^3,4^, by either manual^1,5^ or robotic insertion^6^. Second, some vascular lesions, such as thrombotic diseases and arteriosclerosis, decrease or even block the blood flow in arteries, making it impractical to utilize the flow to advance the catheter to the target position^4^.

As a typical example, the distally cortical arteries are limited to safely and effectively access by catheters to treat various lesions and diseases around this region, such as acute ischemic stroke (AIS)^3,7,8^, aneurysm^9,10^, cerebral arteriovenous malformation (CAVM)^11,12^, dural arteriovenous fistulas (dAVFs)^13,14^, and brain tumors^15,16^. Particularly, most malignant primary brain tumors occur in the cerebral cortex, with the highest percentage of 26% developing in the frontal lobe^15^, and dAVFs and 86% of the CAVM occur in the cortical region^12^. Although occurrence rates of cortical aneurysms and AIS were reported to be 1% and lower than 15%, respectively, it has also been raised that actual rates were underestimated due to the sensitivity of medical imaging^3,10^. Moreover, the hemispherical distal occlusions commonly lead to partial aphasia, fractionated hemiparesis or hemianesthesia, as well as partial or complete hemivisual field defects^3^. Cortical aneurysms are only manifested clinically after the rupture of their aneurysmal sac^10^, which would lead to various fatal complications and need to be treated early and properly^9^. Due to the challenge in safe reachability to the proximity of these targeted lesions by catheterization, the therapeutic agents, such as stents, coils, and medications, cannot be delivered accurately and efficiently (for details of the limitation on current endovascular therapies of the above diseases, please refer to Supplementary Table 1). Thus, the efficacy of therapies can be decreased^3,9,11^. Similar limitations also apply to other distal vascular routes^17^. Therefore, there are unmet needs for more effective medical tools for minimally invasive therapies in these distal arterial regions.

Burgeoning efforts have been made to develop wireless medical devices at the milli/microscale, which could enter these hard-to-reach sites because of their tetherless nature and smaller dimensions^18–27^. In tortuous distal vascular regions, these devices need to achieve shape adaption for changing lumen diameters, withstand the flow even if the external actuation is disrupted or turned off (i.e., self-anchoring capability) for safe local operations, traverse among curved routes and bifurcations, and be movable against the blood flow for retrieval or mistaken navigation. However, all the previous works have shown limitations in concurrently fulfilling all of these requirements^18–27^. Notably, most of the previous designs lack the self-anchoring capability with the surrounding lumen and could be drifted away by the pulsatile flow. The distribution of these drifted robots is unpredictable, and they could accumulate in non-target vessels and organs, which might lead to prolonged health risks^28^.

**Fig. 1:**
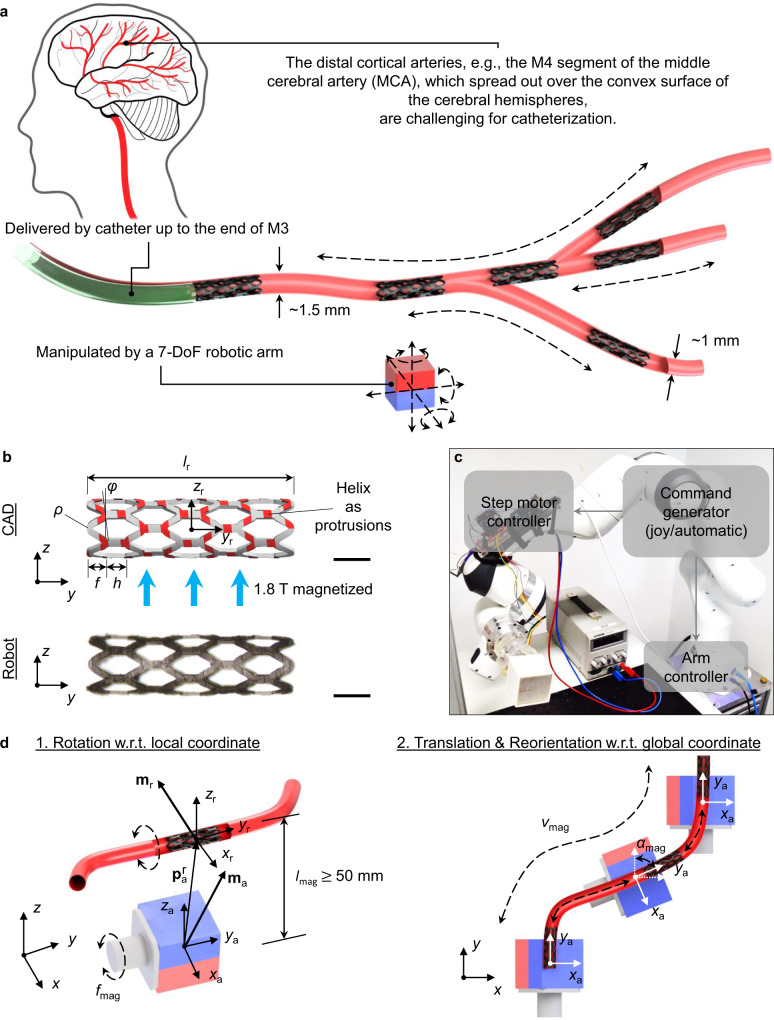
Design of the robotic system for distal-endovascular applications. **a** Overall concept of the application scenarios in the distal vasculature. The M4 segment of the middle cerebral artery (MCA) case is shown here, where catheterization is challenging. Major locomotion capability of the proposed wireless soft robot: forward and backward shape adaptation in lumens with varying diameters; flow withstanding when no magnetic field is applied; traversing among curved routes and branches. The robot can function as a mobile carrier for other functional tools to treat acute ischemic stroke, aneurysm, and arteriovenous malformation. **b** Robot CAD design drawing and the photo of the robot prototype. A stent-shaped structure was chosen for its high radial deformability and low fluidic drag property. There are three critical design parameters: the strut spacing *h*, the radius of curvature at the crown junction *ρ*, and the axial amplitude of each segment *f*. The NdFeB ferromagnetic microparticles inside the robot body were uniformly magnetized using 1.8 T homogeneous magnetic field. Right-handed helical structures with a helix angle *φ* were coated to assist the locomotion utilizing anisotropic frictional forces when the robot was rotated around its body-attached *y*_r_-axis. **c** Magnetic actuation system composed of a 7-DoF robotic arm, a step motor, and a 50 mm cubic NdFeB permanent magnet. The communication framework was realized by Robot Operating System (ROS). **d** Robot actuation using both magnetic torques and forces. The magnet with moment **m**_a_ is rotated around the *y*_a_-axis of the magnet-attached local coordinate *x*_r_-*y*_r_-*z*_r_, such that the robot at the ${{{{{{\bf{p}}}}}}}_{{{{{{\rm{a}}}}}}}^{{{{{{\rm{r}}}}}}}$ away from the magnet with moment **m**_r_ is rotated around the *y*_r_-axis of the robot-attached local coordinate *x*_r_-*y*_r_-*z*_r_ by magnetic torques (direction is reversed). The magnet is also translated with the speed of *v*_mag_ and reorientated with the angle of *α*_mag_ with respect to the global coordinate *x*-*y*-*z*. Given the requirement on cortex-to-scalp di_s_tance *l*_s_ = 15 mm, the magnet should be placed at least 50 mm away from the robot along the *z*_r_-axis, i.e., *l*_mag_ ≥ 50 mm. All scale bars: 1 mm.

In this study, we propose a wireless stent-shaped magnetic soft millirobot that can achieve all of the above requirements to operate in the distal M4 segment of MCA (Fig. 1a). The robot achieves retrievably adaptive locomotion with the lumen diameter down to 1 mm, small lumen radius of curvatures down to 3 mm, branch bifurcation angles up to 120^°^, and pulsatile blood flow speeds up to 26 cm/s at 80 beats per minute (bpm). Moreover, the robot has a safe self-anchoring capability and can withstand the flow even when the external magnetic actuation input is turned off. In addition to controlled and precise navigation, we also demonstrate that the robot variants can have several essential medical functions. For example, the robot can deliver the tissue plasminogen activator (tPA) on-demand to achieve thrombolysis at the target location. Moreover, the robot can be used as diverters to regulate flow into undesirable sites, such as branches or aneurysms. These functions enable new minimally invasive targeted therapies for AIS, aneurysm, CAVM, dAVFs, and brain tumors in the distal and tortuous vascular regions.

## Results

### Design of the robotic system for distal-endovascular applications

We chose a cylindrical hollow stent shape for the magnetic soft robot design due to the radial deformability and low fluidic drag of such a shape^29,30^. This design ensures effective operation inside the arteries with the high-speed pulsatile flow and changing lumen diameter. Furthermore, to leverage rotational and translational magnetic actuation, we incorporated ferromagnetic NdFeB microparticles into the robot body and magnetized it uniformly along the *z*_r_-axis of the body-attached coordinate inside a vibrating sample magnetometer (VSM) with a homogeneous 1.8 T field (Fig. 1b and “Fabrication of the magnetic soft robots and quantification of Young’s modulus” in Methods).

To minimize the radial force applied to the lumen during locomotion, the radial stiffness of the overall robot *k*_r_ should be as small as possible. Meanwhile, the robot as a hollow structure tends to collapse itself due to internal magnetization-induced magnetic forces. Thus, *k*_r_ should also be high enough to prevent such undesired self-deformation. From the structural aspect, *k*_r_ is decided by three critical design parameters: the strut spacing *h*, the radius of curvature at the crown junction *ρ*, and the axial amplitude of each segment *f* (Fig. 1b). The structure employing larger *h*, *ρ*, and *f* enables smaller *k*_r_^29,30^. We decided these parameters based on the optimal design achieving the smallest *k*_r_ proposed by Bedoya et al. ^30^. The whole robot was composed of identical diamond-shaped cells. This arrangement enables uniform distribution of the compressive stresses and frictional forces when it deforms, minimizing the possibility of the robot twisting due to uneven contact force distribution, e.g., at bifurcation branches.

For locomotion inside the lumen, the helix structure can realize the axial propulsion when it converts rotational motion around the helical axis by magnetic torque to linear motion along the axis. This is induced by both helical shape and the anisotropic friction, where the coefficient of friction perpendicular is higher than the one parallel to the helix^24,31,32^ (Supplementary Table 2). To leverage such a property for locomotion, we designed right-handed helical structures with a helix angle *φ* of 8^°^ on the outer side of the robot as mechanical protrusions. Given the identical arrangement of the diamond-shaped cells, the helix-coated area along the robot is evenly distributed (Fig. 1b). The detailed design and measured parameters of the current prototypes can be seen in Supplementary Table 3.

**Fig. 2:**
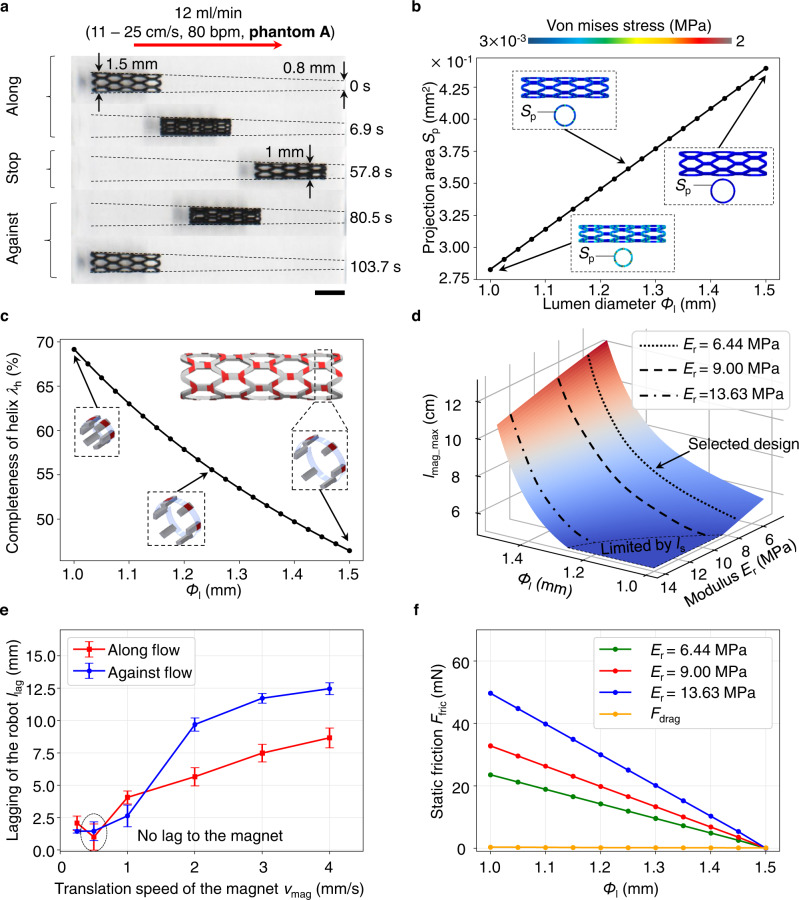
Radial shape adaptation and self-anchoring capability. **a** Snapshots for the retrievable radial shape adaptation in the lumen with diameter *Φ*_l_ changing from 1 mm to 1.5 mm (phantom A). Scale bar: 2 mm. Shape adaptation is quantified by the maximum allowed distance of the magnet away from the robot along the *z*_r_-axis enabling the locomotion, *l*_mag_max_. *l*_mag_max_ is decided by the projection area *S*_p,_ completeness of helix *λ*_h_, *Φ*_l_, and Young’s modulus *E*_r_. **b** Effect of *S*_p_ (*E*_r_ = 6.44 MPa). *S*_p_ is defined as the projected area of the robot to the *x*_r_-*z*_r_ plane. Higher *S*_p_ in smaller *Φ*_l_ tends to induce much increased fluidic drag *F*_drag_ on the robot. The radial deformability of the stent-shaped structure enables the shrinking of *S*_p_ into smaller *Φ*_l_. **c** Completeness of the discontinuous helix along with radial deformation. For one pitch, completeness is defined as the ratio between coated helix length (constant and discontinuous, indicated by red in the zoom-in figures) and the complete helix length (varying and continuous, indicated by red and blue in the zoom-in figures). The complete helix length is proportional to the diameter. For smaller *Φ*_l_, the completeness is improved, and the effect of anisotropic frictional forces for axial locomotion can be enhanced (Supplementary Fig. 5). **d** Modeling of *l*_mag_max_. Given the design with low fluidic drag and anisotropic frictional forces, *E*_r_ and *Φ*_l_ decide *l*_mag_max_. We chose the design with *E*_r_ = 6.44 since all *l*_mag_max_ was strictly greater than 50 mm. **e** Effect of the magnet translation speed *v*_mag_ on the robot displacement in phantom A (*f*_mag_ = 0.5 Hz, *l*_mag_ = 55 mm). The lagging distance to the length of phantom A indicates how well the robot could follow the magnet. *v*_mag_ at around 0.5 mm/s is suitable for actuation without lagging. The data are presented as mean values ± standard deviation for the number of trials *n* = 5. **f** Self-anchoring performance in phantom A. The frictional forces for robots with various *E*_r_ are higher than *F*_drag_, ensuring flow withstanding even when the external magnetic actuation is off.

To flexibly manipulate the robot for clinical applications, we combined a rotational cubic NdFeB permanent magnet (50 mm in length) and a seven degrees-of-freedom (DoF) robotic arm, realizing spatial three-dimension (3D) magnetic actuation (Fig. 1c, Supplementary Fig. 1, and “6-DoF magnetic actuation system” in Methods). Specifically, the magnet was rotated around the *y*_a_-axis with frequency *f*_mag_ (Fig. 1d − 1), translated ahead of the robot with a speed of *v*_mag_, and reorientated by an angle of *α*_mag_ to coincide with the *y*_a_-*z*_a_ plane to the current route (Fig. 1d − 2). Given the requirement on cortex-to-scalp distance *l*_s_^33,34^ of around 15 mm, the magnet should be placed at least 50 mm away from the robot along the *z*_r_-axis, i.e., *l*_mag_ ≥ 50 mm.

### Radial shape adaptation and self-anchoring capability

The first functional requirement, radial shape adaptation, demands a forward and backward (retrievable) surface locomotion capability in the lumen with a diameter *Φ*_l_ changing from 1.5 mm to 1 mm in the M4 region^3,35^. This *Φ*_l_ range corresponds to the average flow speed *v*_f_ estimated to be from 11.3 cm/s to 25.5 cm/s in a single route without branches^36^ (see “Physiological features of the MCA M4 segment and preparation of the simulants” in Methods). Particularly, this requirement needs to be fulfilled in two dynamic conditions, i.e., the magnet is actuated to move the robot along with and against the flow, for entering the smaller *Φ*_l_ and moving back to the larger *Φ*_l_, respectively.

To experimentally explore which design could fulfill such a requirement, we tested a class of robot prototypes with various Young’s moduli of *E*_r_ from 6.4 MPa to 13.6 MPa in phantom A made of poly(dimethylsiloxane) (PDMS) elastomer (Supplementary Fig. 2, see “Physiological features of the MCA M4 segment and preparation of the simulants” in Methods for details on the phantoms A – U). The magnet was rotated with the frequency of *f*_mag_ = 0.5 Hz and led the robot by half the size of the magnet (Supplementary Fig. 3). For radial shape adaptation to the narrower *Φ*_l_, the magnetic forces and torques need to be increased by decreasing the distance between the magnet and the robot along the *z*_r_–axis, *l*_mag_. Meanwhile, *l*_mag_ ≥ 50 mm needs to be assured for satisfying *l*_s_. Thus, the maximum allowed *l*_mag_ for adapting to various *Φ*_l_, *l*_mag_max_, dictates the capability of radial shape adaptation. The design with a larger *l*_mag_max_ is easier to achieve the shape-adaptive locomotion. Particularly, we found that the design with *E*_r_ = 6.4 MPa fulfilled the requirement with all *l*_mag_max_ greater than 50 mm (Fig. 2a and Supplementary Movie 1).

To explain the observed behaviors, we analyzed the relevant forces (see “Force modeling and analyses” in Methods). Notably, four essential variables, the projection area *S*_p,_ completeness of helix *λ*_h_, *Φ*_l_, and Young’s modulus *E*_r_, affect the relative relations among the magnetic forces and torques, fluidic drag, and frictional forces, which further dictates *l*_mag_max_.

First, to effectively withstand the pulsatile flow, the robot projection area *S*_p_, which is the projected area of the robot to the body-attached *x*_r_-*z*_r_ plane, should not increase when the robot enters a smaller *Φ*_l_, since increased *S*_p_ would increase the fluidic drag *F*_drag_ on the robot notably. Given the low fluidic drag nature of the stent shape, this requirement could be satisfied without further effort. Actually, *S*_p_ would decrease from 0.44 mm^2^ for *Φ*_l_ = 1.5 mm to 0.28 mm^2^ for *Φ*_l_ = 1 mm (Fig. 2b). Due to the increased flow speed in smaller *Φ*_l_, *F*_drag_ would still be increased from 0.1 mN to 0.3 mN. However, the increase is minor compared with another classic design for potential endovascular applications, the helical-shaped structure^24^ (Supplementary Fig. 4).

Second, in our specific robot design with a discontinuous right-handed helix, for one pitch, completeness *λ*_h_ is defined as the ratio between the coated helix length (constant and discontinuous, indicated by red in Fig. 2c) and the complete helix length (varying and continuous, indicated by red and blue in Fig. 2c). The complete helix length is proportional to the diameter. For smaller *Φ*_l_, *λ*_h_ is improved. Thus, the effect of symmetry breaking from the helical shape and the anisotropic frictions for the axial locomotion could be enhanced^24,31,32^, which aligns with the experiments (Supplementary Fig. 5). Although both left-handed and right-handed rotations enabled axial propulsion using our actuation method with the magnetic pulling force, the right-handed one improved the robot’s speed, especially significant when the robot moved from smaller *Φ*_l_ with higher *λ*_h_ to the larger *Φ*_l_ with lower *λ*_h_.

Given the design with low fluidic drag and anisotropic frictional forces, the effect of *E*_r_ and *Φ*_l_ on *l*_mag_max_ could be appropriately modeled (Fig. 2d). Either decreasing *Φ*_l_ or increasing *E*_r_ increased the necessity of increasing the magnetic force and torques, i.e., decreasing *l*_mag_max_ for the radial shape-adaptative locomotion. Particularly, the robot with *E*_r_ = 6.4 MPa fulfilled the requirement with all *l*_mag_max_ more than 50 mm, satisfying the distance requirement on cortex-to-scalp distance *l*_s_, which matched well with the experimental results (Supplementary Fig. 6a). Furthermore, modeling based on the ex vivo measurements of the coefficient of friction (CoF) for porcine arteries indicated that *l*_mag_max_ could be as large as 10 cm for the robot with *E*_r_ = 6.4 MPa, suggesting the feasibility of the current system design (Supplementary Fig. 6b).

Based on the modeling for arteries, the maximum radial forces *F*_n_ and frictional force *F*_fric_ for *E*_r_ = 6.4 MPa when the robot enters the lumen with *Φ*_l_ = 1 mm are estimated to be around 0.05 N and 0.004 N, respectively in porcine arteries. *F*_n_ here induces the compressive stress of around 5.5 kPa, smaller than the quantified threshold to rupture the endothelial cell at around 12.4 kPa^37,38^. Moreover, these values are in the same order as the one achieved by the novel braided stent used as the blood clot retriever, which has been shown to decrease the radial force to minimize the damage to the endothelial layer^39^. Thus, this design can potentially enable safer interactions with the lumen. The following analyses on other locomotion modes in PDMS-based phantoms focused on this design.

Previously, the magnet was controlled by the manual operation command from the joystick to investigate whether or not the robot could achieve the radial shape adaptation. We further studied how fast the adaptation could achieve. We fixed *l*_mag_ = 55 mm since this value could enable the radial shape adaptation for all *Φ*_l_ (Supplementary Fig. 6a). Then we investigated the effect of various translation speeds of the magnet *v*_mag_ and frequencies *f*_mag_. The studies in phantom B and the corresponding modeling indicated that *f*_mag_ did not contribute to *v*_r_ as much as *v*_mag_ (Supplementary Fig. 7). Therefore, we fixed *f*_mag_ as 0.5 Hz. The robot was further tested in phantom A with different *v*_mag_ from 0.25 mm/s to 4 mm/s (automatic mode, and the pulsatile flow rate was set at 12 ml/min). The experimental results were summarized in Fig. 2e and Supplementary Fig. 6c. When *v*_mag_ was set to be 0.5–1 mm/s, the robot followed the magnet translation with no lag, with the average locomotion speed *v*_r_ of around 0.18 mm/s (along and against the flow on average). Note that due to the variation of the magnetic forces and torques, *v*_r_ was not constant, and we reported the average values along the path.

Besides the radial adaptation, the robot is also required to be self-anchoring to withstand the pulsatile flow even when the magnetic field is turned off, which is essential for safe operations. To enable such a property, the frictional forces *F*_fric_ should be larger than the fluidic drag *F*_drag_. The experiments and modeling results indicated that this requirement could be satisfied for all designs (Fig. 2f). The relation also held for arteries, given the quantified CoF (Supplementary Fig. 6d).

The current robot design was compared with the relevant works in the literature^18–22,24–27^, as indicated in Supplementary Fig. 8. Our design shows superiority in the flow withstanding speed range and the self-anchoring mechanism for broader future endovascular applications.

### Locomotion among highly curved routes

**Fig. 3:**
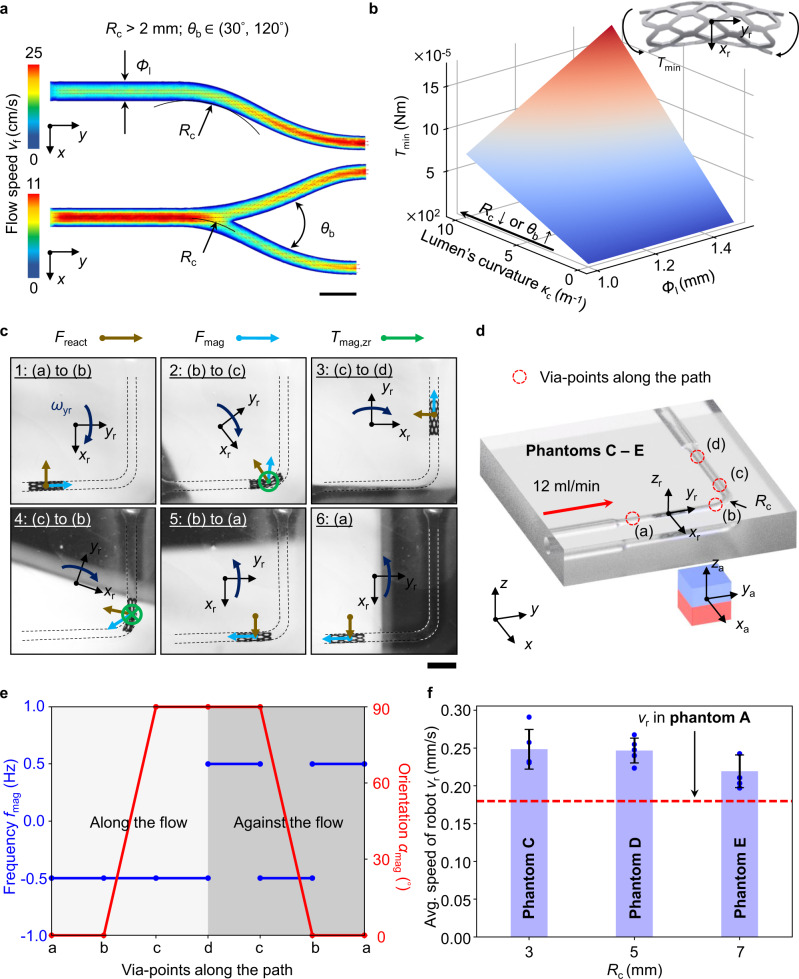
Locomotion among highly curved routes. **a** Physiological requirements on curved lumens with flow speed visualization (including curved routes and bifurcations). The values of radius of curvature *R*_c_ and bifurcation angles *θ*_b_ are indicated in the figure. Scale bar: 2 mm. **b** Effect of lumen’s curvature *κ* and *Φ*_l_ on the minimum required torque to bend the robot into the curved lumens *T*_min_ (*E*_r_ = 6.44 MPa). Increasing *κ* (more curved lumens with smaller *R*_c_ or larger *θ*_b_) and *Φ*_l_ increases *T*_min_. (**c**) Snapshots for retrievable curved routes traversing with *R*_c_ of 5 mm. The bending torques from the magnetic force *F*_mag_ due to the leading position of the magnet, magnetic torque *T*_mag,z_ due to the reorientation of the magnet, and the reaction force *F*_react_ from the lumen wall due to the rotation of the magnet, overcome *T*_min_, realizing the curved route traversing. Scale bar: 4 mm. Only the forces and torques enabling the traversing are labeled. **d** Coordinate system and via-points discretization for curved routes traversing. **e** Strategies for curved routes traversing (+ and – represent the rotation direction of the magnet around the *y*_a_-axis). **f** Experimental results of robot locomotion speed among various *R*_c_ in phantoms C – E (*f*_mag_ = 0.5 Hz, *l*_mag_ = 55 mm). The results are consistently around 0.18 mm/s, the one acquired from phantom A. Thus, traversing among curved routes does not sacrifice the locomotion speed. The data are presented as mean values ± standard deviation for the number of trials *n* = 5.

For traversing among highly curved lumens, i.e., the curved routes and bifurcations (Fig. 3a), the robot needs to be bent around the *z*_r_-axis. The minimum required torque for such bending *T*_min_ is correlated to the lumen’s curvature *κ*_c_ and *Φ*_l_, i.e., *T*_min_ = *E*_r_·*I*_r_(*Φ*_l_)·*κ*_c_, where *I*_r_ is the second moment of area for the cross-section along the *x*_r_-*z*_r_ plane (Fig. 3b). Note that either increasing *κ*_c_ (decreasing the radius of curvature *R*_c_ or increasing the bifurcation angle *θ*_b_) or increasing *Φ*_l_ increases *T*_min_. We focused on *Φ*_l_  = 1.45 mm to experimentally find the effective curved routes traversing strategies in the physiologically relevant *R*_c_ (≥1 mm) and *θ*_b_ (30^°^–120^°^) value ranges (see “Physiological features of the MCA M4 segment and preparation of the simulants” in Methods). Particularly, for consistent evaluation of the strategies for curved routes, the inclination angle *γ*_i_ for each phantom route was unified as 90^°^.

The total torques applied to the robot need to be greater than *T*_min_ to bend the robot into the curved route. These contributing torques include: 1) the torque from the magnetic pulling force, *F*_mag_, due to the leading position of the magnet, 2) the magnetic toque around the *z*_r_-axis, *T*_mag,zr_, due to the reorientation of the magnet, and 3) the reaction force from the lumen wall due to the robot rotation, *F*_react_ (Fig. 3c).

To explain the actuation strategies, we discretized the locomotion path into via-points (a) to (d), shown in Fig. 3c, d, and Supplementary Movie 2. When the robot entered the curved route and moved to path (c) – (d), the magnet was oriented such that the *y*_a_-axis was parallel to (c) – (d), leading the robot and rotating the robot around the positive *y*_r_-axis simultaneously. Thus, the torque from the magnetic pulling force *F*_mag_ bent the robot towards (c) – (d). Meanwhile, *T*_mag,zr_ tended to align *x*_r_ to *x*_a_, which bent the robot towards the same direction. Lastly, the rotation tended to roll the robot to the positive side of the *x*_r_-axis. When the robot was in contact with the lumen wall, the reaction force *F*_react_ bent the robot towards (c) – (d) (see Supplementary Notes S1 and S2). The consequent sum of the bending torques overcame *T*_min_ such that the robot could enter (c) – (d). The procedures above are indicated in step 2 in Fig. 3c.

When the robot returned to (a) – (b) from (c) – (d), the magnet was oriented such that the *y*_a_-axis was parallel to (a) – (b), leading the robot and rotating the robot around the positive *y*_r_-axis. Thus, the torque from pulling force *F*_mag_ bent the robot towards (a) – (b). Meanwhile, *T*_mag,zr_ tended to align *x*_r_ to *x*_a_, which also bent the robot towards (a) – (b). Lastly, the rotation tended to roll the robot to the positive side of *x*_r_-axis. When the robot was in contact with the lumen wall, the reaction force *F*_react_ would point to and bend the robot towards (a) – (b). The above procedures are indicated in step 4 in Fig. 3c. The complete strategies for the whole path are summarized in Fig. 3e. For detailed quantification of the contributing torques, please refer to Supplementary Note 2.

The effectiveness of the proposed strategies was evaluated using the phantoms C-E, and the experimental results are consistent. The average robot speed *v*_r_ was around 0.2 to 0.25 mm/s, indicating the robustness of the strategies among different curved routes (Fig. 3f). Furthermore, these values are similar to *v*_r_ acquired from the investigation on phantom A (0.18 mm/s), suggesting that traversing among curved routes does not sacrifice locomotion efficiency.

### Locomotion among bifurcating branches

**Fig. 4:**
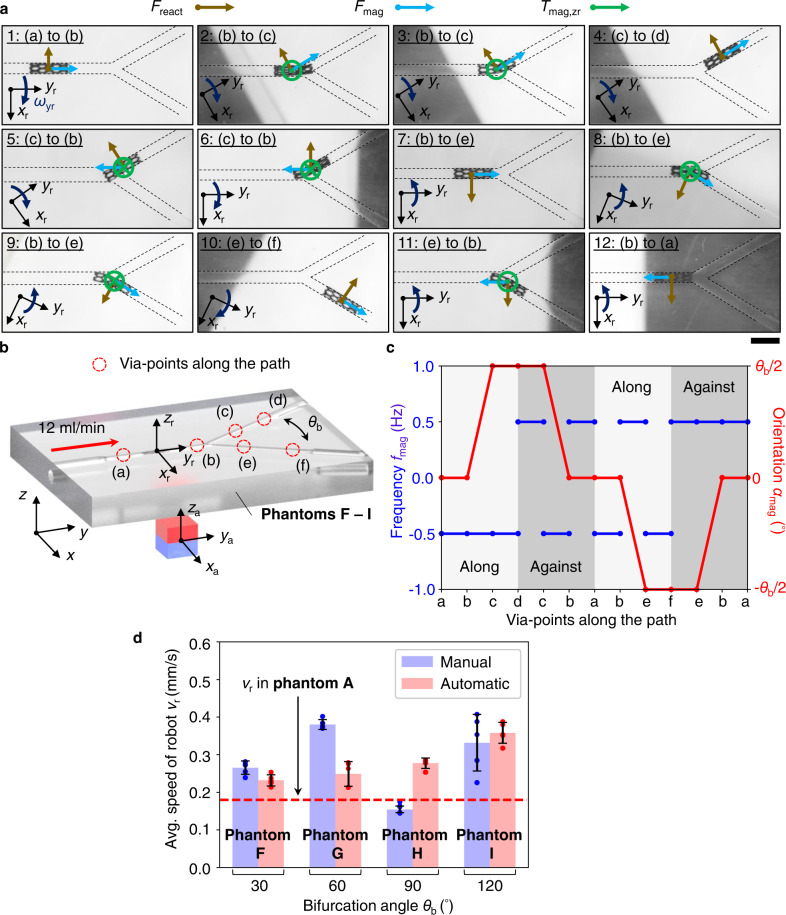
Locomotion among bifurcating branches. **a** Snapshots for retrievable branch traversing among the bifurcation with angle *θ*_b_ of 60^°^. Only the leading forces and torques enabling the traversing are indicated on each time step. The composition of magnetic force-induced torque from *F*_mag_, magnetic toque *T*_mag,zr_, and reaction force-induced torque from *F*_react_, bends and leads the robot into the desired branch. Scale bar: 4 mm. **b** Coordinate system and via-points discretization. **c** Strategies for branch traversing. **d** Experimental results of robot locomotion speed among various bifurcation angles in phantoms F – I from manual control and automatic trajectory (*f*_mag_ = 0.5 Hz, *l*_mag_ = 55 mm). The results are around 0.18 mm/s, the one acquired from phantom A. Thus, traversing among bifurcations does not sacrifice the locomotion speed. The data are presented as mean values ± standard deviation for the number of trials *n* = 5.

The strategies for bifurcating branch traversing are similar to the ones for curved routes. For the bifurcation angle *θ*_b_ from 30^°^ to 120^°^, the contributing torques applied to the robot should be greater than *T*_min_. To explain the strategies, we discretized the path into via-points (a) to (f), shown in Fig. 4a, b, and Supplementary Movie 3. The procedures for the robot entering into the branch (c) – (d) can be indicated from steps 2 and 3 in Fig. 4a. The magnet was oriented such that the *y*_a_-axis was parallel to the route (c) – (d), leading the robot and rotating the robot around the positive *y*_r_-axis simultaneously. Thus, the torque from pulling force *F*_mag_ bent the robot towards (c) – (d). Meanwhile, *T*_mag,zr_ tended to align *x*_r_ to *x*_a_, which bent the robot towards the same branch. Lastly, the rotation tended to roll the robot to the positive side of the *x*_r_-axis. When the robot was in contact with the lumen junctions, the reaction force *F*_react_ would point to the negative *x*_r_-axis, bending the robot towards (c) – (d) (see Supplementary Notes 1 and 3). The consequent sum of the bending torques is greater than *T*_min_, so the robot could enter (c) – (d).

The procedures for the robot returning to the main trunk (a) – (b) from (c) – (d) could be indicated in steps 5 and 6 in Fig. 4a. The magnet was oriented such that *y*_a_-axis was parallel to the route (a) – (b), leading the robot and rotating the robot around the positive *y*_r_-axis. Thus, the torque from pulling force *F*_mag_ bent the robot towards (a) – (b). Meanwhile, *T*_mag,zr_ tended to align *x*_r_ to *x*_a_, which also bent the robot towards (a) – (b). Lastly, the rotation tended to roll the robot to the positive side of *x*_r_-axis. When the robot was in contact with the lumen wall, the reaction force *F*_react_ would point to the negative *x*_r_-axis, bending the robot towards (a) – (b). Similar strategies could be extrapolated when the robot enters the branch (e) – (f) and returns. The complete strategies for the whole path are summarized in Fig. 4c. For detailed quantification of the contributing torques, please refer to Supplementary Note 3.

To investigate the consistency of the proposed strategies, we evaluated them in phantoms F-I by manual trajectories, i.e., manipulation by joystick, and automatic ones, i.e., manipulation by via-points from the predefined trajectories. The automatic ones were used to rule out the randomness in manual control. The average locomotion speed *v*_r_ was 0.15–0.35 mm/s, which are relatively consistent among the different phantoms with various *θ*_b_ and different modes (manual or automatic, Fig. 4d). These values are similar to the one acquired from the investigation on phantom A (0.18 mm/s), indicating that traversing among the branches did not notably sacrifice locomotion efficiency. However, the values of *v*_r_ are not exactly the same due to the fabrication differences on various robots and phantoms. As shown above, the curved lumen traversing is enabled by the magnetic forces, magnetic torques, and interaction with the lumen walls. Given the same magnetic actuation configuration and the similar elastic properties of phantoms and the arteries, the strategies can be expected to be transferred to real artery cases.

### Locomotion demonstrations and robot detection under medical imaging modalities

**Fig. 5:**
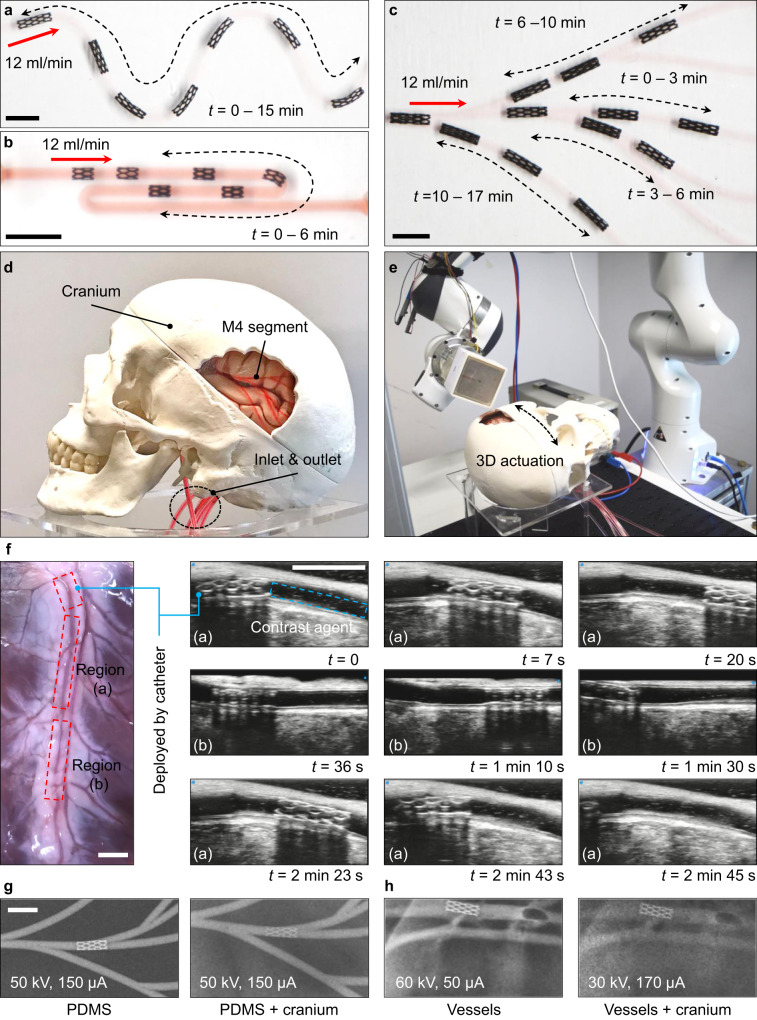
Locomotion demonstrations and robot detection under medical imaging modalities. In all the demonstrations, the pulsatile flow rate into the inlet was 12 ml/min. **a** Locomotion in a tortuous route (phantom J). The radius of curvature *R*_c_ range is from 2.5 mm to 5 mm. **b** Locomotion in an extremely curved route where the inclination angle *γ*_i_ is 180^°^, and *R*_c_ is 1 mm (phantom K). **c** Locomotion among branches in 2D (phantom L). **d** and **e** Locomotion among 3D branches in a cranium simulant (phantom M). **f** Robot delivery using a medical catheter and locomotion in porcine arteries visualized by ultrasound imaging. At *t* = 1 min 10 s, the magnet was moved away, but the robot could withstand the flow safely. **g** Optimal X-ray imaging parameters for robot detection in PDMS phantoms with and without cranium simulant as the cover (all with contrast agent). **h** Optimal X-ray imaging parameters for robot detection in vessels with and without cranium simulant as the cover (all with contrast agent). All scale bars: 5 mm.

The robot’s locomotion capability was demonstrated in the phantoms with combined physiological features based on the above knowledge. The first demonstration was performed in phantom J with the continuous tortuous route, where the radius of curvature *R*_c_ is from 2.5 mm to 5 mm. The lumen diameter *Φ*_l_ is set to be 1.45 mm (Fig. 5a). Note that the current arm configuration enabled the magnet’s orientation up to ±90^°^ around the global *z*-axis (Supplementary Fig. 1). For a rather tortuous route, reorientation of the phantoms was made to assist the robot locomotion if the arm manipulation came to a singular configuration (Supplementary Movie 4). The second demonstration was made in phantom K with the highly curved route, i.e., 180^°^ for the inclination angle (*γ*_i_ = 180^°^) and *R*_c_ = 1 mm. The phantom was based on an angiography example for the posterior divisions of the MCA reported in the literature^40^, where we adapted the original *γ*_i_ = 110^°^ and *R*_c_ = 3 mm to demonstrate the accessibility of the current robot design. Here the robot with two cells along the axis showed flexible navigation in this very special condition (Fig. 5b and Supplementary Movie 5).

The third demonstration was managed in phantom L with six branches, where *Φ*_l_ was designed to be from 1 mm to 1.5 mm (Fig. 5c and Supplementary Movie 6). Finally, the third demonstration was carried out in phantom M, the 3D version of phantom L, fixed on the cerebral hemisphere simulant (Fig. 5d, e, and Supplementary Movie 7; human skull model with the brain, 3B Scientific GmbH). The successful locomotion among these phantoms confirmed proper understanding of mechanics and effective system development towards the shape-adaptative locomotion in complex lumens of various *Φ*_l_ with the pulsatile flow.

To evaluate the robot in more medically relevant settings and demonstrate the scenario of its advantage against the catheters, we delivered the robot by medical tubing and tested its locomotion in fresh porcine arteries with *Φ*_l_ from 1 mm to 1.5 mm, also with the pulsatile flow pumped inside. The vessels we used were the coronary arteries freshly cut from the porcine hearts (Fig. 5f and Supplementary Movie 8) (see “Preparation of the porcine coronary arteries and thrombus for ex-vivo tests” in Methods). The blood analog with 12 ml/min was pumped into the arteries during the experiment. The robot was first delivered to the target region’s vicinity by a catheter (inner diameter ID = 0.072 in, Polyurethane Medical Tubing, Nordson Medical). Then, the robot was released to the locomote to the region that the catheter could not access. Ultrasound imaging was utilized to visualize the robot locomotion in the artery^20,41^ (see “Collection of visual data” in Methods). The pulsatile flow was visualized by a contrast agent added to the blood analog (Vevo MicroMarker Non-Targeted Contrast Agent, FUJIFILM Visualsonics, Inc) (Supplementary Fig. 9). The proof-of-concept demonstration indicates how the robot can be compatible with the existing endovascular tools and improve their accessibility. Thinner microcatheters should be chosen for practical usage in distal segments^3^, e.g., Headway 27^TM^ (tip ID: 0.027 in). The robot with different initial diameters can be fabricated and delivered by these microcatheters based on application needs. For example, when towards flow diversions, the robot does not need to carry other therapeutic agents, so it can be collapsed enough to be carried and delivered by the microcatheters, as shown in the next section.

On the other hand, the robots need to carry other functional tools when delivering therapeutic agents. Therefore, the robot should not be collapsed too much, and the initial diameter needs to fit the ID of the microcatheter. To confirm such a capability, we even fabricated a smaller-diameter robot with an outer diameter of 0.7 mm, compatible with a microcatheter with ID = 0.03 in. Both experimental results and the theoretical modeling indicated that retrievable locomotion for *Φ*_l_ = 1.5 mm to 0.3 mm could be achieved, and the self-anchoring capability was maintained for *Φ*_l_ = 0.7 mm to 0.3 mm (Supplementary Note 4).

The robot was also tested under X-ray imaging, commonly used in minimally invasive endovascular surgeries. The robot’s detection under the X-ray imaging (see “Collection of visual data” in Methods) was managed under various conditions, e.g., 1) the robot inside a PDMS phantom, 2) the robot inside the PDMS covered by a cranium simulant, 3) the robot in porcine vessels, and 4) the robot in vessels covered by a cranium simulant. The contrast agent (Iomeron 400, solution for injection, Bracco UK Limited) in PBS was also injected into the lumens (mass ratio 1:1). Two critical imaging parameters, voltage and current, were swept to find the best imaging results (Fig. 5g, h; please refer to Supplementary Fig. 10 for more details on the effects of these parameters). The clear identification of the robot from the background materials supported its efficacy in potential future clinical usage for human inspection-based intervention or robotic surgeries.

### Proof-of-concept local on-demand drug delivery and flow diversion

**Fig. 6:**
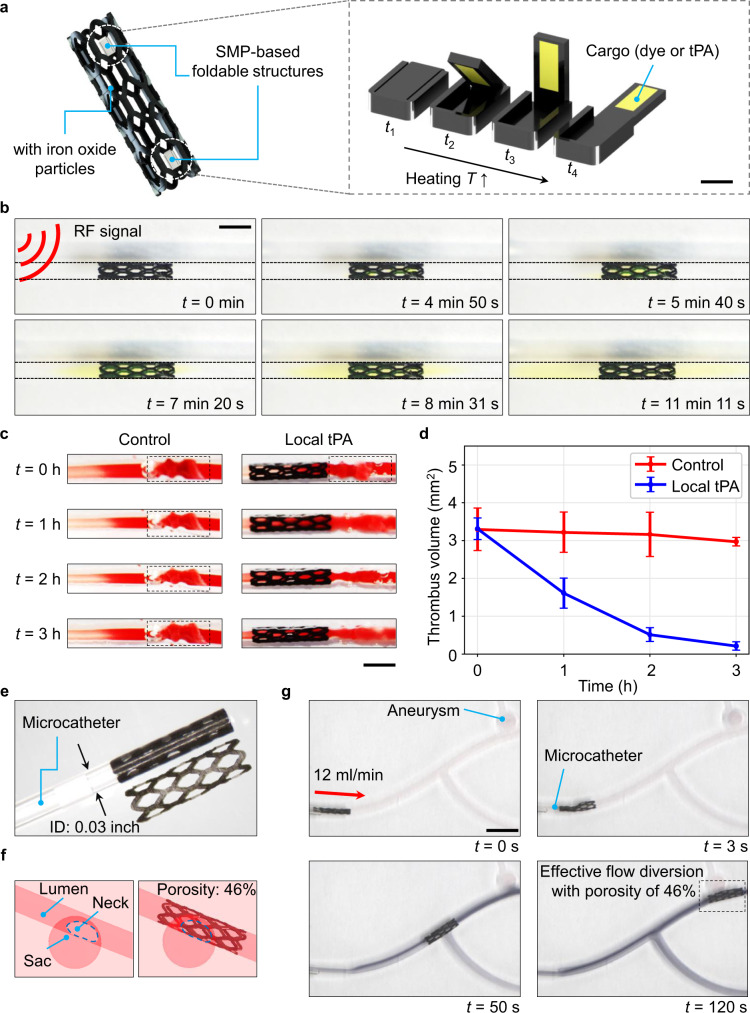
Proof-of-concept local on-demand drug delivery and flow diversion. **a** A design variant with the SMP-based foldable structures for local on-demand delivery of the endovascular tissue plasminogen activator (tPA) towards the therapy of acute ischemic stroke. Scale bar: 0.5 mm. **b** Demonstration of the radiofrequency-based heating and release of the composite cargo consisting of silk fibroin and fluorescein dye. Scale bar: 2 mm. **c** Effect of thrombolysis using the carried tPA. The thrombi are labeled by black dotted lines. Scale bar: 2 mm. **d** Quantitative results on the effect of thrombolysis. The data are presented as mean values ± standard deviation for the number of trials *n* = 3. **e** Compatibility of the robot with microcatheter for endovascular flow diversion towards the aneurysm and arteriovenous malformation therapies. **f** Diagrams for the components of saccular-shape aneurysm and computation of porosity to be 46% for the neck size of 0.44 mm^2^. The porosity, measured on the patch covering the neck as the ratio between the area of voids and the total area, indicates flow diversion efficacy. When the porosity is less than 70%, the flow condition is more favorite to initialize the thrombus formation and promote the aneurysm occlusion^46,47^. **g** Experimental demonstration on the flow diversion of the robot delivered by the microcatheter (ID: 0.03 in) and magnetically controlled to the desired lesion site in phantom N. Scale bar: 5 mm.

Given its promising locomotion capability in distal artery lumen environments under pulsatile flow conditions, the stent-shaped magnetic soft robot can function as a wireless medical device. Particularly, here we demonstrated two proof-of-concept functions that the robot could realize local on-demand drug delivery and flow diversion, potentially enhancing the current state-of-the-art catheter-based therapies for AIS, aneurysm, and AVM in the distal arteries (Fig. 6).

For AIS in the middle cerebral artery, the large vessel occlusions in the proximal M1 and M2 are successfully treated with intra-arterial mechanical thrombectomy. However, given the tortuosity, narrower lumen diameters, and deeper territory in the distal M3 and M4 regions, the therapies for the occlusions there are not standardized with mechanical thrombectomy^3,7^, where the intravenous or intra-arterial thrombolytics can be applied^7^. Intravenous thrombolytics fail to recanalize one-half to two-thirds of these distal occlusions^3^. Meanwhile, catheter-based intra-arterial thrombolytics are still limited by accessibility^8,42^. To enhance the thrombolysis in these distal vascular regions, we propose using our wireless soft robot to locally deliver the tissue plasminogen activator (tPA). This method is promising for improving the effectiveness of such medicine since the releasing location and quantity can be actively controlled. Moreover, the targeted application of a drug can improve the local concentration, thus minimizing the systematic exposure and reducing undesired complications^43^.

To achieve this goal, we designed shape memory polymer (SMP)-based foldable structures, which could hold the tPA inside and release it through remote radiofrequency (RF) heating (Fig. 6a) (see “SMP-based foldable structure for cargo (drug) loading and releasing” in Methods). The demonstration of the working principle is indicated in Fig. 6b and Supplementary Movie 9 using fluorescein dye (Fluorescein sodium, Fisher Scientific U.K. Limited). The RF coil was placed 20 mm away from the robot (also satisfies *l*_s_), which contained iron oxide (Fe_3_O_4_) nanoparticles to enhance the efficiency of RF heating (PDMS:NdFeB:Fe_3_O_4_ = 1:3:1 for the mass ratio). The maximum power used for RF heating was 752 A with 337 kHz (EASYHEAT, Ambrell Corporation).

After heating for around 5 mins, the SMP reached its shape-memory transition temperature of 30 ± 5 °C, and the structures opened. Note that the transition temperature could be tuned precisely up to 90 °C using the method adopted in this work, promising for actual clinical usage where the average human body temperature is 37 °C^44^. After the SMP structures opened, the capsulated solid pill, made of silk fibroin and fluorescein dye, was dissolved into the deionized water, which could be visualized clearly via the diffusion of the yellow color.

To confirm the efficacy of the local on-demand drug delivery, around 0.02 mg tPA (Actilyse^®^, Boehringer Ingelheim International GmbH) was released in the vicinity of the fabricated blood thrombi with volumes of around 3.5 mm^3^ (see “Preparation of the porcine coronary arteries and thrombus for ex-vivo tests” in Methods). The samples were kept at a constant temperature of 37 °C, where the thrombolysis results are shown in Fig. 6c, d. The comparison with the control group without the drug shows the effectiveness of the local application of tPA. Clinically, the robot could be immediately retrieved after the drug is released to the thrombus.

For aneurysm and AVM in the distal neurovascular routes, the current minimally invasive therapies dependent on catheters are also limited by the accessibility into the vicinity of the lesioned regions and the flexibility of the delivered therapeutic agents^9,11,45^. We demonstrated that our robot could function as an actively controlled, flexible stent-like flow diverter into these distal regions. To achieve so, we first collapsed the robot enough to fit into the microcatheter compatible with the distal M3 segment (ID = 0.03 in, Polyurethane Medical Tubing, Nordson Medical)^3^ (Fig. 6e). Given its *E*_r_ of 6.44 MPa compared with the Nitinol conventional flow diverter of 28 – 83 GPa, the collapsed robot should be flexible enough to be delivered up to the end of the M3 segment. Next, the robot can be released and magnetically controlled to the even distally tortuous site to divert the flow. Particularly, for a saccular aneurysm with a neck size of 0.44 mm^2,10^, our robot can achieve a porosity of 46%, which is promising to trigger the effective occlusion^46,47^ (Fig. 6f). The porosity, measured on the patch covering the neck as the ratio between the area of voids and the total area, indicates flow diversion efficacy. Previous quantified investigations have shown that when the porosity is less than 70%, the flow condition is more favorite to initialize the thrombus formation and promote the aneurysm occlusion^46,47^. However, further investigations are needed to fully understand the robot’s efficacy for saccular aneurysms with various physiological features. The complete procedures indicated above can be referred from Fig. 6g and Supplementary Movie 10, which was carried out in phantom N.

## Discussion

Given the limited accessibility of catheterization into the distal vascular routes, we designed magnetically-driven wireless stent-shaped soft machines and investigate their actuation strategies. The device has shown substantial advantages in the following aspects. First, in respect of accessibility, its controlled retrievable locomotion improved the maneuverability into the distal regions with the self-anchoring assurance given flow or no flow in the lumen. Second, it enabled safe interaction forces, where lower forces were applied to the vessel walls during its intervention (max. on the order of 10^−2^ N) when compared to the standard neuro-interventional catheterization (max. on the order of 10^0^ N). Third, its local drug delivery capability would minimize the possible systematic side effects. Finally, its position could be adjusted actively if misplacement or dislodgement^48^ during the endovascular operations or migrations after the surgeries occurs^49–51^, e.g., as flow diverters to treat wide-neck aneurysms. For the detailed point-to-point experimental justifications of these advantages, please refer to Supplementary Note 5.

Besides the demonstrated medical functions, with its superiority in local on-demand drug delivery, the robot can avoid the risks and complications of the second systematic exposure to tPA. Clinically, after the first intravenous thrombolytic attempt does not achieve the desired recanalization, it is unsafe to expose the whole vascular system to tPA again immediately. This is due to the risks of internal hemorrhage from the repetitive, systematic exposure to the medicine^3^. Thus, the proposed robot here can function as a solution for such a scenario. Furthermore, the customized concentrated drug can be developed and loaded into the robot to improve its therapeutic efficiency. For example, we demonstrated that the local application of 0.02 mg tPA could enable successful thrombolysis for thrombus with a volume of 3.5 mm^3^. However, the concentration of the active gradients, alteplase, is only 2.1% in the commercial Actilyse^®^ (467 mg tPA contains 10 mg alteplase). Thus, an even smaller amount of drug is needed with a more concentrated alteplase. In addition, other functional medical tools, e.g., miniature flow pumps realized by cilia-like structures, can also be appropriately integrated into the robot to enhance drug delivery efficiency further^52^. Last but not least, the robot usage could also be potentially extended to the proximal arteries, given the enlarged magnetic moment of the robot in the larger lumen diameters and the low coefficients of friction in the arteries. As a controllable mobile flow diverter, the robot can be also wirelessly and precisely adjusted if misplacement, dislodgement, or migration occurs. For experimental validations, please refer to Supplementary Notes 6 and 7.

The overall concept of the mobile magnetic stent-shaped soft machine can be combined with state-of-the-art microcatheter techniques in distal vascular systems for a systematic improvement of clinical effectiveness. However, most of the tests were conducted in realistic phantoms to understand the detailed mechanics of soft-bodied adaptive locomotion systematically. More quantitative analyses of the robot in specific arteries are needed to assist the region-oriented applications. Second, the range of shape adaptations can be potentially boosted by utilizing active programmable magnetic materials to translate the robot application from minimally invasive to almost non-invasive surgeries^27^. Third, the materials used in the current prototype do not satisfy the biocompatibility and hemocompatibility of a blood-contacting device at the same time^53–57^. To improve these two critical aspects, we first coated the NdFeB microparticles with silica following the method developed in a recent study^1^ to provide an extra protective shell preventing corrosion. Then, we coated the two composite materials, e.g., PDMS + NdFeB@SiO_2_ (for the robot body) and SMP + Fe_3_O_4_ (for the foldable structure), with Parylene C (SCS Labcoter^®^ 2 (PDS 2010), Specialty Coating Systems). Parylene C, as an FDA-approved biocompatible and hemocompatible material, conforms to the stringent USP Class VI, ISO 10993, and RoHS standards with four decades of usage in implantable and non-implantable medical devices, including the blood contact stents, guidewires, and catheters. We experimentally optimized the thickness of the Parylene C coating layer and the modulus of the robot to confirm that the locomotion capabilities and functions could be maintained properly (see “Evaluation of biocompatibility” and “Evaluation of hemocompatibility” in Methods and Supplementary Note 8). Since the proposed robot design is not limited to a particular material, we anticipate that using the systematic experiments and modeling in this study, the robot can be fabricated with various FDA-approved materials for medical devices, such as polyurethane, polyethylene, and even metals^58,59^, for various application scenarios as future work. Last but not least, descriptive force modeling was carried out to supplement the experimental studies and used to explain the phenomenon in the current work. However, the transition dynamics during robot locomotion among complex structures, e.g., curved routes and branches, under the pulsatile flow conditions, needs to be further investigated for the reliable usage of the robot in vivo. These systematic understandings and improvements would advance the medical robotics field and translate the proposed functional wireless robot to the clinical side.

## Methods

### Physiological features of the MCA M4 segment and preparation of the simulants

The MCA originates at the internal carotid artery (ICA) bifurcation. The M4 segment, with a lumen diameter *Φ*_l_ of around 1–1.5 mm, is regarded as the medium vessel category and the distal segments of MCA. It starts when the vessels exit the Sylvian fissure and spread out over the convex surface of the cerebral hemispheres^3,35^. The angle for bifurcation *θ*_b_ in this segment varies, and we selected the representative range from 30^°^ to 120^°^ to cover the data reported in the literature^40^. The radius of curvature *R*_c_ for the vessels here is reported to be larger than 2 mm^40^. Without the loss of generality, we used the reported data on the average scalp-to-cortex distance to describe the distance between the M4 segment and the outer side of the scalp *l*_s_, around 15 mm on average^33,34,60^.

Based on the above geometrical features, we prepared two groups of phantoms, i.e., the quantitative phantoms for evaluating robot performances and the ones for demonstrations. The detailed design parameters of these physiologically relevant phantoms A – U are given in Supplementary Table 4. All phantoms were made by PDMS elastomer (Sylgard^TM^ 184, Dow Inc.). PDMS was chosen for its mechanical stability and wide acceptance in biomedical applicationss^61^. The injection molding technique was used to fabricate the desired lumen geometries. The negative molds (two parts) for the desired shape of the lumens were firstly 3D-printed (Clear V4 resin, FormLab Form 3). Commercial cast wax (Salmue) was then melted at 120 °C and injected into the molds by syringe. The solidified wax with the desired lumen shapes was taken out. Next, PDMS (mass ratio 10:1) was poured into and cured at room temperature (23 °C) for 48 hours. The phantoms were then placed in the hot oven at 120 °C, and the molding wax was melted away. Finally, the cured PDMS phantoms with the hollow lumen structures were placed in the ultrasound cleaner and cleaned with ethanol for 5 hours, which removed the wax residuals. Young’s modulus of PDMS phantoms is around 2.6 MPa^62^, achieving similar values as arteries at around 1–3 MPa^63,64^. However, the friction properties, e.g., the coefficients of friction (CoF), do not generally match (see “Quantification of the friction properties for the porcine arteries and PDMS phantoms”). Thus, we developed a customized model to show the robot can realize desired locomotion functions in arteries in each relevant section. The successful locomotion demonstration in the porcine artery, as shown in Fig. 5f, also justified the practical usage of the robot design.

The blood analog for all the quantitative analyses in PDMS phantoms was the glycerol/deionized water mixture, and the volume ratio is 44 to 65. The mixture has a dynamic viscosity of 4.4 cP at room temperature 23 °C, matching human blood for normal control subjects and moderate normal artery shear rates at 37 °C, i.e., 4.4 ± 0.5 cP^65,66^ (Supplementary Table 5). For all ex-vivo demonstrations in organs, Phosphate-buffered saline (PBS, pH = 7.4, Gibco^™^, Thermo Fisher Scientific) was pumped to the porcine coronary arteries to clean the route first, and then the blood analog was used for the tests.

Concerning the flow rate in brain arteries, the total cerebral blood flow (717 ± 123 ml/min) distributed to one side of the MCA is 21%, with 6% supplied to the distal MCA^36,67^. This calculates around 4.5–7 ml/min of flow rate going into each part of the M4 segment. We currently set the flow rate of the M4 segment to be around 10–12 ml/min for quantitative analysis and modeling. The average resting heart rate for adults ranges from 60 to 100 bpm, and we unified the stroke rate to 80 bpm. We used a commercial pulsatile blood pump (Harvard Apparatus) to pump 10–12 ml/min blood analog with 80 bpm in all the experimental conditions with the flow.

### Quantification of the friction properties for the porcine arteries and PDMS phantoms

The coefficient of friction (CoF) between the robot and the porcine arteries was settled using the fresh porcine aorta and the friction tests by the mechanical tester (Instron 5942). The aorta samples were cut and immersed into the PBS in the customized holders. The pulling direction for the measurement was parallel and perpendicular to the outer helix structure of the robot, respectively. The tests gave the average maximum static CoF along the helix *µ*_sa,∥_ = 0.12, the average maximum static CoF perpendicular to the helix *µ*_sa,⊥_ = 0.18, the average kinetic CoF along the helix *µ*_ka,∥_ = 0.07, the average kinetic CoF perpendicular to the helix *µ*_ka,⊥_= 0.08. The measurements on kinetic CoF align with the reported values in the literature, where the CoF between stent devices and the endothelial cells of artery lumens could vary from 0.03 to 0.06^68^.

The same tests were managed between the helix structure and the phantom material PDMS. PDMS is known for its hydrophobicity, increasing the friction between the robot and the phantom lumen wall and hindering locomotion. Therefore, plasma treatment was used to render the PDMS surface hydrophilic before the experiments^61,69^. The PDMS phantoms were put into the plasma cleaner and treated with the power of 75 W for 3 mins by air (Tergeo, PIE Scientific LLC). The sample was then immediately immersed in the blood analog for experiments and tests. Due to the phenomenon of stick-slip friction of PDMS^70^, we quantified the maximum static CoFs for the samples and used them for modeling and analyses, where the average maximum static CoF along the helix *µ*_sp,∥_ = 0.38, the average maximum static CoF perpendicular to the helix *µ*_sp,⊥_ = 0.47. For details, please refer to Supplementary Table 2.

### Fabrication of the magnetic soft robots and quantification of Young’s modulus

The positive robot model was firstly 3D-printed (IPQ, Nanoscribe GmbH), which was used to fabricate the negative PDMS mold (base and crosslinker mass ratio 10:1). The composition for casting was PDMS with the various mass ratios of 3:1, 7:1, and 12:1, and the Neodymium-iron-boron particles (NdFeB, 5 µm, Magquench GmbH), enabling the robots with various Young’s modulus *E*_r_. The PDMS to NdFeB mass ratio was 1:4 for all samples. *E*_r_ of different materials were quantified by tensile tests in a mechanical tester (Instron 5942), and the results are shown in Supplementary Fig. 2A. The thoroughly mixed polymer was cast into the negative PDMS mold and cured in the hot oven at 85 °C for 7 hours. Then the sample was put in the vibrating-sample magnetometer (VSM, EZ7, Microsense) with 1.8 T for uniform magnetization. Finally, the robot was demolded and ready for tests. The fabrication details can be seen in Supplementary Fig. 11.

### 6-DoF magnetic actuation system

The hardware components include a 50 mm cubic permanent magnet (N45, IMPLOTEX GmbH), a step motor (NEMA 17) to rotate the magnet, and a linked 7-DoF robotic arm (Panda, Franka Emika GmbH). The communication software was built upon the framework of Robot Operating System (ROS Melodic). There are three major groups of nodes used in the communication frameworks, i.e., the motion command generator node, the step motor controller node, and the arm controller node. Particularly, the motion command can either be from the manual input or the automatically generated via-points of the predefined trajectory. During manual manipulation mode, the command published by the joy is subscribed by the arm controller node (arm joy receiver) and the step motor controller mode (step motor joy receiver). During automatic manipulation mode, the predefined via-points are published by the motion command generator and subscribed by the two controllers (arm command receiver and step motor command receiver). The overall system enables the 6-DoF spatial manipulation around the end-effector, i.e., the cubic magnet. Note that the rotation direction of the robot is the reverse of the magnet due to the specific magnetic field generated by the permanent magnet.

### Force modeling and analyses

There are three major groups of forces contributing to the robot locomotion in phantom B (Supplementary Fig. 12): 1) the magnetic forces applied on the robot along the *y*_r_-axis, *F*_mag_,_yr_, the magnet torque applied on the robot around the *y*_r_-axis, *T*_mag_,_yr_, 2) the fluidic drag, *F*_drag_, and 3) the frictional forces parallel and perpendicular to helix structure, *F*_fric,||_ and *F*_fric,⊥_, respectively.

*F*_mag_,_yr_, and *T*_mag_,_yr_ are modeled using the dipole approximation as^71^

1 $${{{{{{\bf{F}}}}}}}_{{{{{{\rm{mag}}}}}}}\left({{{{{{\bf{p}}}}}}}_{{{{{{\rm{a}}}}}}}^{{{{{{\rm{r}}}}}}},\, {{{{{{\bf{m}}}}}}}_{{{{{{\rm{r}}}}}}}\right)=\left({{{{{{\bf{m}}}}}}}_{{{{{{\rm{r}}}}}}}\cdot \nabla \right){{{{{\bf{B}}}}}}\left({{{{{{\bf{p}}}}}}}_{{{{{{\rm{a}}}}}}}^{{{{{{\rm{r}}}}}}}\right),$$

2 $${T}_{{{{{{\rm{mag}}}}}}}={{{{{{\bf{m}}}}}}}_{{{{{{\rm{r}}}}}}}\times {{{{{\bf{B}}}}}}\left({{{{{{\bf{p}}}}}}}_{{{{{{\rm{a}}}}}}}^{{{{{{\rm{r}}}}}}}\right),$$

where ${{{{{{\bf{m}}}}}}}_{{{{{{\rm{r}}}}}}}$ is the magnetic moment of the robot, and ${{{{{\bf{B}}}}}}\left({{{{{{\bf{p}}}}}}}_{{{{{{\rm{a}}}}}}}^{{{{{{\rm{r}}}}}}}\right)$ is the magnetic flux density generated by the actuation magnet with the magnetic moment of ${{{{{{\bf{m}}}}}}}_{{{{{{\rm{a}}}}}}}$, and ${{{{{{\bf{p}}}}}}}_{{{{{{\rm{a}}}}}}}^{{{{{{\rm{r}}}}}}}$ is the vector pointing from the actuation magnet to the robot. *F*_drag_ is modeled by fluid-structure interaction in COMSOL Multiphysics 5.4. *F*_fric,||_ and *F*_fric,⊥_ are described by the Coulomb model of friction, where the radial force *F*_n_ along with the deformation are modeled by Abaqus 2019 using the measured robot Young’s modulus *E*_r_ (Supplementary Fig. 2a), and the coefficient of friction (CoF) is quantified by friction tests (see “Quantification of the friction properties for the porcine arteries and PDMS phantoms”). The robot as the composite of NdFeB particles and PDMS is modeled as a linear elastic material, enabling the linear relations between *F*_n_ and the radial deformation (Supplementary Fig. 2b). The experimental validation of these forces was individually conducted and explained in Supplementary Note 9.

To realize the shape adaptation, the force relations as shown in Eqs. (3) and (4) need to be satisfied when the robot moves along with the flow (magnetic actuation is on). Similarly, the relations in (4) and (5) need to be satisfied when the robot moves against the flow (magnetic actuation is on). (6) needs to be satisfied when the robot halts at the targeted location such that the self-anchoring can be ensured even the magnetic actuation is off:

3 $${F}_{{{{{{\rm{mag}}}}}},{{{{{\rm{yr}}}}}}}+{F}_{{{{{{\rm{drag}}}}}}}+{F}_{{{{{{\rm{fric}}}}}},\perp }\,\cos (\varphi )\ge {F}_{{{{{{\rm{fric}}}}}},\parallel}\,\sin (\varphi ),$$

4 $${T}_{{{{{{\rm{mag}}}}}},{{{{{\rm{yr}}}}}}}\ge ({F}_{{{{{{\rm{fric}}}}}},\parallel}\,\cos (\varphi )+{F}_{{{{{{\rm{fric}}}}}},\perp }\,\sin (\varphi )){R}_{{{{{{\rm{d}}}}}}},$$

5 $${F}_{{{{{{\rm{mag}}}}}},{{{{{\rm{yr}}}}}}}+{F}_{{{{{{\rm{fric}}}}}},\perp }\,\cos (\varphi )\ge {F}_{{{{{{\rm{drag}}}}}}}+{F}_{{{{{{\rm{fric}}}}}},\parallel}\,\sin (\varphi ),$$

6 $${F}_{{{{{{\rm{drag}}}}}}} \le {F}_{{{{{{\rm{fric}}}}}},\parallel}\,\sin (\varphi )+{F}_{{{{{{\rm{fric}}}}}},\perp }\,\cos (\varphi ),$$

where ${F}_{{{{{{\rm{fric}}}}}},\perp }={\mu }_{\perp }{F}_{{{{{{\rm{n}}}}}}}$, *F*_fric,||_ = *μ*_||_*F*_n_, *φ* is the helix angle, which is 8^°^, ${R}_{{{{{{\rm{d}}}}}}}$ is the robot radius after radial deformation, *μ*_⊥_ and *μ*_||_ are the CoF perpendicular to and parallel to the helix, respectively. *F*_mag_,_yr_ here refers to the maximum achievable value, and *T*_mag_,_yr_ refers to the one when *F*_mag_,_yr_ is acquired.

Concerning the dynamics of the robot during locomotion, it can be modeled as:

7 $${F}_{{{{{{\rm{mag}}}}}},{{{{{\rm{yr}}}}}}}+{F}_{{{{{{\rm{fric}}}}}},\perp }\,\cos (\varphi )-{F}_{{{{{{\rm{fric}}}}}},\parallel }\,\sin (\varphi )\,+a\,{F}_{{{{{{\rm{drag}}}}}}}={m}_{{{{{{\rm{r}}}}}}}{\ddot{w}}_{{{{{{\rm{yr}}}}}}},$$

8 $${T}_{{{{{{\rm{mag}}}}}},{{{{{\rm{yr}}}}}}}-({F}_{{{{{{\rm{fric}}}}}},\parallel }\,\cos (\varphi )+{F}_{{{{{{\rm{fric}}}}}},\perp }\,\sin (\varphi )){R}_{{{{{{\rm{d}}}}}}}={J}_{{{{{{\rm{yr}}}}}}}{\ddot{\theta }}_{{{{{{\rm{yr}}}}}}},$$

where $a$ is a parameter equal to 1 or −1 when the robot moves along and against the flow, respectively. ${w}_{{{{{{\rm{yr}}}}}}}$ is the robot displacement along the ${y}_{{{{{{\rm{r}}}}}}}$-axis, ${m}_{{{{{{\rm{r}}}}}}}$ is the robot mass, ${\theta }_{{{{{{\rm{yr}}}}}}}$ is the rotation angle of the robot around the ${y}_{{{{{{\rm{r}}}}}}}$-axis, and ${J}_{{{{{{\rm{yr}}}}}}}$ is the moment of inertia around the ${y}_{{{{{{\rm{r}}}}}}}$-axis. The dynamic equation was solved by the ODE23 solver in MATLAB R2018a (MathWorks, Inc.). For the detailed force modeling during curved lumens traversing, please refer to Supplementary Notes 2 and 3.

### Preparation of the porcine coronary arteries and thrombus for ex-vivo tests

The porcine hearts and blood were received as an animal side product (registration number: DE 08 111 1008 21). The permit and registration number for the scientific use of animal side products were issued by the office for public order, in particular, the authorities for official food control, consumer protection and veterinary services of the state capital Stuttgart. As requested by the permit, an official holding register of the biomaterial is kept, and the used animal side products are pressure sterilized following the experiment. The coronary arteries for locomotion experiments and aorta for friction tests were cut from the fresh porcine hearts slaughtered within 48 hours and stored under 4 °C (Slaughterhouse Ulm, Germany, and Gourmet Compagnie GmbH, Germany). The cut tissues were firstly cleaned by PBS and then prepared for tests.

There were two sources of thrombi. The first source was directly sampled from the fresh porcine aorta and cut into desired sizes for tests. The second one was manually fabricated from blood, obtained from freshly slaughtered porcine within 48 hours, and stored under 4 °C (Slaughterhouse Ulm, Germany, and Gourmet Compagnie GmbH, Germany). The liquid with the volume ratio of 50 to 1 for blood to calcium chloride aqueous solution (0.5 mol/L) was prepared. After 15 mins at room temperature (23 °C), the thrombi were formulated. For both methods, the thrombi with volumes of around 3.5 mm^3^ were prepared and immersed in PBS for thrombolysis tests.

### SMP-based foldable structure for cargo (drug) loading and releasing

The structure was fabricated with SMP materials using the molding technique. The structures of the desired shape (*t*_4_ step in Fig. 6a) were firstly 3D-printed (IPQ, Nanoscribe GmbH), which were then used to make the negative molds of PDMS (base and crosslinker mass ratio 10:1). The materials used for the synthesis of the SMP were Poly(Bisphenol A-co-epichlorohydrin) glycidyl end-capped (PBGD), Poly(propylene glycol) bis(2-aminopropyl ether) (Jeffamine D230), and Neopentyl glycol diglycidyl ether (NGDE). 1 g PBGD was melted by heating in an oven at 70 °C for 20 min. Next, 360 µL Jeffamine D230 and 300 µL NGDE were added to the melted PBGD and stirred for 5 minutes. The resulting solution was mixed with the iron oxide particles (Sigma-Aldrich) with a mass ratio of 5:4. The composite was cast into the prepared PDMS molds. After curing at 100 °C for 1.5 h in an oven and postcuring at 130 °C for 1 h, the SMP-based structure could be demolded from the PDMS mold.

For the visualization demonstration of cargo releasing (Fig. 6b), the pill made of silk fibroin aqueous solution (15 wt%) doped with fluorescein dye solution (50 wt%) was used (volume ratio 1:3). The compound solution was cast into the PDMS molds with the desired pill shape (indicated in Fig. 6a). After the water was evaporated, solid pills could be acquired and assembled into the slots of the SMP-based structures by using Ecoflex 00-10 (Smooth-On, Inc.) as the connection agent. The overall structure was heated to 50 °C and manually closed, as shown in step *t*_1_ in Fig. 6a. Then the shape was held until the temperature was decreased to room temperature, 23 °C, to fix the shape of SMP. For the final step, the structures with the incorporated pills were assembled to the inner beam of the robot using Ecoflex 00-10. For thrombolysis tests (Fig. 6c, d), the tPA powder (Actilyse^®^, Boehringer Ingelheim International GmbH) was directly loaded into the slots of the SMP-based foldable structures.

### Evaluation of biocompatibility

The murine monocyte-macrophage cell line J774A.1 (ATCC) was used as the model cell line for cell viability studies. J774A.1 were expanded in T-75 cell culture flasks containing Dulbecco’s Modified Eagle’s Medium (DMEM; Gibco) supplemented with 10% fetal bovine serum (Gibco) and 1% penicillin/streptomycin (Gibco). For the experiments, the cells were collected and seeded into 24-well plates containing the respective material sample with the size of 2 mm × 2 mm × 0.1 mm at a concentration of 4 × 10^4^ cells/well in triplicates. The positive control was treated with 20% DMSO (Sigma-Aldrich), and the negative control was left untreated. The cells were incubated at 37 °C in a humidified atmosphere of 5% CO_2_ for up to 72 hours. The SMP samples were incubated for up to 24 hours since the structure was intended for drug release lasting from minutes to several hours. The cell viability after exposure to the different materials was assessed using the Live/Dead Cell Imaging Kit (R37601, Thermo Fisher Scientific). Fluorescence microscopy images were recorded using a Keyence BZ-X800E microscope. Live and dead cells were counted using the integrated hybrid cell count analysis module.

### Evaluation of hemocompatibility

The hemocompatibility of the device was separated into hematotoxicity and thrombogenicity studies. Fresh rat blood was received as an animal side product (registration number: DE 08 111 1008 21) from the Institute for Animal Welfare, Veterinary Service and Laboratory Animal Science, Eberhard Karls University of Tübingen, Germany, and the Institute for Anatomy and Cell Biology, University of Ulm, Germany. Whole blood samples were added to the wells containing the material samples with the sizes of 2 mm × 2 mm × 0.1 mm, and the plates were agitated for 15 min, 1200 revolutions per minute (rpm) at room temperature. After the incubation, the samples were used for hematoxylin and eosin staining and luciferase assay. Three independent experiments were performed using fresh rat blood samples for each test^72^. In the hematotoxicity study, hematoxylin and eosin staining (ab245880, Abcam) were conducted. After the staining, the slides with thick blood smear samples were embedded with the xylene-based mounting medium. Light microscopy images were collected from each sample in different experimental groups. In the thrombogenicity study, platelet activation was measured using the luminescence method in a plate reader (BioTek Synergy HTX, Agilent). The ATP release from activated platelets was measured using a luciferin-luciferase reaction for the platelet activation test^73^. This test used a ready-to-use Luciferin-luciferase ATP kit (CellTiterGlo Luminescent Cell Viability Assay, Promega). 100 µL ATP mix was added into the well containing the previously agitated samples for 15 min, 1200 rpm at room temperature before measuring platelet activation using luminescence. The whole blood sample with collagen type I (100 µg/ml, ibidi) was used as a positive control, and the whole blood samples incubated with Parylene C-coated glass slides were used as a negative control.

### Collection of visual data

For the quantitative experiments evaluating the locomotion and functions of the robot, the data was collected using a commercial camera (Blackfly S USB3, Teledyne FLIR LLC) and the compatible software (SpinView 2.4.0). The ultrasound-based robot inspection in the porcine coronary artery-based ex-vivo experiments was conducted by the B-mode of a commercial medical ultrasound machine (Vevo 3100, FUJIFILM Visualsonics, Inc). The imaging stream was imported to the PC using a video grabber (DVI2USB 3.0, Epiphan Systems Inc) and a public ROS-compatible driver for video streaming^74^. The X-ray inspection was conducted using a commercial system (XPERT^®^ 80, Cabinet X-ray System, KUBTEC^®^ Scientific) and its compatible operating software (KubtecNC 3.0.0.0).

### Statistical analysis

All quantitative values from the experiments were presented as means plus/minus the standard deviation. Each quantitative investigation was conducted on at least two robot samples. The two-sample *t*-test and one-way ANOVA test were used for the statistical analysis. We set the statistical significance at 95% confidence level (*P* < 0.05).

### Reporting summary

Further information on research design is available in the Nature Research Reporting Summary linked to this article.

## Supplementary information

Supplementary Information

Description of Additional Supplementary Files

Supplementary Movie 1

Supplementary Movie 2

Supplementary Movie 3

Supplementary Movie 4

Supplementary Movie 5

Supplementary Movie 6

Supplementary Movie 7

Supplementary Movie 8

Supplementary Movie 9

Supplementary Movie 10

Reporting Summary

## Data Availability

The data evaluating the findings in this study are included in the article and its supplementary information. All data are available from the corresponding author upon reasonable request.

## Code availability

All relevant code is available upon request from the corresponding author.
