# Supplementary material for: Adaptive wireless millirobotic locomotion into distal vasculature
Source: Nat Commun. 2022 Aug 1;13:4465. doi: 10.1038/s41467-022-32059-9 (PMC9343456; doi:10.1038/s41467-022-32059-9)
Supplement: Supplementary file 1 — Supplementary Information [file 41467_2022_32059_MOESM1_ESM.pdf]

Supplementary Materials for

Adaptive Wireless Millirobotic Locomotion Into Distal Vasculature

Tianlu Wang[1,2], Halim Ugurlu[1,3,4], Yingbo Yan[1], Mingtong Li[1], Meng Li[1], Anna-Maria Wild[1], Erdost Yildiz[1], Martina Schneider[1], Devin Sheehan[1], Wenqi Hu[1†], Metin Sitti[1,2,5†]

[1] Physical Intelligence Department, Max Planck Institute for Intelligent Systems, 70569 Stuttgart, Germany

[2] Department of Information Technology and Electrical Engineering, ETH Zurich, 8092 Zurich, Switzerland

[3] Clinic for Neuroradiology, Klinikum Stuttgart, 70174 Stuttgart, Germany

[4] Department of Biophysics, Aydın Adnan Menderes University, Graduate School of Health Sciences, 09010 Aydın, Turkey

[5] School of Medicine and College of Engineering, Koç University, 34450 Istanbul, Turkey

[†] Correspondence to: wenqi@is.mpg.de, sitti@is.mpg.de

Supplementary Figures

a

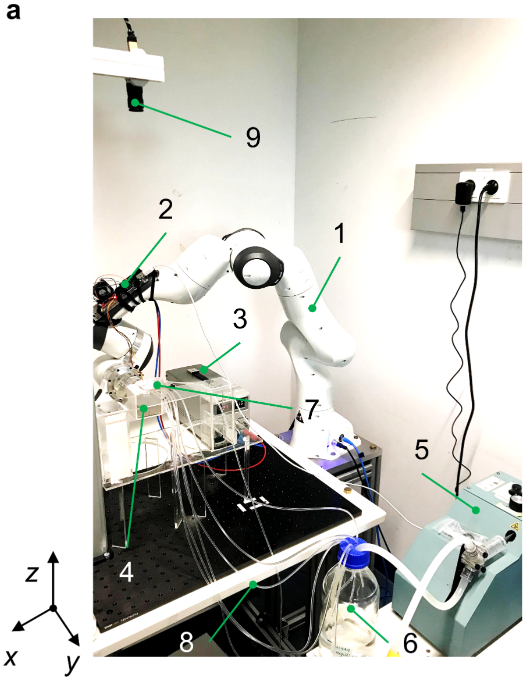

b

1	Robotic arm
2	Step motor controller & driver
3	Power source for step motor
4	Permanent magnet
5	Pulsatile flow pump
6	Blood analog
7	PDMS phantom
8	Connecting tubings
9	Camera

Supplementary Figure 1. Experiment setup with individual components.

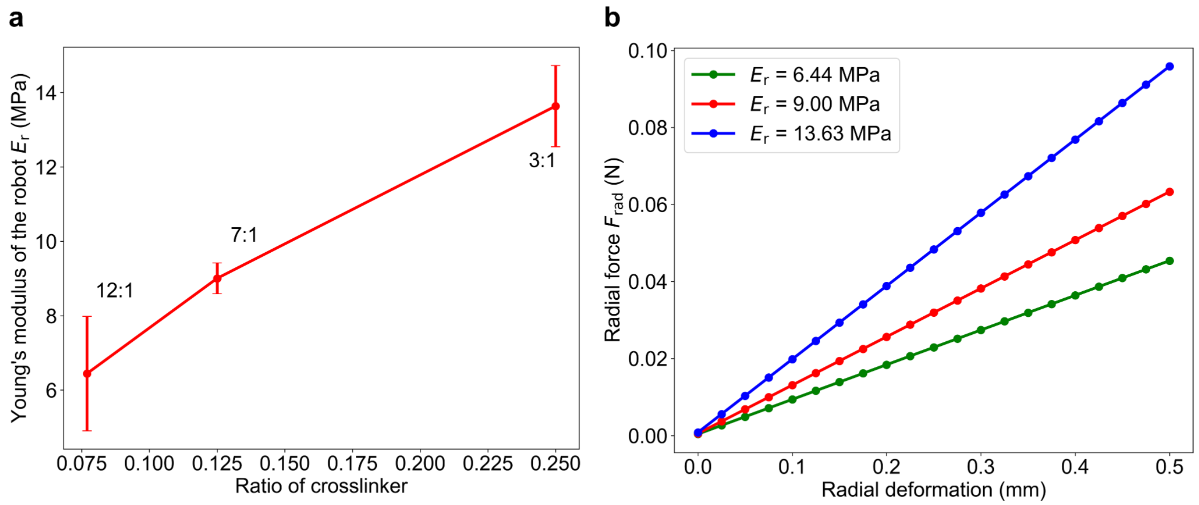

Supplementary Figure 2. Quantification of radial forces F_n during deformation for robots with different Young's modulus E_r. a Characterization of E_r for magnetic composite with different mass ratios of crosslinker. The ratios of the base to crosslinker are indicated in the figure. For all cases, the mass ratio between PDMS (base and crosslinker) and the NdFeB particles is 1:4. The data are presented as mean values ± standard deviation for $n = 3$. **b** Modeling results on the F_n along with radial deformation for robots with various E_r.

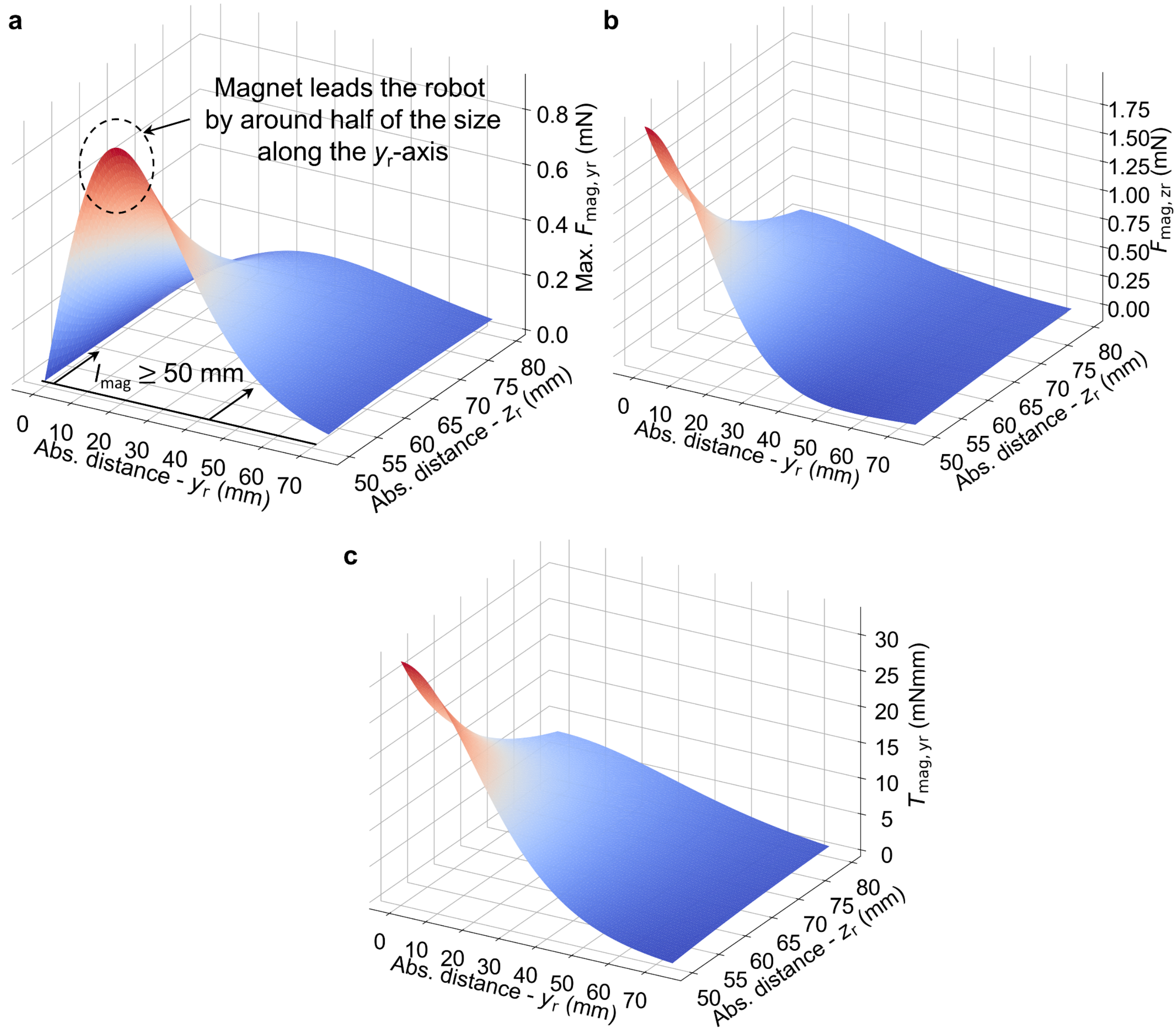

Supplementary Figure 3. Quantification of magnetic forces and torques. **a** Variation of the maximum magnetic force along the y_r-axis $F_{mag,yr}$ for different distances between the robot and the magnet along with the y_r-and z_r-axes. Maximum $F_{mag,yr}$ is achieved when the magnet leads the robot by around half of the size of the magnet, i.e., 25 mm. **b** Variation of magnetic force along the z_r-axis $F_{mag,zr}$. **c** Variation of magnetic torque around the y_r-axis $T_{mag,yr}$.

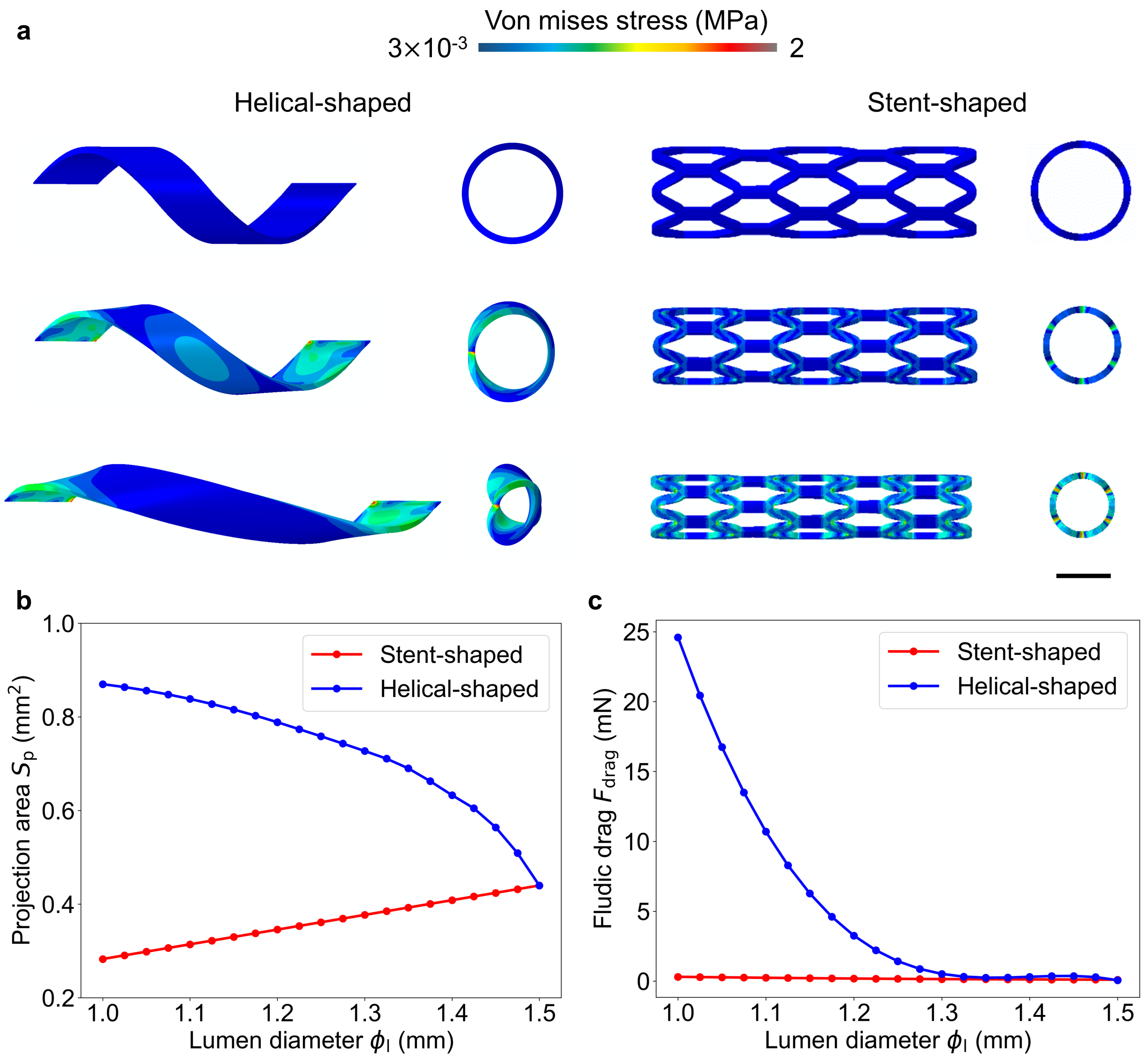

Supplementary Figure 4. Comparison of the flow drag F_{drag} between the stent-shaped design and helical-shaped design. **a** Simulation of the robot's adaptation into the lumen with smaller Φ_l (top view and side view). Both designs have the same body length and thickness. Therefore, the projection area S_p is the same when the robot is not deformed. When the helical-shaped robot is adaptive into the lumen with smaller Φ_l, the structure is stretched due to boundary constraint, and S_p is increased. In contrast, for the stent-shaped robot, due to the radial deformability, S_p is decreased. Scale bar: 1mm. **b** Variation of the S_p along with Φ_l. **c** Variation of F_{drag} along with Φ_l. The increasing of S_p notably increases F_{drag} for the helical-shaped design, which further justifies the advantage of stent-shaped structure in flow withstanding.

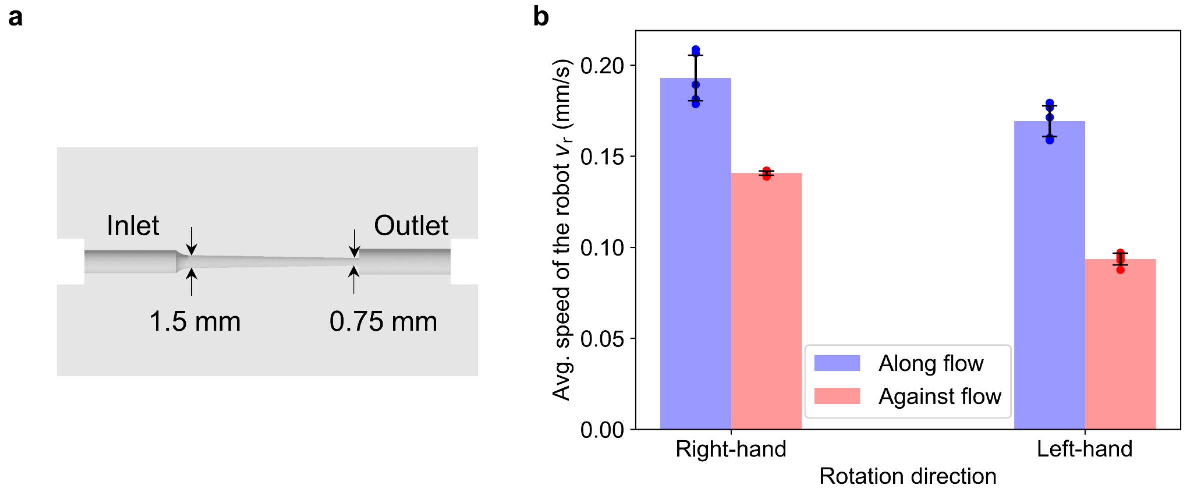

Supplementary Figure 5. Role of the helix on locomotion. The right-handed rotation and the right-handed helix enabled the enhanced speed, especially when moving from the narrower lumen to the wider lumen in phantom A. $P = 0.01$ and 3.13×10^{-9} for along flow and against flow cases, respectively. The data are presented as mean values ± standard deviation for $n = 5$. $v_{mag} = 0.5$ mm/s, $f_{mag} = 0.5$ Hz, and $l_{mag} = 55$ mm.

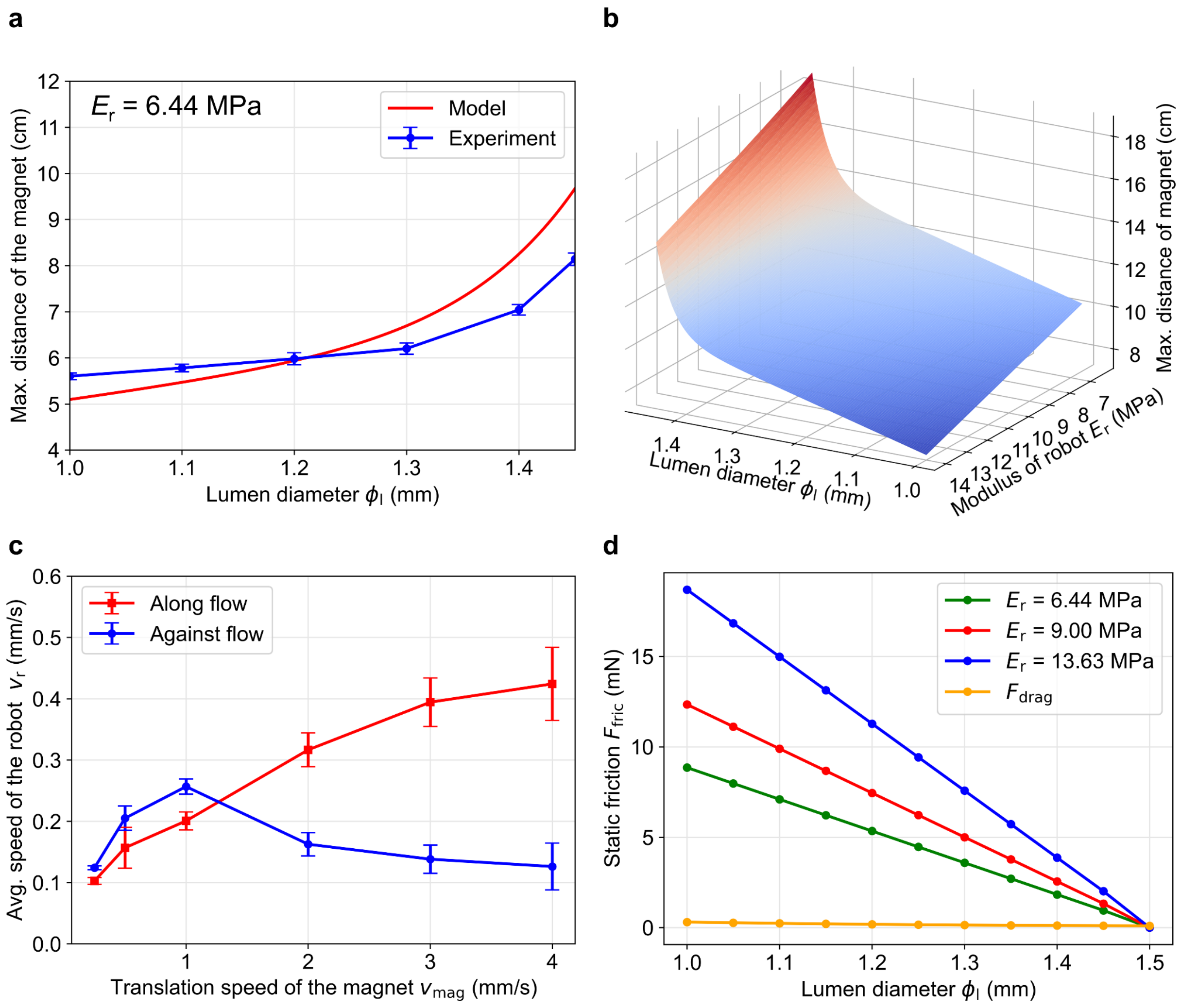

Supplementary Figure 6. Details of the radial shape adaptation and self-anchoring. **a** Experimental and modeling results on the maximum allowed distance of the magnet l_{mag_max} along with Φ_l for the robots with E_r = 6.44 MPa. **b** Effect of Φ_l and E_r on l_{mag_max} for porcine arteries. Due to the decreasing friction in arteries, the requirement on l_{mag_max} can be much more relaxed. **c** Average speed of the robot v_r for various translation speeds of the magnet v_{mag} (f_{mag} = 0.5 Hz, l_{mag} = 55 mm). At v_{mag} = 0.5 mm/s, where the robot can properly follow the magnet, the robot achieved the average speed v_r at around 0.18 mm/s. Note that for higher v_{mag}, v_r can be increased, but the lagging is also more apparent. **d** Self-anchoring of the robot utilizing friction when the external magnetic field is off for porcine arteries. In **a** and **c**, the data are presented as mean values ± standard deviation for n = 5.

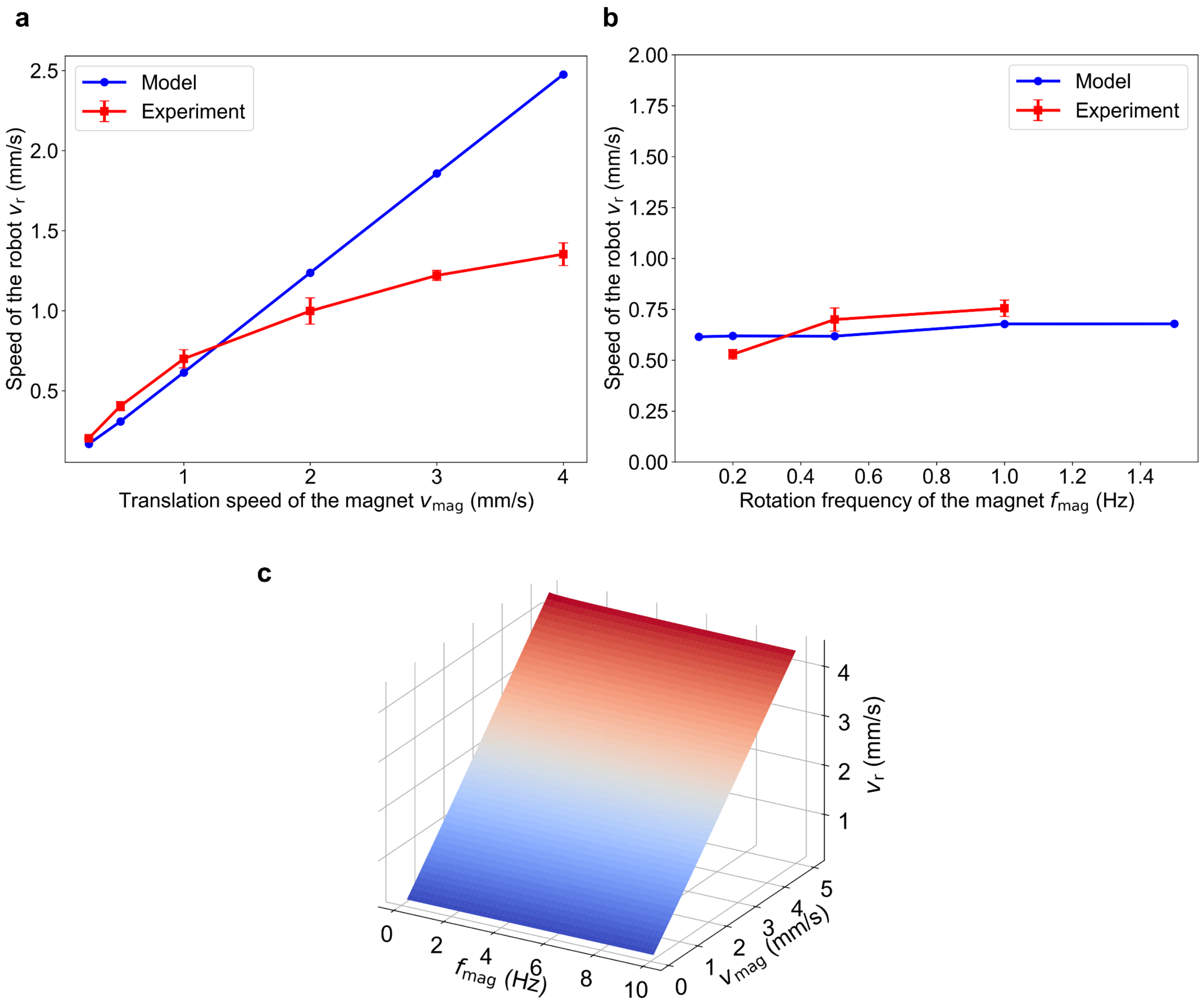

Supplementary Figure 7. Modeling and experimental results on speed of the robot in phantom B without flow. Motion commands were generated under the automatic mode to rule out the randomness in manual operation. l_{mag} was set to be 55 mm. **a** Robot speed v_r along with the translation speed of the magnet v_{mag}. The rotation frequency f_{mag} was set to be 0.5 Hz. **b** Robot speed along with f_{mag}. v_{mag} was set to be 1 mm/s. **c** Relation between robot speed v_r and f_{mag}, v_{mag}. The influence of v_{mag} is much more significant than f_{mag}. In **a** and **b**, the data are presented as mean values ± standard deviation for $n = 3$.

a

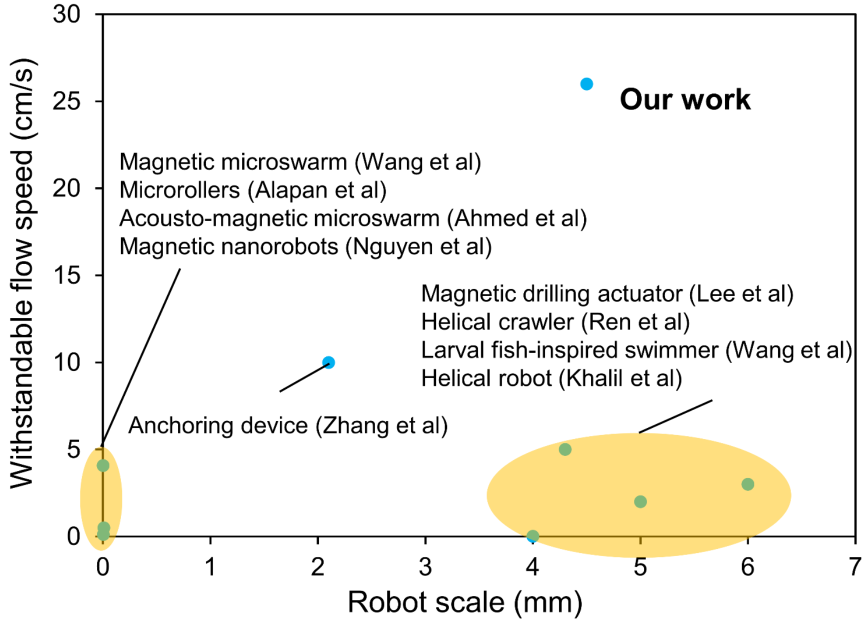

b

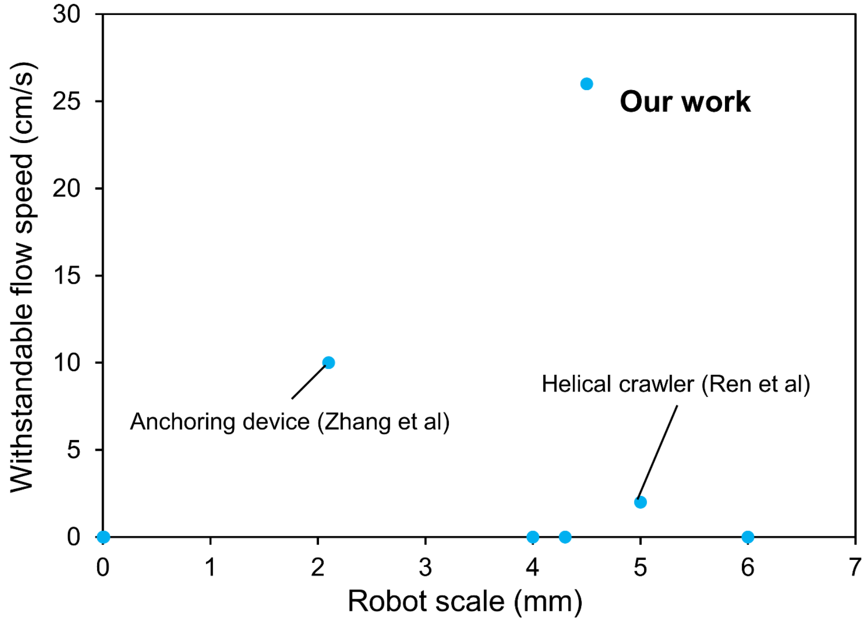

Supplementary Figure 8. Comparison of flow withstanding among available micro/millirobots for endovascular applications. **a** The withstandable flow speed when the control signals are on. **b** The withstandable flow speed when the control signals are off.

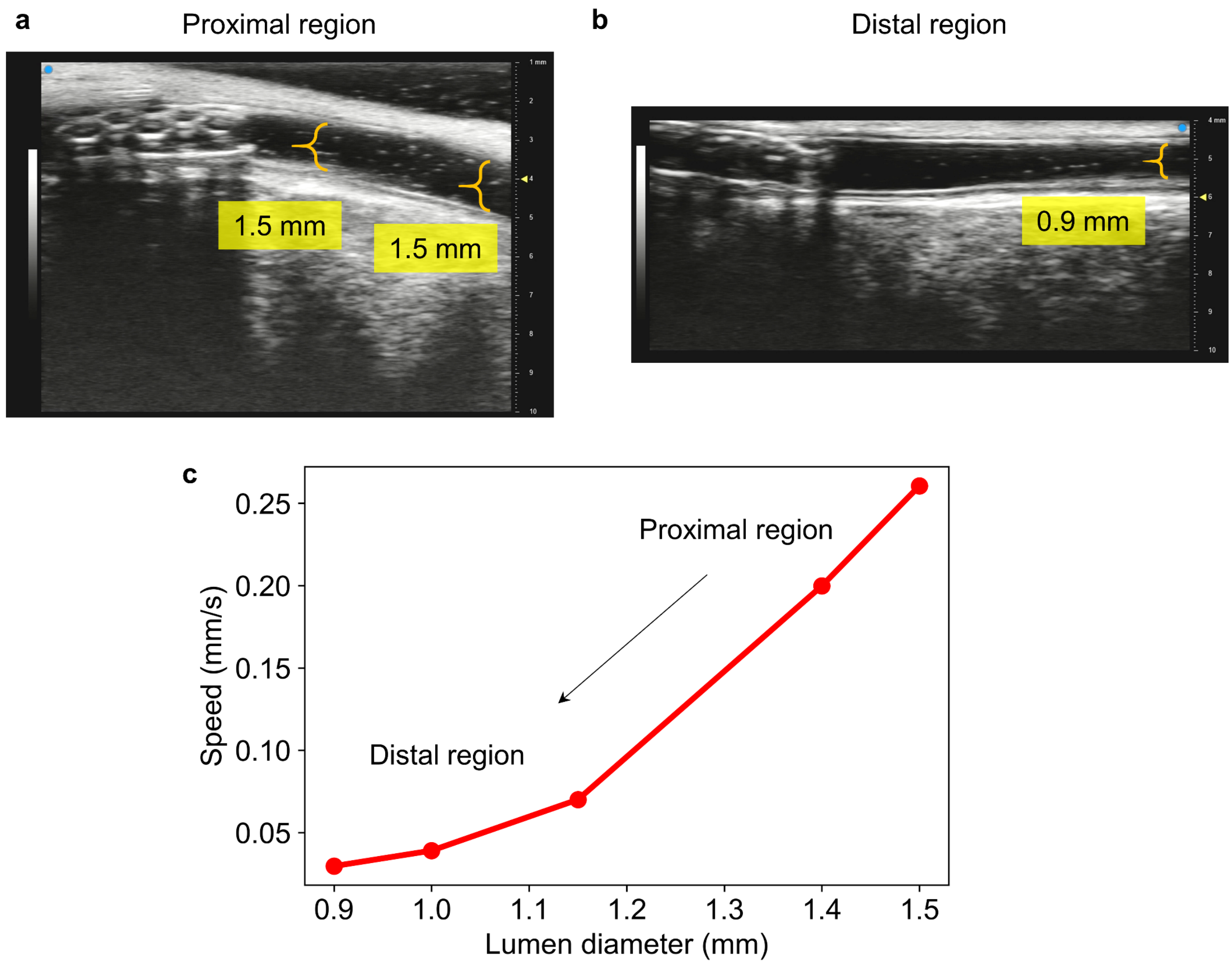

Supplementary Figure 9. Experimental details of the *ex vivo* tests in porcine arteries. **a** and **b** Lumen diameters at the proximal and distal regions of the porcine coronary artery, respectively. **c** Robot speed along with the lumen diameters (estimated from the along-flow locomotion).

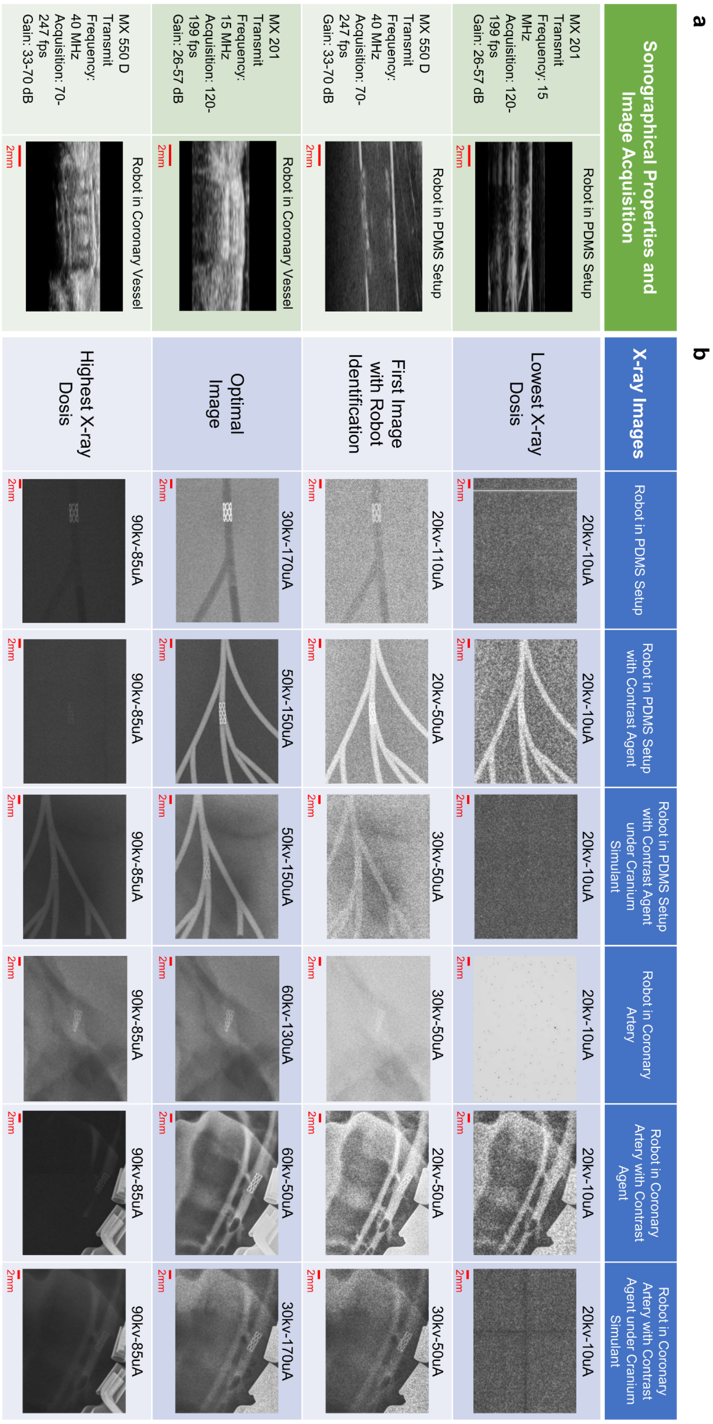

Supplementary Figure 10. Investigation of the robot detection using two medical imaging modalities. **a** Imaging parameters for detection by ultrasound. **b** Imaging parameters for detection by X-ray**.**

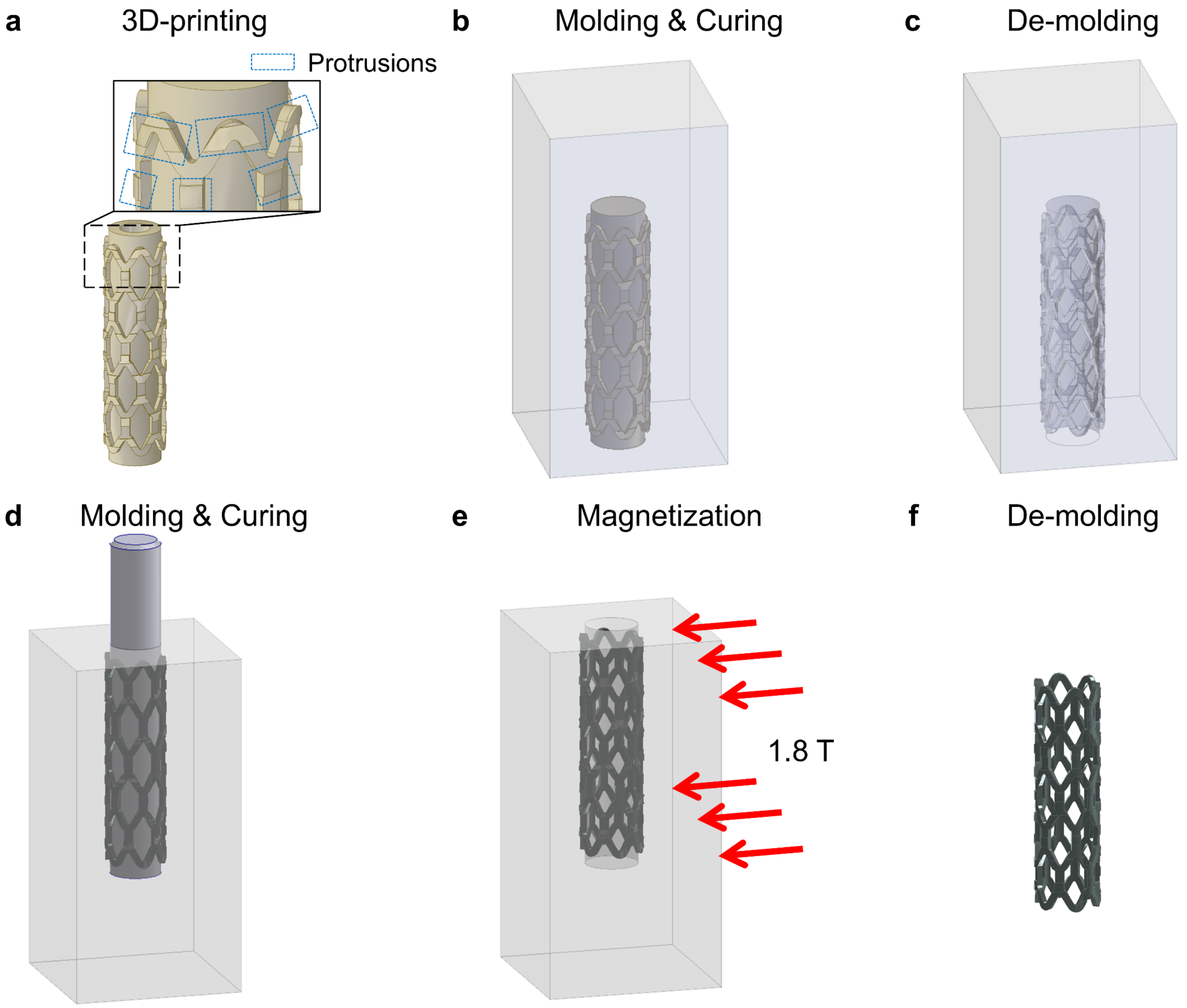

Supplementary Figure 11. Fabrication details of the stent-shaped magnetic soft robot. **a** 3D-printing of the positive mold. The helix structure as the protrusions are also printed on the stent-shaped body. **b** Fabrication of the PDMS-based negative mold. **c** De-molding to get the negative mold. **d** Molding and curing the robot by PDMS and NdFeB composite. A stainless pin is inserted into the mold center to obtain the desired shape of the robot. **e** Magnetization of the robot under the 1.8 T uniform magnetic field. **f** De-molding the robot from the PDMS mold.

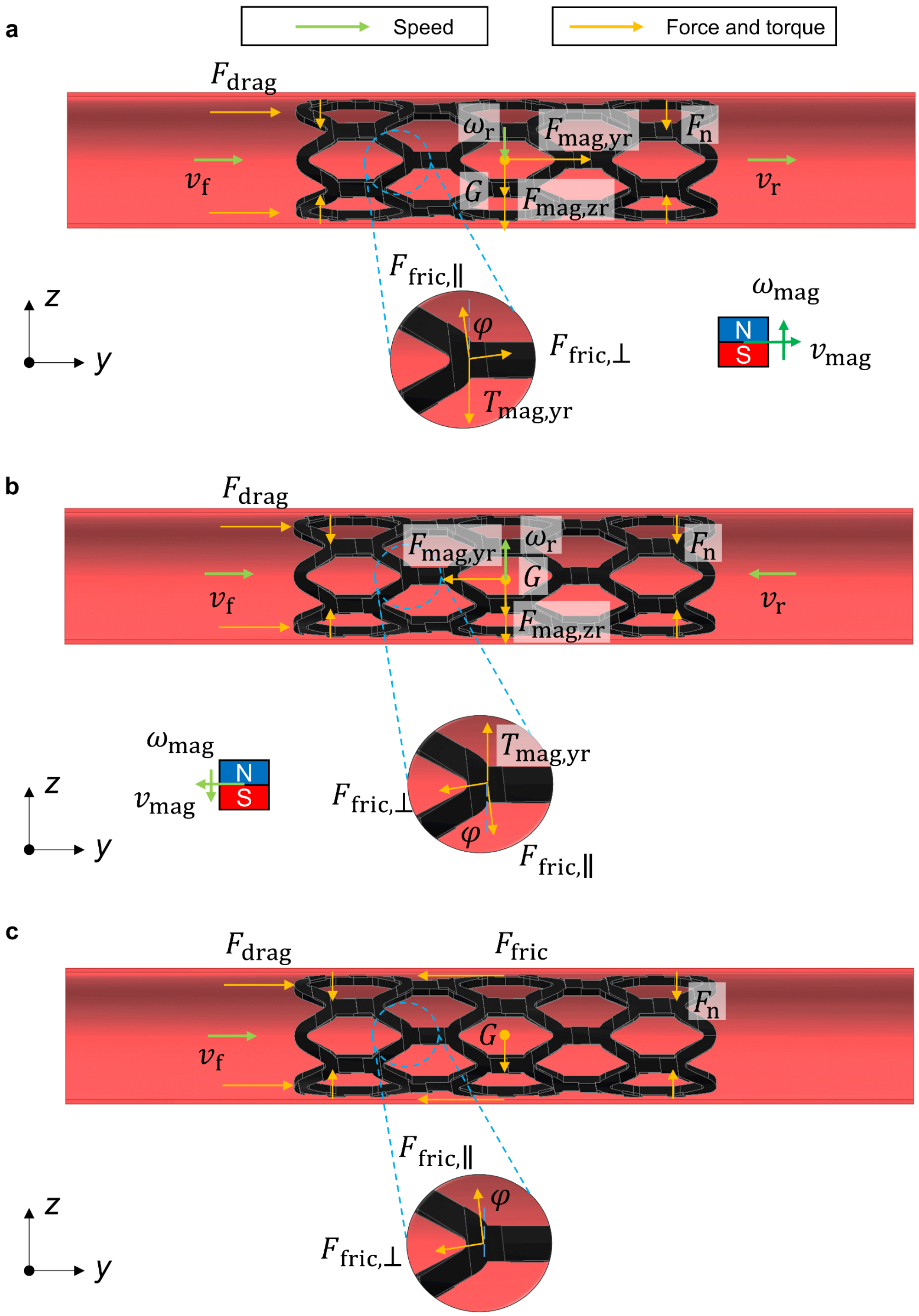

Supplementary Figure 12. Force diagrams during robot locomotion in phantom B. **a** Along with flow locomotion (the magnetic field is applied). **b** Against flow locomotion (the magnetic field is applied). **c** Halting at the targeted location (no magnetic field is applied).

Supplementary Tables

Supplementary Table 1. Limitations of the current minimally invasive endovascular operations for the diseases in distal cortical artery segments

Disease	Occurrence rate	Symptoms	Current therapy	Limitation of the current therapy	Requirement on the new device (our contribution)
Acute ischemic stroke	16 % for ACA/M3/M4[1]; 24%-40% for distal, medium vessel occlusions[2]; 1 % for M4[3] (possibly underestimate actual occurrence due to sensitivity of medical imaging[2])	Partial aphasia, fractionated hemiparesis or hemianesthesia, as well as partial or complete hemivisual field defects[2,4]	Intravenous thrombolysis	1. Systematic rather than targeted application and fail to recanalize 50% to 67% of distal occlusions[2]<br>2. Repeat thrombolysis within three months raises the concern for the risk of hemorrhage[5,6]	Controlled local drug release to lower the systematic dosage while maintaining the efficacy
			Intra-arterial thrombectomy (retrievers and aspiration devices)	Longer, more tortuous access routes, thinner arterial walls of the M4 segment, and possible extensive atherosclerosis 1. limit the catheter navigation; 2. constrain the physical forces deliverable by a retrieval device separated from its manipulable end by multiple turns, and 3. potentially increase the risk of dissection, perforation, and vasospasm by using catheters[2,7]	1. Controlled retrievable navigation among the targeted distal regions<br>2. Safe mechanical forces applied to the arterial walls

			Intra-arterial thrombolysis	1. Longer, more tortuous access routes, thinner arterial walls of the M4 segment, and possible extensive atherosclerosis limit the catheter navigation[8]<br>2. Risks of intracranial hemorrhage[5,6,9,10]	1. Controlled retrievable navigation among the targeted distal regions<br>2. Controlled local drug release to further minimize dosage
Aneurysm	1% for M4[11]	Sudden severe headache, nausea, vomiting, and a slight hemiparesis (after the rupture of their aneurysmal sac)[11]	Intra-arterial coil embolization	Require placing multiple catheters in the parent artery, which may have a higher risk of thromboembolic events in small vessels[12]	Simplification of the therapeutic agent placement locally
			Intra-arterial flow diversion	1. Challenging due to small vessel caliber, vessel fragility, and eloquence of branch arteries[13]<br>2. Potential misplacement[14,15] or migration[16,17]	1. Controlled navigation among the targeted distal regions<br>2. Adjustability of the stent after full deployment
Cerebral arterial-venous malformation	6 out of 7 at cortical region[18]	Intracranial hemorrhage, seizures, headaches, and long-term disability[19]	Intra-arterial embolization	Unintended vessel embolization, incomplete embolization, intracranial hemorrhage, and normal perfusion pressure breakthrough,	1. Controlled retrievable navigation among the targeted distal regions

				leading to edema or hemorrhage[19]	2. Controlled local drug release
Dural arteriovenous fistulas	On the cortical region	Headache, seizures, speech or language issues, etc[20]	Transarterial or transvenous embolization[21]	Challenging to get the closest proximity to the fistula point[21]	1. Controlled retrievable navigation among the targeted distal regions<br>2. Controlled local drug release
Brain tumor	Most malignant primary brain tumors occur in the cerebral cortex, with the highest percentage 26% developing in the frontal lobe[22]	Headaches, seizures, difficulty in thinking, etc[22,23]	Intra-arterial therapeutic agents delivery	1. Complications related to catheter placement and vascular access[24]<br>2. Mechanical damage to the vessel wall[25]	1. Controlled retrievable navigation among the targeted distal regions<br>2. Controlled local drug release<br>3. Safe mechanical forces applied to the arterial walls

Supplementary Table 2. Characterization of the coefficient of friction (CoF)

Test samples	Type	CoF	Value
Porcine artery	Static	$\mu_{sa,\perp}$	0.18 ± 0.04
		$\mu_{sa,\parallel}$	0.12 ± 0.01
	Kinetic	$\mu_{ka,\perp}$	0.08 ± 0.01
		$\mu_{ka,\parallel}$	0.07 ± 0.01
PDMS	Static	$\mu_{sp,\perp}$	0.47 ± 0.13
		$\mu_{sp,\parallel}$	0.38 ± 0.08
	Kinetic	$\mu_{kp,\perp}$	0.13 ± 0.03
		$\mu_{kp,\parallel}$	0.08 ± 0.04

Supplementary Table 3. Design parameters of the robot

Parameters	Value
Strut spacing h (mm)	0.4
Radius of curvature at the crown junction ρ (mm)	0.3
Axial amplitude of each segment f (mm)	0.5
Overall length l_r (mm)	5
Initial diameter Φ_r (mm)	1.5
Wall thickness t_r (mm)	0.1
Coated helix pitch p_r (mm)	0.5
Helix revolution number	10
Helical angle φ (deg)	8
Mass m_r (kg)	$2.68 \times 10^{-6} \pm 1.37 \times 10^{-7}$
Volume V_r (m^3)	0.77×10^{-9}
Young's modulus E_r (MPa)	6.44 ± 1.1, 9.00 ± 0.4, and 13.63 ± 1.5
Magnetic moment m_r ($A \cdot m^2$)	$1.27 \times 10^{-4} \pm 9.43 \times 10^{-6}$

Supplementary Table 4. Design of the PDMS-based phantoms

Phantom label	Design	Important parameters
A	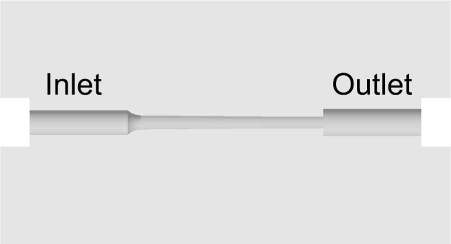	$\Phi_l = 0.75 – 1.5$ mm
B	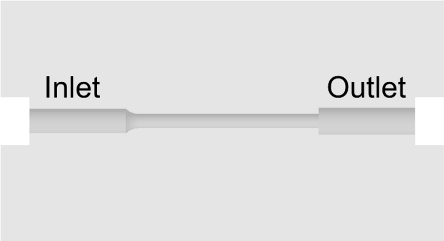	$\Phi_l = 1.45$ mm
C – E	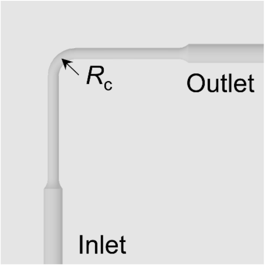	$\Phi_l = 1.45$ mm; $R_c = 3 –$ 7 mm with 2 mm as the step; $\gamma_i = 90^{\circ}$
F – I	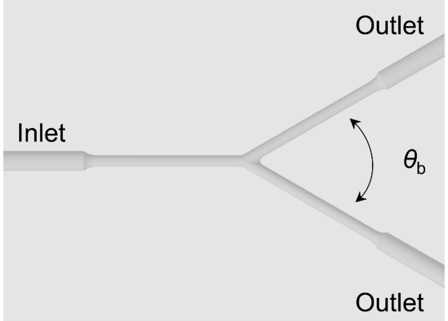	$\Phi_l = 1.45$ mm; $\theta_b = 30^{\circ}$ – 120° with 30° as the step

J	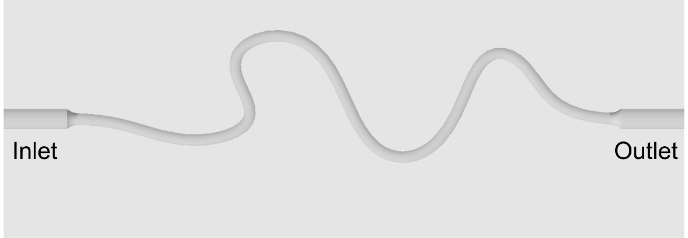	$\Phi_l = 1.45$ mm; $R_c = 2.5 – 5$ mm
K	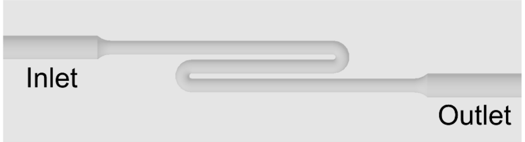	$\Phi_l = 1.45$ mm; $R_c = 1$ mm; $\gamma_i = 180^{\circ}$
L	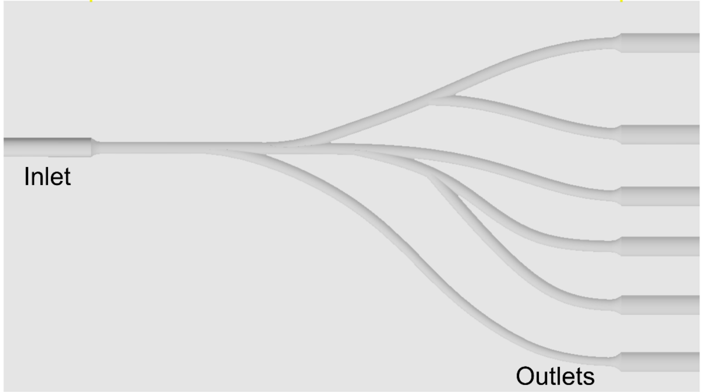	$\Phi_l = 1 – 1.45$ mm
M	The spatial version of L, where L is fixed on the cerebral hemisphere and brain stimulant	$\Phi_l = 1 – 1.45$ mm
N	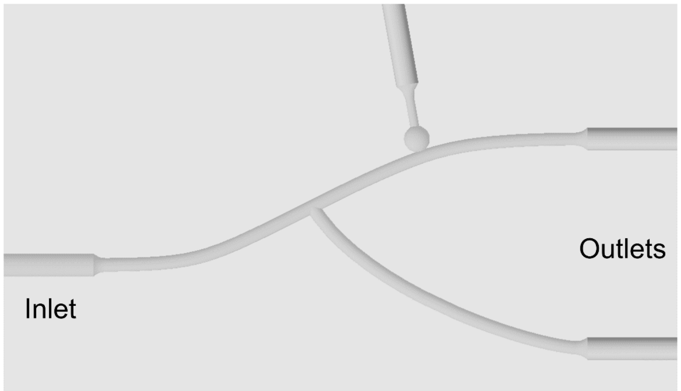	$\Phi_l = 1 – 1.45$ mm; aneurysm sac diameter is 2 mm; neck area is 0.44 mm^2

O	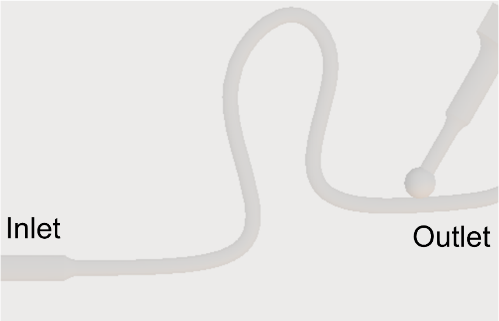	Φ_l = 1.45 mm; $R_c \geq 3$ mm; $\gamma_i \leq 120^{\circ}$
P	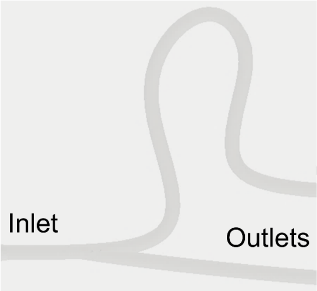	Variant of Phantom O with two branches
Q – S	Straight lumen phantoms based on MCA M1 – M3	Φ_l = 2.4 – 2.7 mm; 2 – 2.4 mm; 1.5 – 2 mm
T	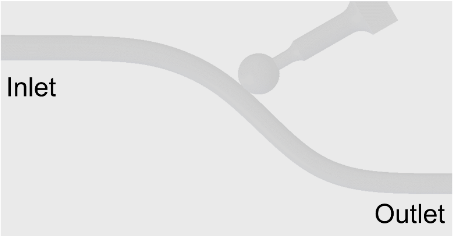	Φ_l = 2.4 mm – 2.7 mm; R_c = 6 mm
U	Straight lumen phantom with even smaller Φ_l	Φ_l = 0.2 – 1.5 mm;

Supplementary Table 5. Physical properties of the blood analog and comparison with blood

Parameters	Blood analog	Blood (normal artery)
Temperature T_a and T_b (°C)	23	37
Density ρ_a and ρ_b (kg·m^{-3})	1.11×10^3	1.06×10^3 [57]
Dynamic viscosity μ_a and μ_b (N·s·m^{-2})	4.4×10^{-3}	4.4×10^{-3} [48, 49]

Supplementary Table 6. Nomenclature of the variables

Variables	Explanation
f_{mag} (Hz)	Rotation frequency of the magnet
v_{mag} (mm/s)	Translation speed of the magnet
Φ_l (mm)	Lumen diameter
Φ_r (mm)	Initial outer diameter of the robot
l_r (mm)	Initial length of the robot
R_d (mm)	Robot radius during deformation, i.e., $R_d = 0.5\Phi_l$
l_s (mm)	Cortex-to-scalp distance
l_{mag} (mm)	Distance between the magnet and the robot along the z_r–axis
l_{mag_max} (mm)	Maximum allowed l_{mag} for adapting to various Φ_l
S_p (mm^2)	Projection area of the robot to the x_r-z_r plane
E_r (MPa)	Young's modulus of the robot material
F_{drag} (mN)	Fluidic drag
λ_h (%)	Completeness of helix
F_n (mN)	Radial force
F_{fric} (mN)	Frictions with the lumen wall
I_r (m^4)	Second moment of area for the cross-section along the body-attached x_r-z_r plane

I_b (m^4)	Second moment of area for the cross-section of a single beam on the composing diamnond-shaped cell
l_b	Length of the single beam on the composing diamnond-shaped cell
Δl_c	Change in distance of two facing beams of the cell under deformation
κ_c (m^{-1})	Curvature of the phantom route
R_c (m)	Radius of curvature of the phantom route
θ_b (deg)	Bifurcation angle of the phantom route
γ_i	Inclination angle of the phantom route
T_{min} (Nm)	Minimum required torque to bend the robot into the curved lumens
F_{mag} and T_{mag} (mN and Nm)	Magnetic forces and torques
F_{react} and T_{react} (mN and Nm)	Reactions force and the induced torque from the lumen
$F_{fric,\parallel}$ and $F_{fric,\perp}$ (mN)	Frictions along and perpendicular to helix
$\mathbf{B}(\mathbf{p}_a^r)$	Magnetic flux density generated by the actuation magnet with the magnetic moment of $\mathbf{m}_a$, and $\mathbf{p}_a^r$ is the vector pointing from the actuation magnet to the robot
t_p (µm)	Thickness of the Parylene-C coating layer

Supplementary Notes

Supplementary Note 1. Asymmetric friction-induced rolling

The permanent magnet-based robot manipulation leads to the asymmetric friction distributions on the robot along the $x_{\mathrm{r}} - z_{\mathrm{r}}$ plane, which causes it to roll and induces the reaction force F_{react} from the lumen. This behavior can be used for curved lumen traversing. As indicated in Supplementary Figure 13a, the direction of the induced F_{react} is decided by the magnet configuration, i.e., the rotation direction and the location relative to the robot (above or beneath).

To illustrate the mechanism, we use the example that the magnet is placed beneath the robot and rotates it around the positive side of the y_{r}-axis (1 in Supplementary Figure 13a). We divide the robot into the upper and lower parts to explain the details of force relations, where the distributed forces are assumed to be centered for ease of illustration (Supplementary Figure 13b). The equilibrium along the z_{r}-axis for the upper part can be represented as

$$F_{\mathrm{n,up}} = F_{\mathrm{in}} - 0.5(m_{\mathrm{r}}g + F_{\mathrm{mag,zr}}), \tag{1}$$

where $F_{\mathrm{n,up}}$ is the normal force applied to the robot from the lumen wall, F_{in} is the inner force from the other part. In contrast, the force equilibrium for the lower part can be represented as

$$F_{\mathrm{n,down}} = F_{\mathrm{in}} + 0.5(m_{\mathrm{r}}g + F_{\mathrm{mag,zr}}). \tag{2}$$

Thus, the corresponding kinetic frictions along the $x_{\mathrm{r}} - z_{\mathrm{r}}$ plane can be represented as

$$F_{\mathrm{n,down}} = F_{\mathrm{in}} + 0.5(m_{\mathrm{r}}g + F_{\mathrm{mag,zr}}), \tag{3}$$

$$F_{\mathrm{fric,down}} = \mu_{\parallel} F_{\mathrm{n,down}} \cos(\varphi) + \mu_{\perp} F_{\mathrm{n,down}} \sin(\varphi), \tag{4}$$

respectively. Thus, it is clear that $F_{\mathrm{fric,down}} > F_{\mathrm{fric,up}}$, i.e., the friction on the lower interface (pointing to the positive x_{r}-axis) is greater than the one on the upper one (pointing to the negative x_{r}-axis) (1 in Supplementary Figure 13a). Thus, the robot tends to roll to the positive side of the x_{r}-axis such that the reaction force from the lumen wall F_{react} balances the frictions along the x_{r}-axis. The quantified $F_{\mathrm{fric,up}}$ and $F_{\mathrm{fric,down}}$ are shown in Supplementary Figure 13c (based on the PDMS phantoms, but the theories also apply to the arteries). The directions of the induced F_{react} for the other three configurations can be extrapolated (2 – 4 in Supplementary Figure 13a).

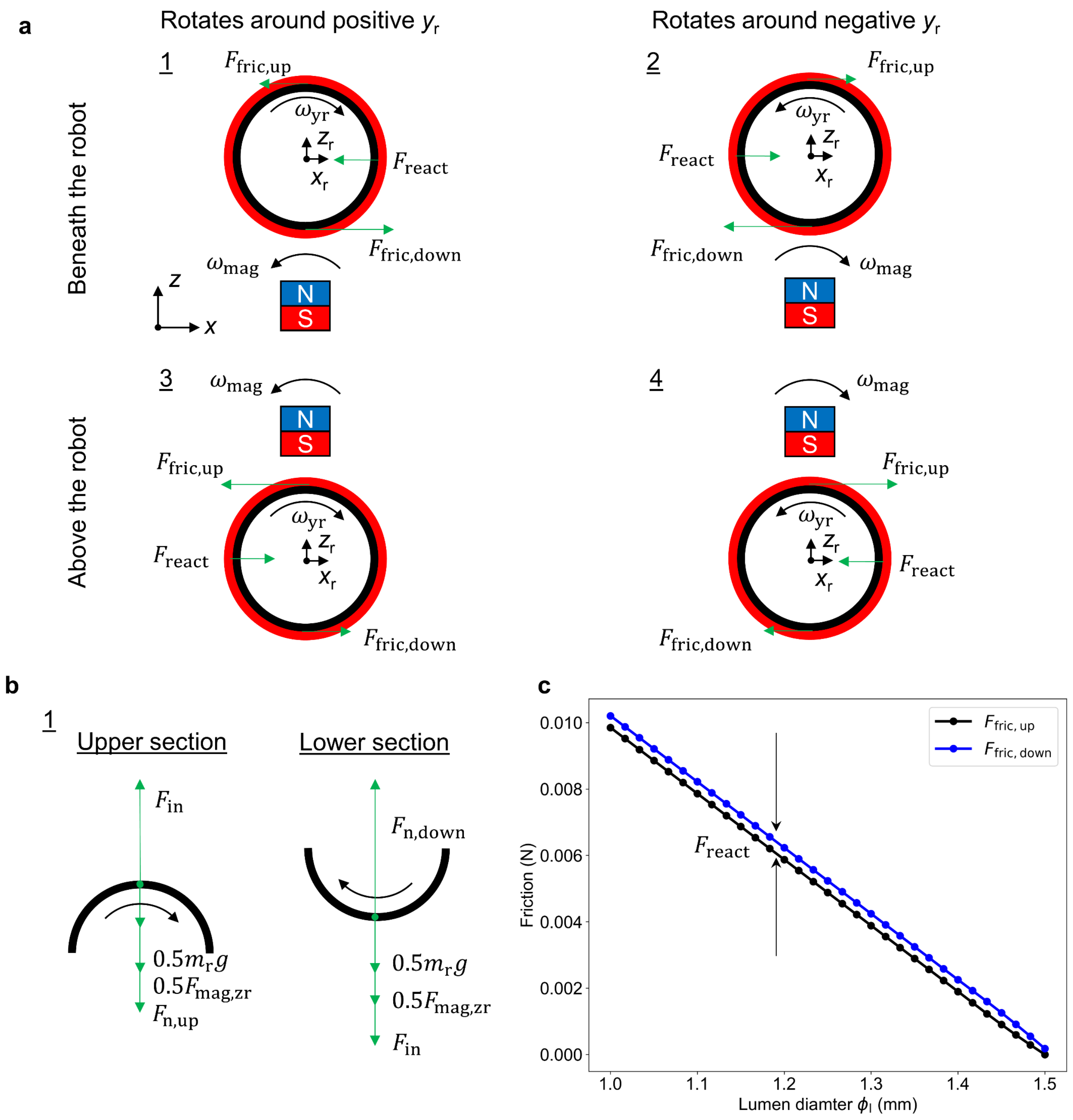

Supplementary Figure 13. Asymmetric friction-induced rolling tendency. **a** Overall force diagrams for the equilibrium along the x-axis in four magnet configurations. The asymmetrical $F_{fric,up}$ and $F_{fric,down}$ enable the rolling tendency to different sides of the x_r-axis, inducing the corresponding F_{react}. **b** Detailed analyses of forces for the upper and lower sections of the robot in configuration 1 of **a**. **c** Quantification on $F_{fric,up}$ and $F_{fric,down}$ for configuration 1.

Supplementary Note 2. Quantification of torques enabling the traversing among curved routes

Among the curved lumens (curved routes and bifurcations), three requirements need to be satisfied simultaneously to enable the traversing, i.e., 1) the maximum leading forces are greater than the maximum resistance forces (along the robot body-attached y_{r}-axis), 2) the maximum actuation torques around the y_{r}-axis are greater than the maximum resistance torques, 3) and the applied bending torques on the robot around the z_{r}-axis are greater than the minimum required torque to bend the robot into the desired curved routes. We regard the curved routes as the consecutive sequence of numerous straight lumen with infinitesimal length. Thus, the analyses of 1) and 2) can be referred directly from the straight lumen case, as explained in the main text. The following details will be about requirement 3).

The torques enabling the traversing of curved routes are composed of magnetic components, i.e., a) magnetic pulling force-based and b) direct magnetic torque-based, and c) the reaction-based from the lumen walls. Here we explain the modeling for each component.

2.1 Torque from the magnetic pulling force $\boldsymbol{F}_{\mathbf{mag}}$

There is always a pulling force F_{mag} on the robot pointing to the magnet's center when it leads the robot. F_{mag} induces a torque bending the robot towards route (c) – (d), as shown in Supplementary Figure 14a. The maximum of F_{mag} gives $F_{\mathrm{mag}} = 3.6 \times 10^{-4}$ N, at a distance of 0.023 m away from the robot in the global $x - y$ plane. The resultant magnetic torque bending towards route (c) – (d) can be approximated as

$$T_{\mathrm{mag\_force}} = F_{\mathrm{mag}} \sin\left(\frac{\pi}{4}\right)\frac{l_{\mathrm{r}}}{2} = 6.3 \times 10^{-7}\ \mathrm{Nm}. \tag{5}$$

When the magnet is not leading the robot, F_{mag} is zero. Thus, the range of $T_{\mathrm{mag\_force}}$ is $[0, 6.3 \times 10^{-7}]$. This component is constant for curved routes with different radii of curvature R_{c}.

2.2 Magnetic torque $\boldsymbol{T}_{\mathbf{mag}}$

Due to the reorientation and rotation of the magnet, there is a rotating vector of magnetic flux density along the $x_{\mathrm{a}} - z_{\mathrm{a}}$ plane. This vector induces the magnetic torque T_{mag}, aligning the robot to the magnet (Supplementary Figure 14b). The maximum T_{mag} can be acquired when the magnet

is not leading the robot, where the maximum flux density along the x_a-axis is $B_{x\_max} = 6.0 \times 10^{-2}$ T. T_{mag} can then be computed as

$$T_{mag} = \mathbf{m}_r \times B_{x\_max} = |\mathbf{m}_r||B_{x\_max}| \sin\left(\frac{\pi}{2}\right) = 7.8 \times 10^{-6} \text{ Nm}, \quad (6)$$

where $\mathbf{m}_r$ is the magnetic moment of the robot. The minimum T_{mag} can be acquired when the magnet leads the robot for 0.023 m, i.e., the maximum of F_{mag} is acquired. Here, the minimum flux density $B_{x\_min} = 4.9 \times 10^{-2}$ T, then

$$T_{mag} = \mathbf{m}_r \times B_{x\_min} = |\mathbf{m}_r||B_{x\_min}| \sin\left(\frac{\pi}{2}\right) = 6.3 \times 10^{-6} \text{ Nm}. \quad (7)$$

Thus, the range of T_{mag} is $[6.3 \times 10^{-6}, 7.8 \times 10^{-6}]$. This component is uniform for curved routes with different R_c.

2.3 Torque from the reaction force F_{react}

When the robot starts to enter the curved route, the reaction force F_{react} from the lumen wall is applied to the robot. As shown in Supplementary Figure 14c, as the robot rotates around the positive y_r-axis and tends to roll to the positive x_r-axis, F_{react} points to the negative x_r-axis. The resultant torque T_{react} bends the robot towards (c) – (d). For the simplification of illustration, the robot can be regarded as a cantilever beam to compute T_{react}. First, by geometric approximation, the bending angle β can be computed as

$$\beta = \frac{l_{in}}{R_c}, \quad (8)$$

where l_{in} is the length of robot into the curved routes. Meanwhile, based on the cantilever beam model, β can be represented as

$$\beta = \frac{T_{react}\, l_{in}}{E_r I_r}, \quad (9)$$

where I_r is the second moment of area for the cross-section undergoing bending. Thus, the T_{react} can be finally computed as

$$T_{react} = \frac{E_r I_r}{R_c} = E_r I_r \kappa_c. \quad (10)$$

Thus, this component is various for curved routes with different R_c.

We first compare the magnet-based torques and T_{min}, as shown in Supplementary Figure 14d. with the decreasing of R_{c} (increasing of κ_{c}), the gap between T_{min} and the torques are more significant, which needs to be enabled by T_{react} from the proper rotation direction of the robot. Together with T_{react}, the sum of these torques are superior to the T_{min} among the physiologically relevant range (Supplementary Figure 14e), enabling the successful curved routes traversing.

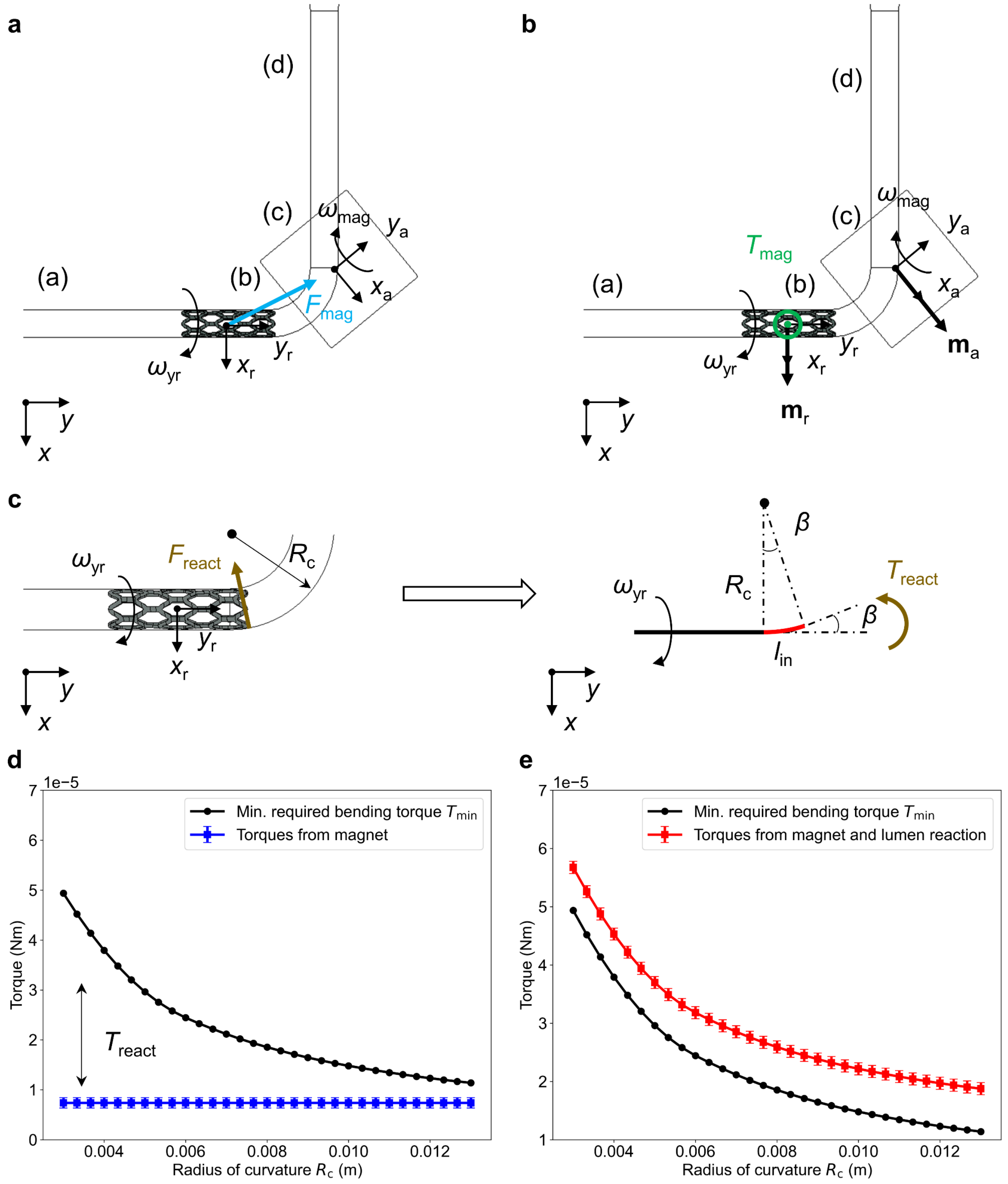

Supplementary Figure 14. Analyses of the torques enabling the traversing among curved routes. **a** Magnetic force-induced torque due to the leading position of the magnet. **b** Magnetic torque due to the reorientation of the rotating magnet. **c** Reaction force-induced torque from the lumen wall. **d** Quantifications on the minimum required bending torques T_{min} and the magnet-

based bending torques. The gap indicates the component enabled from the lumen wall reaction. **e** Quantifications on T_{min} and all bending torques. The superiority of actuation torques enables the curved route traversing among the physiologically relevant range. Note that the error bars indicate the maximum and minimum values. In **d** and **e**, the components of magnetic torques are represented as the average torque along the magnet movement ± half of the range for the achievable torque according to the force modeling.

Supplementary Note 3. Quantification of torques enabling the traversing among bifurcations

3.1 Torque from the magnetic pulling force $\boldsymbol{F}_{\mathbf{mag}}$

The torques generated from F_{mag} is different among various bifurcation angles θ_{b} (Supplementary Figure 15a), which can be approximated as

$$T_{\mathrm{mag\_force}} = F_{\mathrm{mag}} \sin\left(\frac{\theta_{\mathrm{b}}}{2}\right)\frac{l_{\mathrm{r}}}{2}, \tag{11}$$

where $F_{\mathrm{mag}} = 3.6 \times 10^{-4}$ N, at a distance of 0.023 m away from the robot in the global x-y plane. When the magnet is not leading the robot, $T_{\mathrm{mag\_force}}$ is zero.

3.2 Magnetic torque $\boldsymbol{T}_{\mathbf{mag}}$

T_{mag} is different among various bifurcation angles θ_{b} (Supplementary Figure 15b). The maximum T_{mag} can be acquired when the magnet is not leading the robot, where the maximum flux density along the x_{a}-axis is $B_{\mathrm{x\_max}} = 6.0 \times 10^{-2}$ T. T_{mag} can be computed as

$$T_{\mathrm{mag}} = \mathbf{m}_{\mathrm{r}} \times B_{\mathrm{x\_max}} = |\mathbf{m}_{\mathrm{r}}||B_{\mathrm{x_{max}}}| \sin\left(\frac{\theta_{\mathrm{b}}}{2}\right). \tag{12}$$

The minimum T_{mag} can be acquired when the magnet leads the robot for 0.023 m, i.e., the maximum F_{mag} is acquired. Here, the minimum flux density $B_{\mathrm{x\_min}} = 4.9 \times 10^{-2}$ T,

$$T_{\mathrm{mag}} = \mathbf{m}_{\mathrm{r}} \times B_{\mathrm{x\_min}} = |\mathbf{m}_{\mathrm{r}}||B_{\mathrm{x\_min}}| \sin\left(\frac{\theta_{\mathrm{b}}}{2}\right). \tag{13}$$

3.3 Torque from the reaction force $\boldsymbol{F}_{\mathbf{react}}$

When the robot starts to enter the bifurcation, the reaction force F_{react} from the lumen wall is applied to the robot. As shown in Supplementary Figure 15c, when the robot rotates around the positive y_{r}-axis and tends to roll to the positive x_{r}-axis, F_{react} points to the negative x_{r}-axis. The resultant torque T_{react} also bends the robot towards (c) – (d). We also regard the robot as a

cantilever beam to compute the T_{react} here. First, the bending angle β, i.e., $\beta = 0.5\theta_{\mathrm{b}}$, can be represented as

$$\beta = \frac{l_{\mathrm{in}}}{R_{\mathrm{c}}}, \tag{14}$$

where l_{in} is the length of robot into the bifurcation junction. Then, based on the cantilever beam model, β can be represented as

$$\beta = \frac{T_{\mathrm{react}}\, l_{\mathrm{in}}}{E_{\mathrm{r}} I_{\mathrm{r}}}. \tag{15}$$

Thus, the T_{react} can be computed as

$$T_{\mathrm{react}} = \frac{E_{\mathrm{r}} I_{\mathrm{r}}}{R_{\mathrm{c}}}. \tag{16}$$

To compute R_{c} for the bifurcations, we have made the geometric analysis, and the following relation can be used,

$$\sin(\beta) = \frac{l_{\mathrm{gap}}}{R_{\mathrm{c}}}, \tag{17}$$

where l_{gap} is the bifurcation gap length (Supplementary Figure 15d). It can be seen that this component is also various for bifurcations with different θ_{b}. Thus, T_{react} can be finally computed as

$$T_{\mathrm{react}} = \frac{E_{\mathrm{r}} I_{\mathrm{r}} \sin(\beta)}{l_{\mathrm{gap}}}. \tag{18}$$

We first quantify the magnet-based torques and compare them with T_{min}, as shown in Supplementary Figure 15e. Note that with the increasing of θ_{b}, the gap between T_{min} and the magnet-based torques are more significant, which needs to be enabled by the T_{react} from the proper rotation of the robot. Together with T_{react}, the sum of these torques is superior to the T_{min} among the physiologically relevant range, enabling the successful traversing among these routes (Supplementary Figure 15f). Certainly, these descriptive force modeling methods here are used to understand the basic underlying mechanics, and further finite element analyses will be carried out to explore the dynamics in future work.

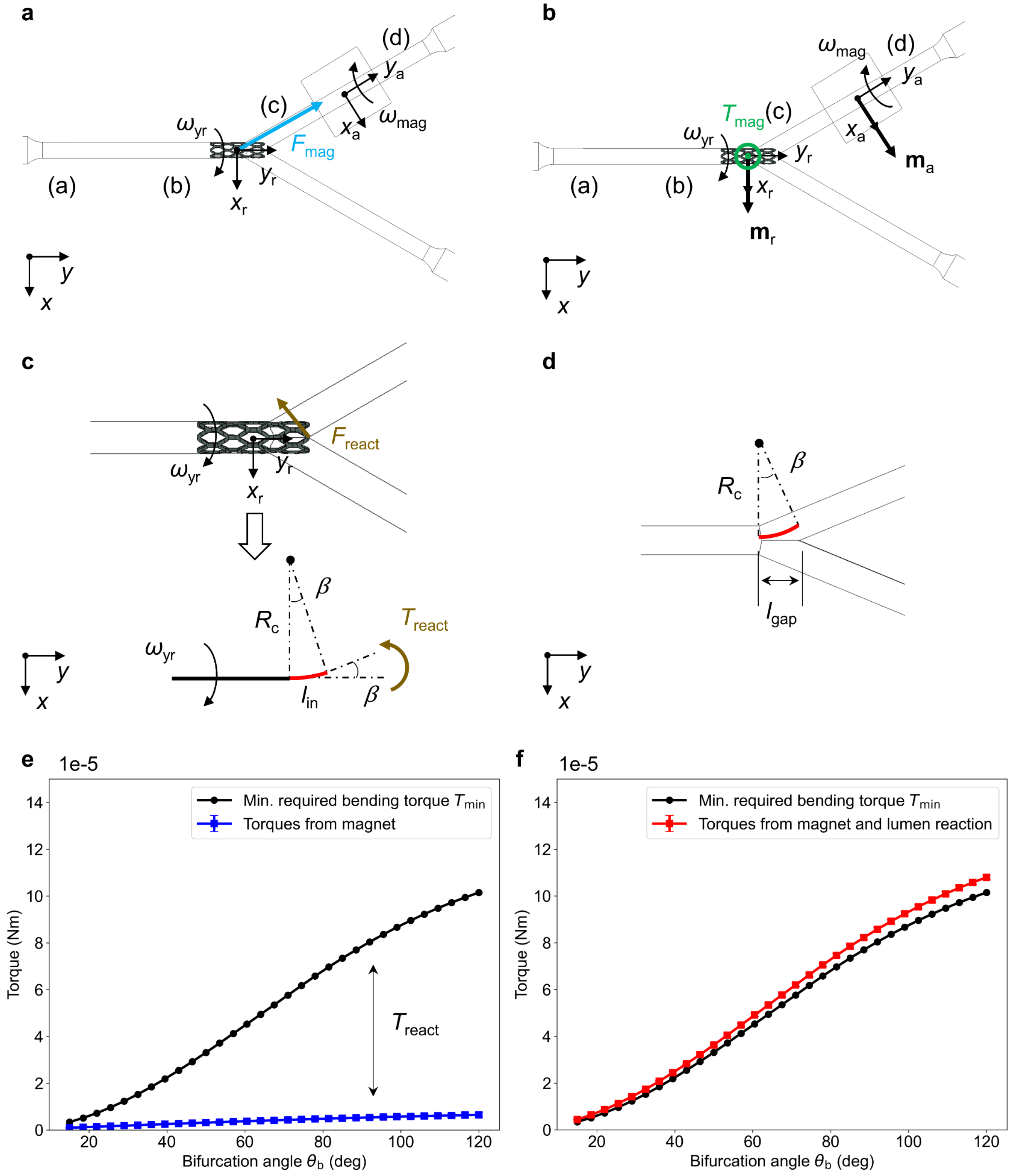

Supplementary Figure 15. Analyses of the torques enabling the traversing among branches. **a** Magnet force-induced torque due to the leading position of the magnet. **b** Magnetic torque due to the reorientation of the rotating magnet. **c** Reaction force-induced torque from the lumen wall. **d** Calculation of radius of curvature for bifurcations. **e** Quantifications on the minimum required

bending torques T_{min} and the magnet-based bending torques. The gap indicates the component from the lumen wall reaction. **f** Quantifications on T_{min} and all bending torques. The superiority of actuation torques enables the curved route traversing among the physiologically relevant range. Note that the error bars indicate the maximum and minimum values. In **e** and **f**, the components of magnetic torques are represented as the average torque along the magnet movement ± half of the range for the achievable torque according to the force modeling.

Supplementary Note 4. Compatibility with the commercial microcatheters for distal arteries

An even smaller version of the robot, e.g., initial diameter Φ_r = 0.7 mm, compatible with the microcatheter for distal arteries, e.g., ID = 0.03 inch, could achieve retrievable locomotion for lumen diameter range Φ_l = 1.5 mm to 0.2 mm. Particularly, for Φ_l = 1.5 mm to 0.7 mm, the robot has no shape adaptation; for Φ_l = 0.7 mm to 0.2 mm, the robot is shape-adaptive during locomotion. This claim was made through theoretical modeling in the previous response.

To experimentally validate such claims, we have fabricated the miniaturized version of the robot with Φ_r = 0.7 mm, length l_r = 2.5 mm (Supplementary Figure 16a), and the corresponding phantom T with Φ_l = 1.5 mm to 0.2 mm. As shown in experiments (Supplementary Figure 16b**)**, the miniaturized robot could be delivered by the microcatheter with ID = 0.03 inch and realized the retrievable locomotion in Φ_l range from 1.5 mm to 0.3 mm under the flow rate of 5.6 ml/min, which is calculated based on the flow into the distal arteries[26]. Moreover, the robot could safely withstand the flow in the deformed state, where flow was indicated by the black dye).

Accordingly, these experimental phenomena could be explained by the updated theoretical modeling. Taking the against flow case as an example, the superiority of maximum leading forces over the max resistant force ensures the retrievable locomotion for around Φ_l = 1.5 mm to 0.3 mm (Supplementary Figure 16c). Given the significant increase of fluidic drag at around Φ_l = 0.3 mm and lower, this superiority could not be guaranteed. For around Φ_l = 0.3 mm to 0.7 mm, self anchoring could also be ensured since the maximum static friction is superior to the fluidic drag in the corresponding lumen (Supplementary Figure 16d).

It should also be noted that for Φ_l = 1.5 mm to 0.7 mm when the robot is not fully in contact with the lumen, the superiority of the maximum leading forces is not very clear, as we have also observed from the experiments. These limitations could be practically resolved by balloon guide catheters (BGCs) to properly regulate the local flow (not necessary to block the flow fully) in the

future during the clinical translation. We expect the application lumen range of a single robot design to be enlarged with this combination. e.g., from 1.5 mm – 1.0 mm to 1.5 mm – 0.3 mm.

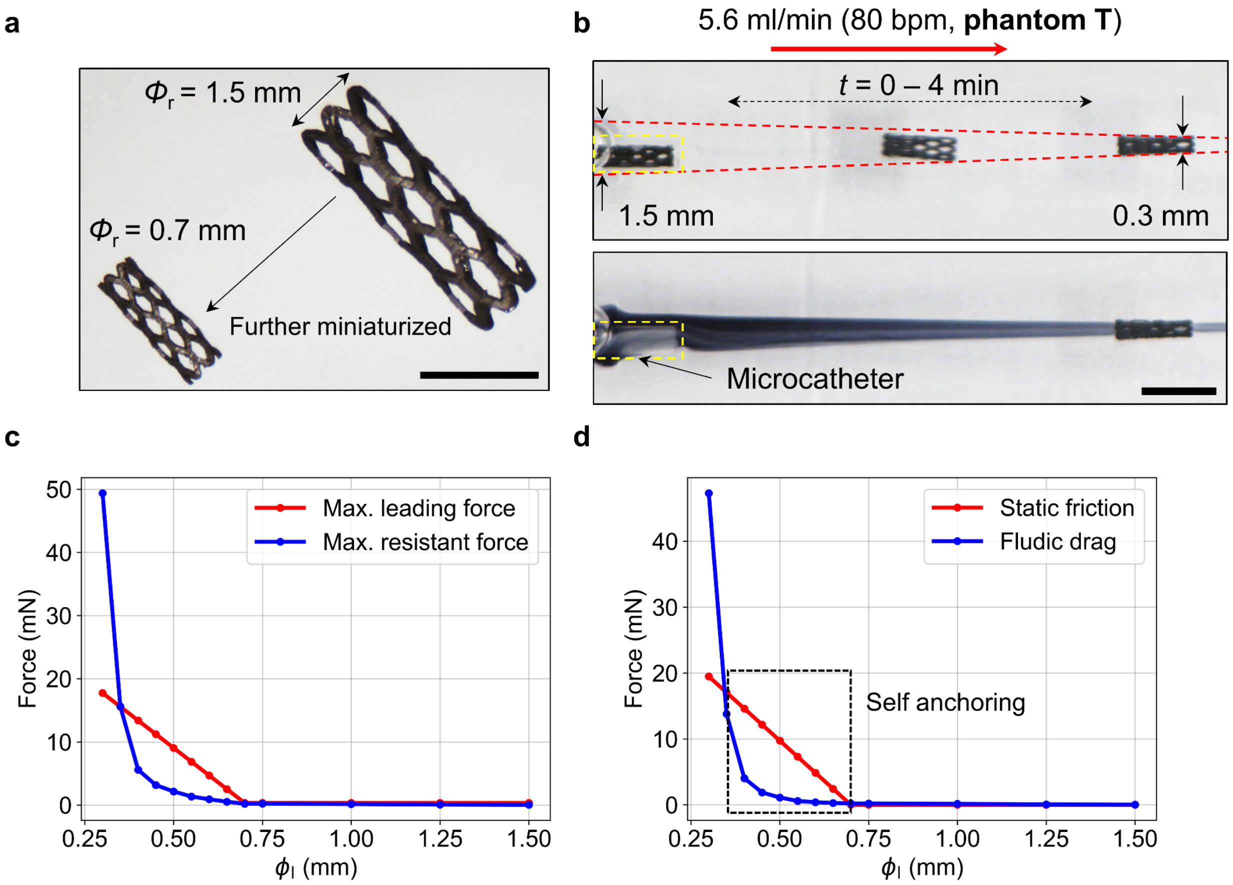

Supplementary Figure 16. Compatibility of the robot with the commercial catheter for the M4 segment. a Size comparison between the original robot and the miniaturized version with initial diameter Φ_r = 0.7 mm and length l_r = 2.5 mm. **b** Retrievable robot locomotion delivered by microcatheter (ID = 0.03 in, Medical Tubing, Nordson Medical) and the self-anchoring capability. **c** Theoretical explanation for the robot adaption limitation to around 0.3 mm (against the flow). **d** Theoretical explanation for the self-anchoring capability. l_{mag} = 50 mm and all scale bars are 2 mm.

Supplementary Note 5. Comparison with the standard neuro-interventional operations in distal arteries

As summarized in Supplementary Table S1, compared to the standard endovascular neuro-interventional operations in the distal cortical arteries, our stent-shaped magnetic soft robots could

be advantageous in the four following major aspects. We have conducted new experiments to confirm these superiorities:

- Accessibility with self-anchoring capability: better controlled retrievable navigation among the targeted distally tortuous routes given flow or no flow; even for the highly curved route with the inclination angle of 180°;
- Safety (in interaction forces): lower forces order (10^{-4} – 10^{-2} N) applied to the vessel walls compared to standard methods (10^{-1} – 10^{0} N);
- Drug dosage: reduced dosages given local delivery to minimize the systematic side effect;
- Adjustability: available to be adjusted as flow diverters after misplacement, dislodgement, or migrations[14-17].

The new phantoms were designed based on an angiography image of MCA M4 (Fig. 2.44 in ref[27] by Renan Uflacker) (Supplementary Figure 17a). Phantom O was based on the part that M4 comes out from the Sylvian fissure. Phantom K was based on the M4 angular artery on the cortex with some adaptations. The original anatomy was with a radius of curvature R_c of around 2 mm and an inclination angle γ_i of 150°. Here we designed phantom K with $R_c = 1$ mm and γ_i = 180°. The adaptation to more extreme conditions was intended to demonstrate the superior accessibility of our robot. It can be seen that the arteries in the section are highly tortuous, i.e., with small R_c and large γ_i. We compared the performance of the standard catheter-based approach and our robot in these physiologically relevant phantoms.

5.1 Accessibility

To access the target region using the standard endovascular neuro-interventional operations, the guidewire is first inserted from the femoral or radial artery and manipulated by pushing and torquing from the operator at the proximal end. After the guidewire is placed, the catheter will be delivered into the vessel coaxially. Due to the longer access route, touristy, and the smaller vessels with thinner vessel walls, catheterization into the distal arteries is often technically challenging[2,7]. In our experiments conducted with the assistance of an experienced neuroradiologist, we have observed the same phenomenon. We first utilized the combination of the micro guidewire (OD = 0.008 in, ASAHI CHIKAI 008, ASAHI, Japan; denoted as guidewire A hereafter) and medical tubing with the size compatible with the distal MCA (ID = 0.023 in, Medical Tubing, Nordson

Medical, USA; denoted as catheter N hereafter) and advanced into the phantoms O and K. Although the guidewire A could be delivered, co-axial delivery of microcatheters is challenging given the sharp turns (Supplementary Figure 17b). Here we have used glycerol in the lumen to lubricate the inner wall and reduce the kinetic coefficient of frictions (CoF) to a comparable value as the realistic case, i.e., around 0.07[28] (Supplementary Table S2).

We further conducted the insertion experiments with one of the smallest commercial microcatheters on the market, Magic 1.2 F (OD = 1.2 F, Balt Group, France; denoted as catheter B hereafter), using the same two-step paradigm (Supplementary Figure 17c). With the extra soft tip and hydrophilic surface coating, the microcatheter could be navigated inside for phantom O. However, the successful pass of the sharp turn cannot be guaranteed for phantom K due to the floppiness and mechanical instability. Our experienced neuroradiologist has attempted five times in total, with successful passing two times, which cost more than 5 minutes on average. Moreover, these two successful cases rely on the proximal force and torque applying near the inlet of the phantom, which could be unrealistic clinically. In summary, our experiments indicate that catheterization into phantom K is challenging. This hard-to-deliver phenomenon aligns with a reported study in a published high-impact article on Nature Communications[29] (please refer to Supplementary Figure 15 in the article for details).

In these highly curved phantoms, our stent-shaped magnetic soft robot could achieve effortless retrievable locomotion, given flow or no flow (Supplementary Figure 17d). Particularly, by shortening the designed length of the robot, the prototype with two cells along the body can even pass the extremely tortuous route with R_c = 1 mm and γ_i = 180° within 3 mins (one way; 6 mins for retrievable access). When translated to the clinical side, the dimensions of the stent-shaped robot, i.e., diameter and length, should be decided rigorously according to the geometrical features of the target region.

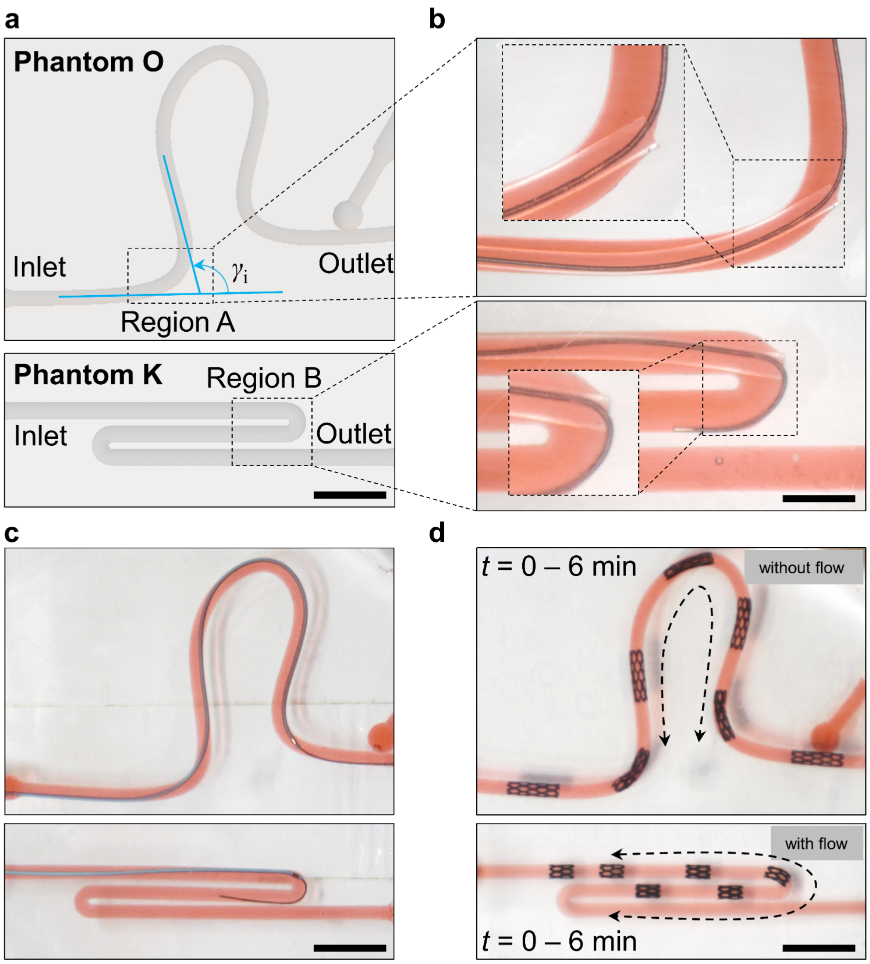

Supplementary Figure 17. Accessibility into the tortuous route by microcatheter and stent-shaped magnetic soft robot. a Design of physiological relevant phantoms O and K based on the anatomy of MCA M4 shown in ref[27]. The upper one corresponds to the section exiting the Sylvian fissure ($R_c \geq 3$ mm, $\gamma_i \leq 120^{\circ}$), and the lower one corresponds to a section of the angular artery ($R_c = 1$ mm, $\gamma_i = 180^{\circ}$). **b & c** Challenging access by guidewires (OD = 0.008 in, ASAHI CHIKAI 008, ASAHI, Japan; denoted as guidewire A hereafter), medical tubing (ID = 0.023 in, Medical Tubing, Nordson Medical, USA; denoted as catheter N hereafter), and one of the smallest commercial microcatheters (OD = 1.2 F, Magic 1.2 F, Balt Group, France; denoted as catheter B hereafter). **d** Retrievable robot locomotion in these tortuous routes with the flow or without flow. Scale bars for a, c, and d: 5 mm; Scale bar for b: 1.5 mm.

5.2 Safety in interaction forces

To quantify the catheter–phantom interaction forces, we utilized the robotic arm's end-effector force and torque sensing function (force resolution: < 0.05 N, Franka Emika, Germany). The phantoms were fixed onto a customized platform, which was connected to the arm's end-effector. We recorded the real-time force measurements (update rate: 30 Hz) along the global x and y-axes on the plane of guidewire and catheter delivery into phantoms O and K (Supplementary Figure 18a). Consistent with the accessibility tests, two sets of devices were investigated, i.e., 1) guidewire A + catheter N; and 2) guidewire A + catheter B. The average maximum force amplitude during the insertion for these two sets is around 0.84 ± 0.11 N (Supplementary Figure 18b). The real-time data is shown in Supplementary Figure 18c, and the A + N induced notable forces in both phantoms from the attempt to push forward the microcatheters and interact with the curved routes (1st and 2nd row). Given the floppy tip design of the A + B, the initial insertion into phantom O did not induce large force. However, to maintain the mechanical stability of the device, the more proximal segment of B is stiffer and insertion of these segments into phantom O started to induce notable resistive forces (3rd row). Inserting A + B and passing the sharp turn in phantom K was not feasible given the tip floppiness, and no notable forces could be observed (4th row). Given the number of contact points from 1 to 4 in different phantoms, the average force applied to each point is on the order of 10^{-1} to 10^{0}. The values are consistent with the reported ones in ref[30], ranging from 0.5 N to 3 N, depending on the expertise of the operators.

In contrast, our stent-shaped magnetic soft robot exerted lower forces on the phantoms, which is on the order of 10^{-4} to 10^{-2} N (Supplementary Figure 18b), according to the validated force modeling. Specifically, there are three groups of interaction forces, i.e., the axial forces applied to the wall at turns due to magnetic pulling force, whose maximum is 5×10^{-4} N at l_{mag} = 55 mm; the radial forces due to robot deformation, whose maximum is 4.5×10^{-2} N at the largest deformed state; and the friction forces, whose maximum is 3.6×10^{-3} N. The maximum forces correspond to the compressive stress of around 5.5 kPa, smaller than the quantified threshold to rupture the endothelial cell at around 12.4 kPa[31,32].

As stated in the literature[2] and the manual for using the neurovascular guidewire, e.g., ASAHI CHIKAI 008 neurovascular guidewire, "if any resistance is felt during insertion into a catheter used with the guidewire, please do not use it" and "adverse effects of vessel dissection,

perforation, and vasospasm etc," the safety of catheterization into these distal regions cannot always be guaranteed given the notable forces. In contrast, given the low interaction forces and no sign of wall deformation observed (Supplementary Figure 18d), our robots could be a promising alternative to enable safer interactions with the vessel walls for therapies in the distal arteries.

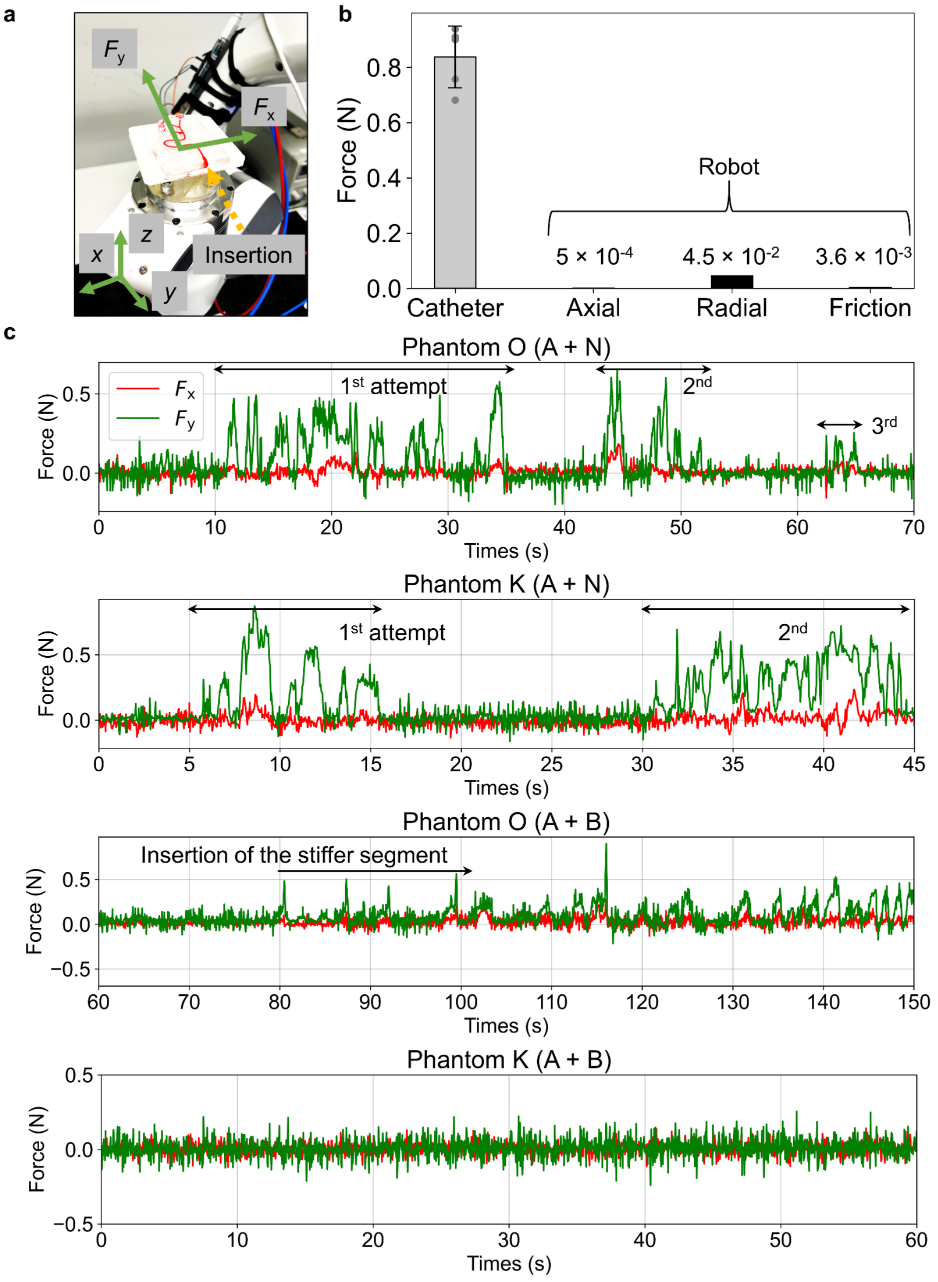

Supplementary Figure 18. Quantification of the interaction forces with the phantoms by microcatheter and stent-shaped magnetic soft robot. a Experiment setup to measure the interaction forces between catheter and phantom. **b** Comparison in the amplitudes of the maximum

forces by microcatheters and the robot. The data on the catheter-based approach are represented as the mean values ± standard deviation for $n = 5$. **c** Real-time force measurements (30 Hz) during advancement of 1) guidewire A + catheter N; and 2) the guidewire A + catheter B in phantoms O and K, respectively.

5.3 Drug dosage

Given the limitations in accessibility and safe interaction forces, local drug delivery in the vicinity of distal lesioned areas by standard intra-arterial catheterization is challenging, consequently increasing the necessary dosage to treat the diseases in the distal arteries and the risks of systematic complications[5,6,9,10,19]. To explain this scenario, we have fabricated phantom P as a variant of phantom O, where the inlet bifurcates into two branches, branch A and branch B (Supplementary Figure 19a). This is a realistic extension given multiple branches in the M4 segment[27].

We assumed a total occlusion in the distal end of branch A, and all incoming flow from the inlet was distributed into branch B, as indicated by the black dye in Supplementary Figure 19b. As seen from the two sections above, further advancement of microcatheter into branch A to treat the occlusion by the drug was challenging and risky given the notable interaction forces in this physiologically relevant setting. Therefore, releasing the drug in the entry part of branch A is a compromised but safe solution (Supplementary Figure 19c). Here we injected 0.3 ml fluorescein sodium solution (4 wt% in PBS, 12 mg fluorescein sodium was thus applied), and it can be seen that the incoming flow has drifted quite a few into branch B. Consequently, diffusion into the distal blocked area was ineffective even after 11 mins.

In contrast, with the advantages of accessibility and safe interaction forces, the stent-shaped robot could approach the vicinity of the target within 4 mins (Supplementary Figure 19d) and release fluorescein sodium of 1.2 mg locally with a notable color contrast (Supplementary Figure 19e), thus reducing the possibly high dosage applied to the entry part of branch A in order to diffuse to the distal end. Although we used the specific occlusion example, the same concept could be extrapolated to other diseases, such as arteriovenous malformation, dural arteriovenous fistulas, and brain tumors, where the local applications of the drug could reduce the dosage and reduce systematic complications.

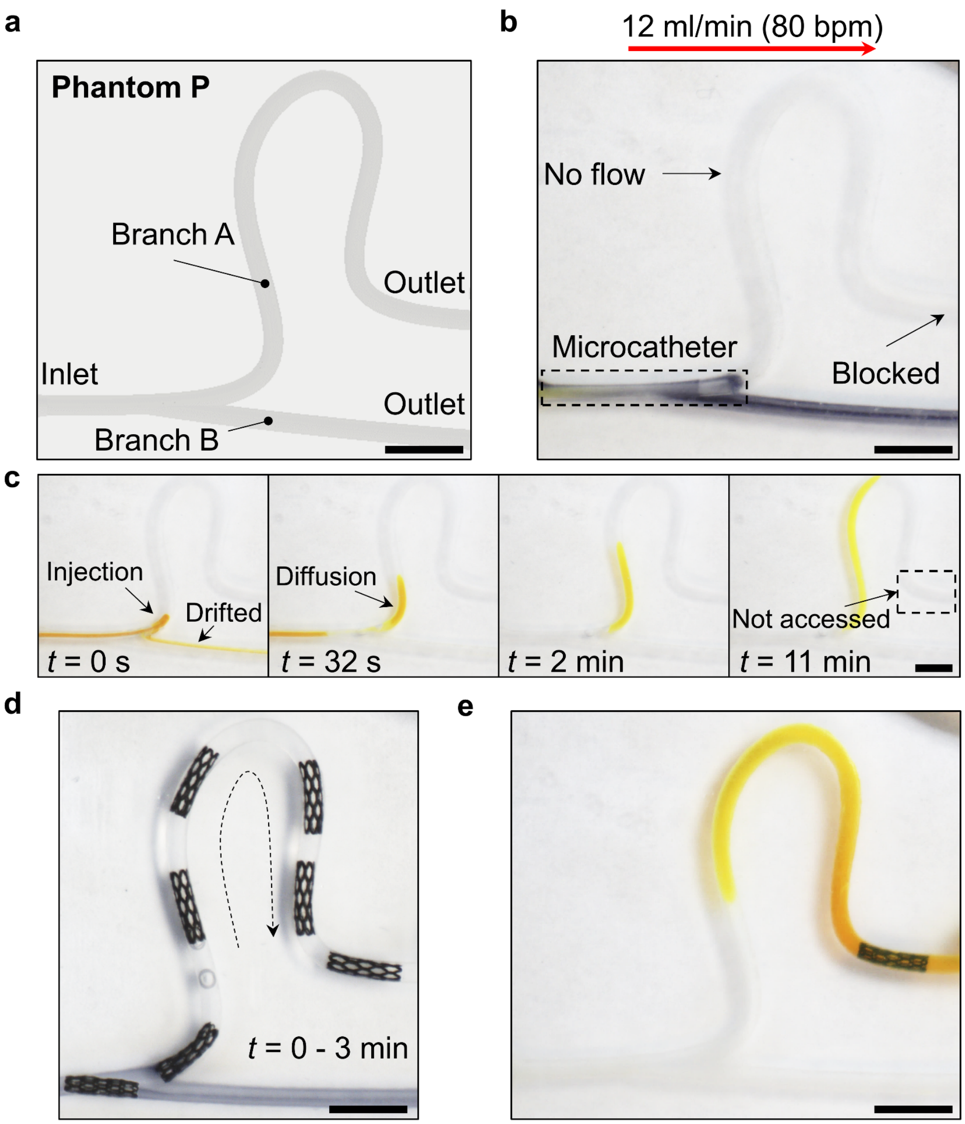

Supplementary Figure 19. Drug delivery by microcatheter and stent-shaped magnetic soft robot. a Phantom P with two branches as an extension of phantom O. **b** Flow visualization by black dye, where the flow into branch A was stopped by a distal blockage. **c** Injected drug (fluorescein sodium solution as the simulant) of around 12 mg with a negligible amount diffused into the distal blockage region. **d** Accessibility of the stent-shaped robot into the distal region. **e** Local drug applications of around 1.2 mg by the robot. All scale bars: 5 mm.

5.4 Adjustability

The stent-shaped magnetic soft robot could function as an actively-controlled flow diverter to deal with the complications of stent misplacement, dislodgement, and migrations for the therapies of treating wide-neck aneurysms, as we have shown in Figures. 6e – 6g.

Supplementary Note 6. Extension to proximal arteries

The newly fabricated phantoms Q – S mimics the dimension ranges for each MCA M1 – M3 segment, with Φ_l ranging from around 2.7 mm to 2.4 mm, 2,4 mm to 2.0 mm, and 2.0 mm to 1.5 mm, respectively[2] (Supplementary Figure 20a). Three more compatible robots for these three segments were also fabricated, where the robot length and the dimension of each composing diamond-shaped cell are currently identical (Supplementary Figure 20b). We experimentally investigated the relationship between l_{mag_max} and Φ_l in each phantom (Supplementary Figure 20c). The results are consistent with the theoretical calculations in both trends and amplitudes (Supplementary Figures 20d and 20e). Therefore, we utilized this validated model in phantoms and the measured CoFs of porcine arteries (Supplementary Table 2) to predict the robot's radial shape adaptative locomotion in vessels with various Φ_l. Particularly, we investigated whether l_{mag_max} would violate the physical constraint l_{mag_min}, i.e., the minimum allowed distance between the magnet and the targeted segment due to the constraint of the scalp. Taking the M1 segment as an example, l_{mag_max} ranges from around 16 cm to 11 cm for Φ_l = 2.7 mm to 2.4 mm. The corresponding l_{mag_min} ranges from 12.5 cm to 8.5 cm, smaller than l_{mag_max} for all Φ_l, indicating the feasibility of utilizing the robot in this proximal region (red lines in Supplementary Figure 20f). The same phenomenon also holds for M2 and M3 segments. Based on the above results, we suggest retrievably shape-adaptive locomotion could be feasible using the 50 mm cubic magnet in the proximal region.

Please note that, unlike the distal regions, without proper flow regulations, the wireless locomotion in the proximal region could be unsafe given the high-speed pulsatile flow (e.g., 151 ml/min into the one side M1 segment[26]) and the small kinetic friction between the robot and the lumen (around 0.07 (Supplementary Table 2)) during the robot movement. The risk can be practically avoided by combining our robot with the balloon guide catheters (BGCs) in the future, which enables the flow arrest during the retrieval of blood clots by stent retrievers or aspiration devices to avoid clots fragment and distal embolization[33] (flow rate was thus regulated to 12

ml/min for all the above investigations on locomotions). However, after the robot stops at the target location and functions as a flow diverter to treat the aneurysm, the flow needs to be recovered, and the robot needs to be self-anchored. Accordingly, we experimentally confirmed that the robot could withstand the realistic flow rate at the rest state. An example result is shown in Supplementary Figure 21a from phantom R that the robot could be self-anchoring to be against the realistic pulsatile flow for the M2 segment of around 50.2 ml/min[26]. This phenomenon could be explained by the relationships between frictions and fluidic drag. We utilized the validated model to predict that the robots could withstand the flow in M1 – M3 segments (Supplementary Figure 21b). For example, in the M1 segment, as long as the robot is adaptive to the lumen with Φ_l < 2.65 mm, the frictions are larger than the drag, enabling the self anchoring.

During the clinical treatment of wide-neck aneurysms, the endovascular stent placement through catheters can divert the flow directly or assist the coiling inside the sac. However, one potential problem of this therapy is stent dislodgement or displacement during the operations[14,15] or migration after the surgery[16]. These fully-deployed stents are nearly impossible to adjust, although some most advanced embolization devices, such as PIPELINE™ FLEX allows resheathable up to two full cycles during the deployment (https://www.medtronic.com/us-en/index.html). The current solution to these complications is to either manage the follow-up endovascular operations again to place another stent[16] or conduct the open surgical clipping[14]. In contrast, our stent-shaped wireless magnetic soft robot has the potential to be adjusted directly by magnetic actuation without further implantable devices. As shown above, our quantified experiments on locomotion capability in the phantoms Q – S validate such feasibility. Furthermore, to be more concrete, a new phantom T with physiologically relevant features of the M1 segment (Φ_l = 2.4 mm – 2.7 mm, the radius of curvature R_c = 6 mm)[27] was also fabricated to demonstrate that locomotion around an aneurysm is feasible (Supplementary Figures 21c and 21d). Please note that the safe and successful locomotion requires flow arrest in the proximal segments, as indicated in the previous section. This can be realized by combining our robot with the commercial BGCs for clinical translations towards proximal regions.

We validated the extension to proximal arteries and potential functional improvement through new experiments and analyses. With many different physiological features such as route curvature, bifurcation angles, and flow conditions in each proximal segment, e.g., M1 – M3, we suggest the role of our robot and the effects of interactive physiological features on its function

should be carefully reconsidered. For example, the M1 arteries are not as torturous as those seen in the distal segments and can be easier accessed by standard catheterization. Consequently, the locomotion capabilities to traverse among various curved routes and branches are not necessary. Meanwhile, its adjustability, combined with the BGCs to regulate the flow, could be an essential supplementary function to the current standard endovascular operations. Moreover, given the shorter distance to cardiac pumping, the proximal segments are more prone to be influenced by pulsatility and pressure fluctuations[34]. In contrast, going deeper into M2 and M3, we require more and more locomotion capability due to the reduction of catheter accessibility, and the segments are less influenced by pulsatile flow due to the longer routes to dampen the pulsatility further. These detailed proximal segment-dependent differences should be individually investigated as a future direction.

a

Segment in MCA	Distance to the scalp (mm)	Φ_l range (mm)	Robot initial diameter (mm)	Estimated m_r (A·m^2)
M1	90	2.4 – 2.7	2.7	2.33×10^{-4}
M2	50	2 – 2.4	2.4	2.12×10^{-4}
M3	35	1.5 – 2	2	1.69×10^{-4}
M4	15	1 – 1.5	1.5	1.27×10^{-4}

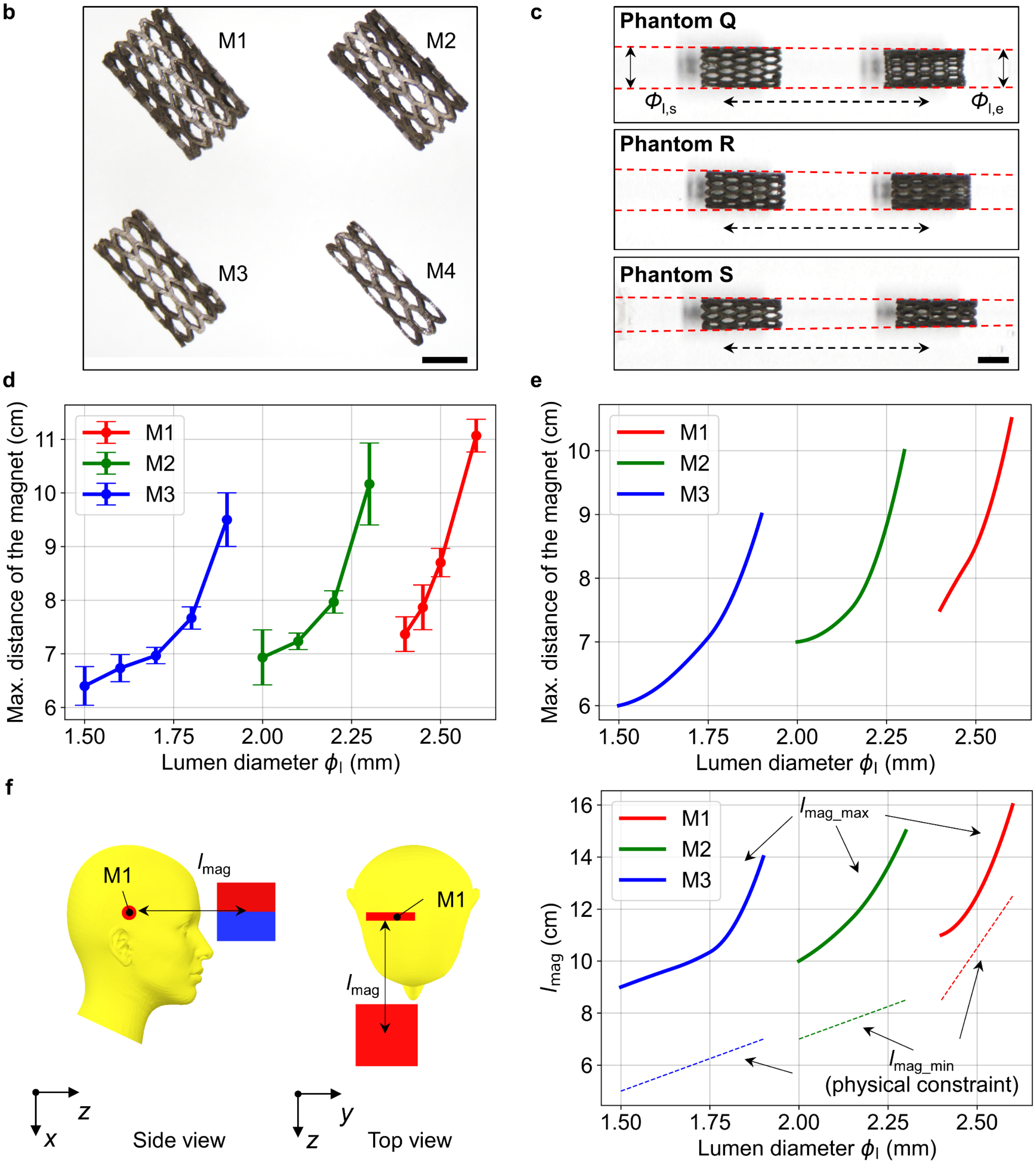

Supplementary Figure 20. Feasibility of extension to proximal arteries. a Physiological features and robot design parameters for different segments in MCA[2,35-37]. **b** Robots for M1 – M4 segments. The composing diamond-shaped cells and the overall length are identical. **c** Retrievably shape-adaptive locomotion in phantoms Q – S corresponding to the M1 – M3 segments (lumen diameter Φ_l: 2.7 – 2.4 mm, 2.4 – 2 mm, 2 – 1.5 mm). The flow rate was regulated to 12 ml/min, which could be realized by balloon guide catheters (BGCs). **d** & **e** Experimental and modeling results of the relationship between the maximum allowed distance of the magnet for retrievably shape-adaptative locomotion l_{mag_max} and the corresponding Φ_l. In **d**, the data are presented as mean values ± standard deviation for n = 5. **f** Predicted relationship between l_{mag_max} and l_{mag_min} along with Φ_l in arteries. The head model was reproduced from "Head And Skkull" by parsanouri under the CC BY 4.0 license (https://skfb.ly/6SWwZ; http://creativecommons.org/licenses/by/4.0/). All scale bars: 2 mm.

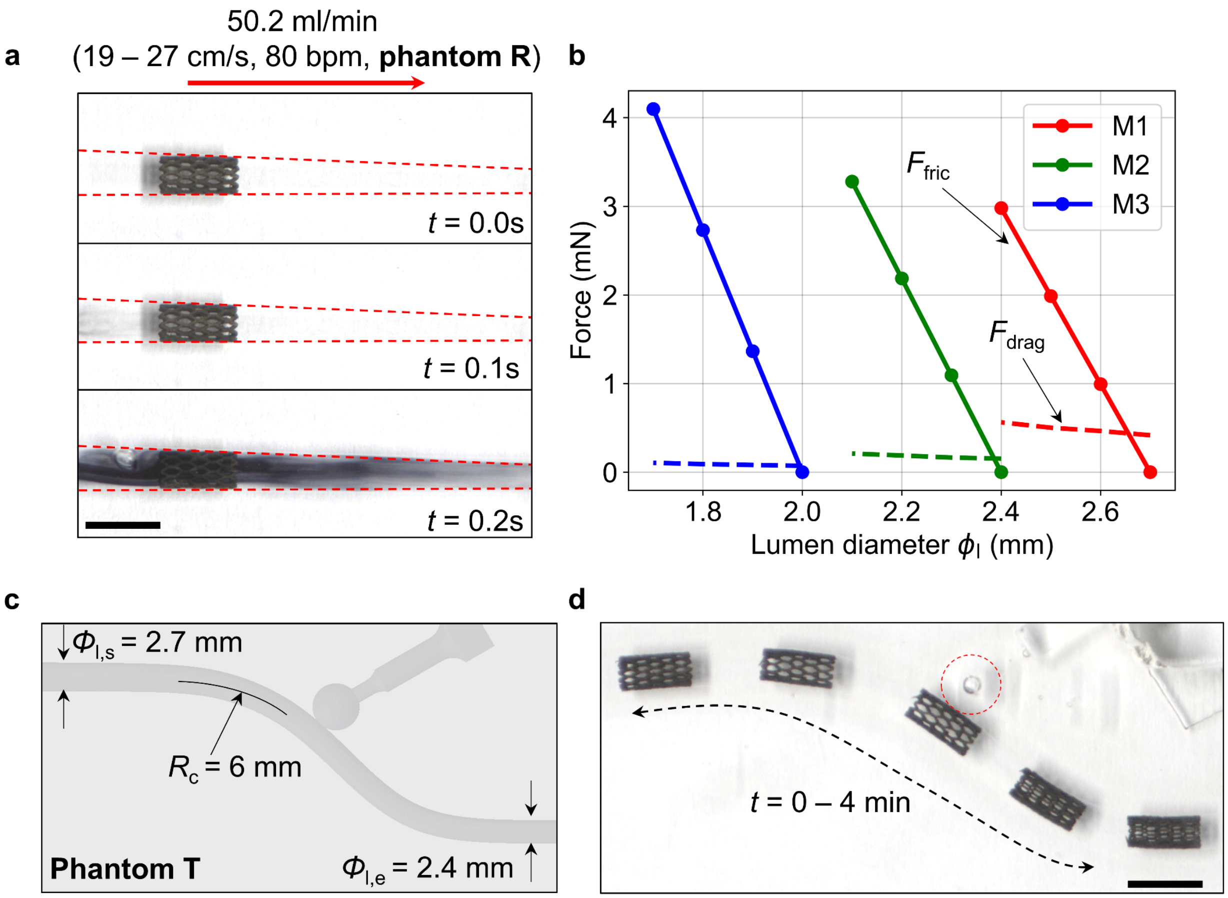

Supplementary Figure 21. Self anchoring and adjustability in the proximal arteries. a Self-anchoring capability of the robot under high-speed pulsatile flow. **b** Theoretical explanation for

self-anchoring capability in arteries. **c & d** Locomotion-enabled adjustability in phantom T. The function can be used as a follow-up treatment for stent misplacement, dislodgement, and migrations. All scale bars: 5 mm.

Supplementary Note 7. Compatibility with the C-arm X-ray fluoroscopy

The robot actuation can be compatible with the commercially available advanced C-arm X-ray fluoroscopy using a single permanent magnet, e.g., the ARTIS icono bi-plane angiography systems from Simens Healthineers. For the robot actuation in the M1 – M2 segments, the permanent magnet can be manipulated at the caudal side of the brain, minimizing the robot blocking by the magnet during scanning (Supplementary Figure 22a). For robot actuation in the M3 – M4 segments, the magnet can be manipulated at the lateral side of the brain, which will also have a minimal negative effect on the visualization of the robot (Supplementary Figure 22b). Please note that this is the preliminary plan for the clinical usage of the device, which needs to be further evaluated for various segments individually.

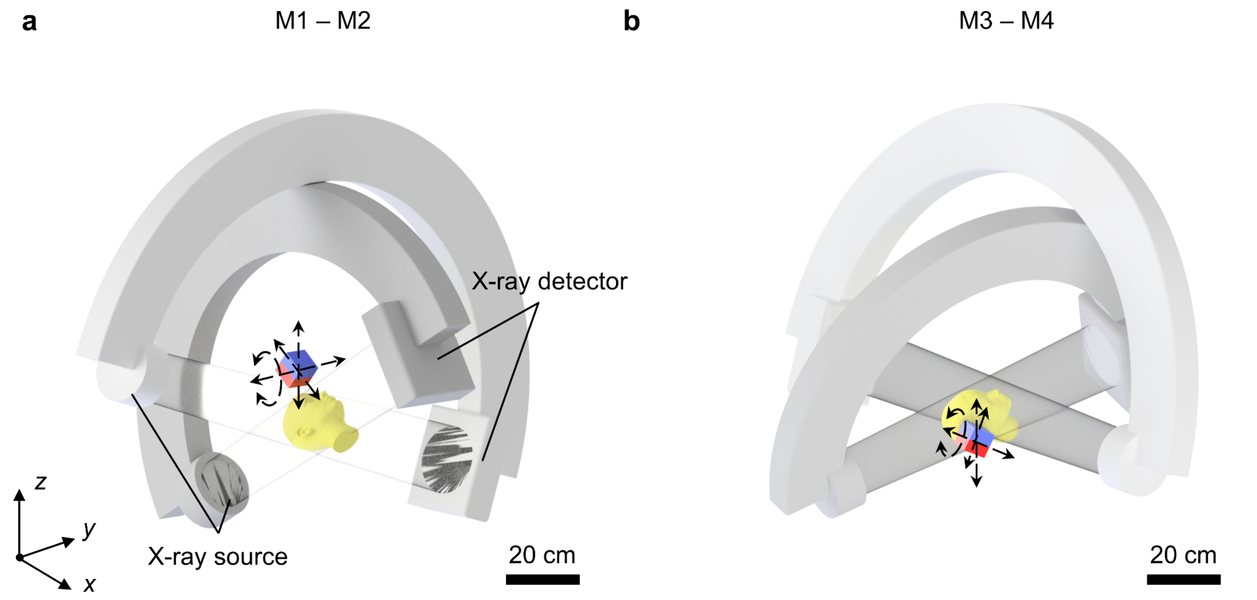

Supplementary Figure 22. Compatibility of the permanent magnet actuation with the bi-plane C-arm fluoroscopy. The cubic magnet with 10 cm is shown here. The head model was reproduced from “Head And Skkull” by parsanouri under the CC BY 4.0 license (https://skfb.ly/6SWwZ; http://creativecommons.org/licenses/by/4.0/). **a** Robot actuation and imaging for M1 – M2 segments. **b** Robot actuation and imaging for the M3 – M4 segments.

Supplementary Note 8. Evaluation and improvement of biocompatibility and hemocompatibility

8.1 Coatings to improve biocompatibility and hemocompatibility

PDMS is well-known for its biocompatibility with no acute cytotoxicity[38], but it is not hemocompatible because of its rapid adsorption of proteins and relatively high adhesion of blood platelets in blood-contacting applications[39-41]; NdFeB is not biocompatible due to its corrosive nature[42]. On the other hand, there is no reported data on the compatibility of the epoxy SMP used in this study, and iron-oxide (Fe_3O_4) nanoparticles have already been used for various blood-contact applications, such as MRI imaging contrast agents and targeted delivery of drugs[43].

After coating NdFeB microparticles with silica to improve the biocompatibility[44], we first quantified the magnetic hysteresis for the bare particles and the coated ones. The comparison indicated neglectable differences in magnetic properties for these samples (Supplementary Figure 23a). Next, we prepared the two groups of material composites, i.e., PDMS + NdFeB@SiO_2 and SMP + Fe_3O_4.

Various (coating) materials have been developed to improve the hemocompatibility of PDMS to be used for blood-contact medical devices, such as PDMS-based polyurethane-urea bearing zwitterion sulfobetaine (PDMS-SB-UU)[39], hyaluronic acid (HA) and polydopamine (PDA) composite (HA/PDA)[41], siloxane-bound poly(ethylene glycol)(PEG)[40], and Parylene C[45]. Particularly, Parylene C is FDA-approved and widely used in the conformal coating of implantable stents with complex structures, given its inert chemical property, pin-hole-free consistent thickness, and light weight[46] (Specialty Coating Systems, USA). Parylene C could also enable the drug-eluting stents with the controlled speed of drug release[47]. It is impermeable upon deposition to a thickness t_p = 1.4 nm (SCS, USA), and an ultra-thin Parylene film around t_p =100 nm could adjust the surface property of the substrate or protect the functional unit[48]. For commercial stents, deposited at 0.5 μm, Parylene C is also pinhole-free and can fully coat inside crevices and contours as small as 1 μm[47]. Following this guidance, we currently coated our material samples with t_p = 0.5 μm using a commercial deposition system (SCS Labcoter® 2 (PDS 2010), Specialty Coating Systems, USA). The validation of the proper coating thickness was conducted with the focused ion beam (FIB) microscopy. The contrast between the coated Parylene C layer and the substrate materials can be seen in Supplementary Figure 23b.

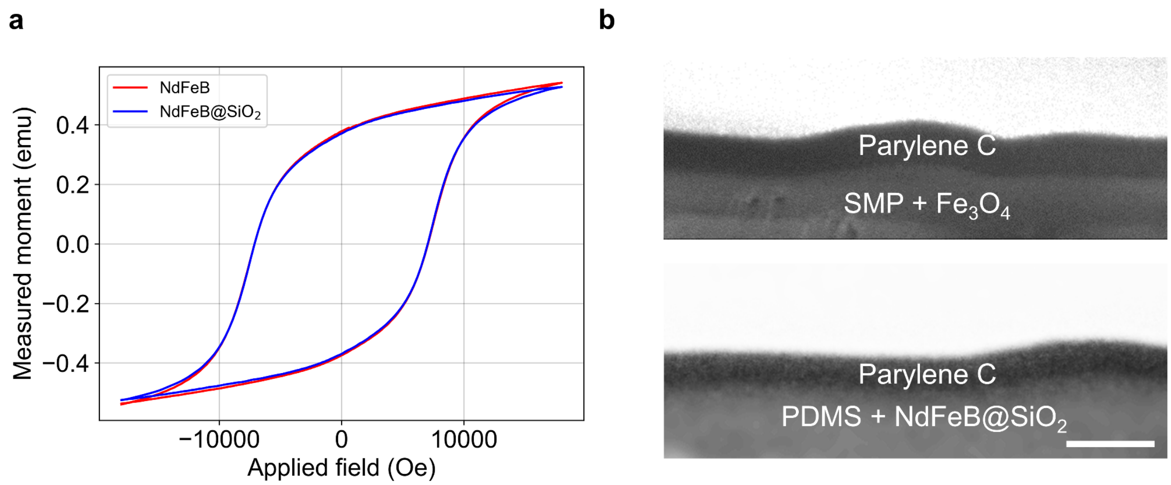

Supplementary Figure 23. Investigation of coating materials. a Magnetic hysteresis of the bare NdFeB microparticles and the silica-coated particles. Sample weights: 5 mg. **b** Investigations on the Parylene C coating using focused ion beam (FIB) microscopy. The thickness t_p is around 0.5 µm. Scale bars: 1 µm.

8.2 Biocompatibility

We prepared Parylene C-coated PDMS + NdFeB@SiO_2 and Parylene C-coated SMP + Fe_3O_4 samples and investigated their *in vitro* biocompatibility[29,49] using murine monocyte-macrophage cell line J774A.1 (ATCC). The tests results showed that cell viability was not negatively influenced for both samples, confirming the biocompatibility of the composite (Supplementary Figure 24). Please note that the composite of SMP and Fe_3O_4 is currently claimed to be used for drug delivery lasting from minutes to hours. Therefore the incubation time was 24 hours and was not evaluated for a longer time.

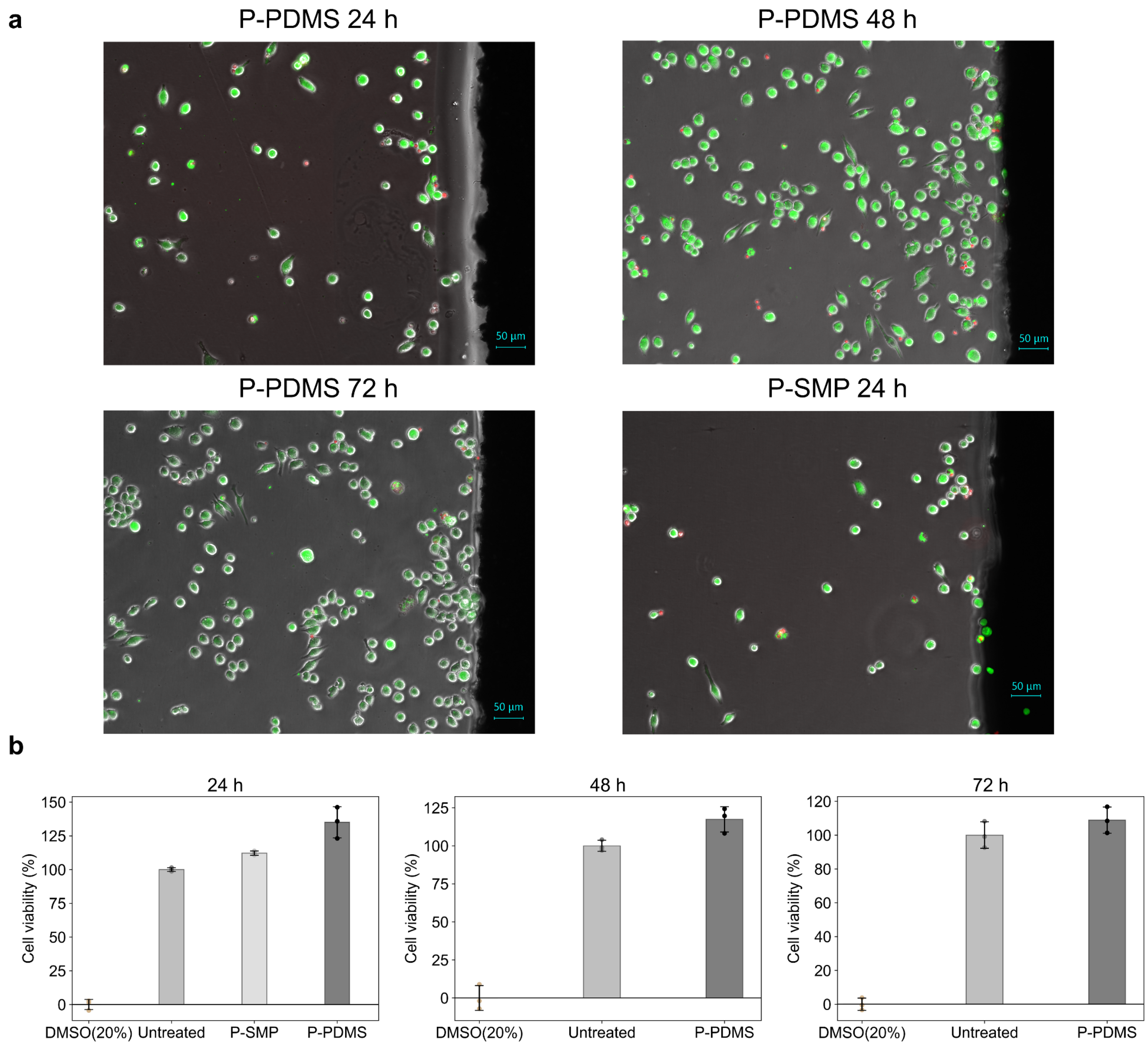

Supplementary Figure 24. Cell viability tests of Parylene C coated material samples. a Fluorescence microscopic images of the murine monocyte-macrophage cell line J774A.1 (ATCC) incubated with the composites at the exposure times of 24, 48, and 72 hours, respectively. Parylene C-coated PDMS + NdFeB@SiO_2 is denoted as P–PDMS. Parylene C-coated SMP + Fe_3O_4 is denoted as P–SMP. Sample size: 2 mm × 2mm × 0.1 mm. **b** Cell viability assessed using the LIVE/DEAD Cell Imaging Kit (Thermo Fisher Scientific, #R37601). The positive control was treated with 20% DMSO (Sigma-Aldrich), and the negative control was left untreated, i.e., no material samples were incubated. The cell viability was normalized between 100% viable for the negative control and 0% viable for the positive control. Fluorescence microscopic images were

taken at the eight corners for each of the three samples in each group. The data are presented as mean values ± standard deviation for $n = 3$.

8.3 Hemocompatibility

The hemocompatibility, including hematotoxicity and thrombogenicity studies, was evaluated using fresh rat blood. We investigated cell morphology by hematoxylin and eosin staining in the hematotoxicity studies. In the thrombogenicity studies, the luciferase assay was conducted to evaluate platelet activation[50]. In none of the materials samples, morphological changes in blood cells were observed after short-term agitated blood incubation (Supplementary Figure 25a), indicating no evidence of hematotoxicity. In addition, no significant thrombogenic activation was observed in each of these coated materials (Supplementary Figure 25b). These evaluations confirmed the good hemocompatibility of the Parylene-C-coated samples, aligned with the reported evaluations according to USP Class VI, ISO 10993, and RoHS standards[46,47].

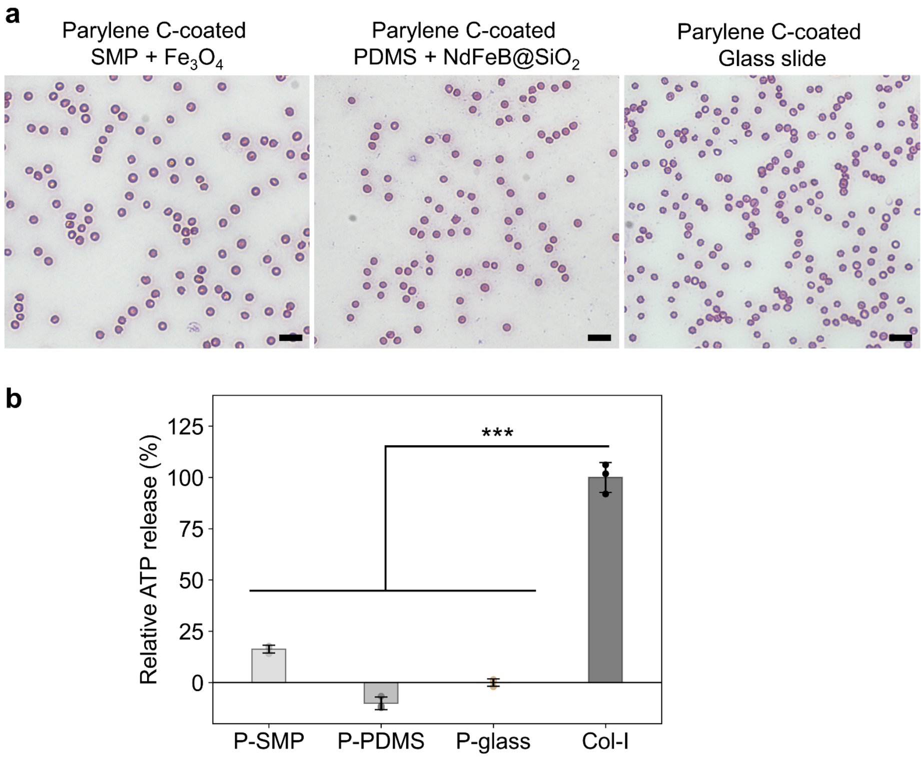

Supplementary Figure 25. Hemocompatibility tests on the Parylene C-coated robot materials. **a** Cell morphology inspection after hematoxylin and eosin staining. Scale bars: 20 µm. **b** Measurement of platelet activation using luciferase assay. The whole blood sample with collagen-I (100 µg/mL) was used as a positive control (100 %), and the whole blood sample incubated with the Parylene C-coated glass slide was used as a negative control (0 %). The relative ATP release

was normalized between the positive control and negative control. Values were compared among the positive control and other groups (****P* < 0.001; $P = 4.22 \times 10^{-5}$, 1.73×10^{-5}, and 2.06×10^{-5} for P-SMP, P-PDMS, and P-glass, respectively) by the one-way ANOVA test. Samples size: 2 mm × 2 mm × 0.1 mm. The data are presented as mean values ± standard deviation for $n = 3$.

8.4 Effects of Parylene C coating on locomotions and functions

We then coated Parylene C on the stent-shaped magnetic soft robot. The effect of Parylene C coating on reducing frictions is notable (Supplementary Figure 26a). Since Parylene C is with high Young's modulus at around 3.2 GPa[51], we experimentally optimized the robot's stiffness by tuning the robot's Young's modulus E_r and the thickness of Parylene C t_p, to find the combination that the robot is still flexible enough to fulfill all the locomotion requirements. To satisfy the necessity of $t_p = 0.5$ μm, we currently selected the design with $E_r = 5.0$ MPa (Supplementary Figure 26b). The experiments indicated that retrievably shape-adaptive locomotion, highly curved route traversing (R_c = 3 mm), and branch traversing ($\theta_b = 120^{\circ}$) could be appropriately maintained (Supplementary Figures 26c – S26e). For the foldable structure, we also experimentally observed that the coating does not influence its shape memory property (Supplementary Figure 26f).

8.5 Effect of deformation on the Parylene C coating

The function of the stent-shaped robot depends on its deformation, and these deformations might lead to the fracture of the Parylene C coating layer and exposure of the substrate, which is not ideal for hemocompatibility. To investigate the potential risk, we prepared the Parylene C coated samples for both composites (2 mm × 2 mm × 0.1 mm) and deformed them through bending (strain: 44.7%) and compression (stress: 5.5 kPa), as experienced by the robots during locomotion and functioning. Frictions were ignored due to their low order at 10^{-3} N. We then observed the surface morphology of the samples by SEM. It is clear that these deformations led to notable wrinkles on the coating layer compared to the undeformed samples (Supplementary Figure 27a). A detailed SEM investigation indicated that these wrinkles should still be continuous and not be the fractures of the coating layer (Supplementary Figure 27b). To further confirm this, we compounded the PDMS + NdFeB composite with the fluorescein sodium (mass ratio: 10:1) and coated the samples (5 mm × 5 mm × 0.1 mm) with Parylene C with $t_p = 0.5$ μm, then deformed them. The leaching tests were conducted on the uncoated sample (U), coated sample without deformation (C), and the coated sample with deformation (CF) in PBS (pH = 7.4, Gibco™, Thermo Fisher Scientific). After

5 days, no visible fluorescence in the solution of C and CF, in contrast to U, indicated that the coating layer is effective in impermeability, and the deformation did not expose the substrate (Supplementary Figure 27c).

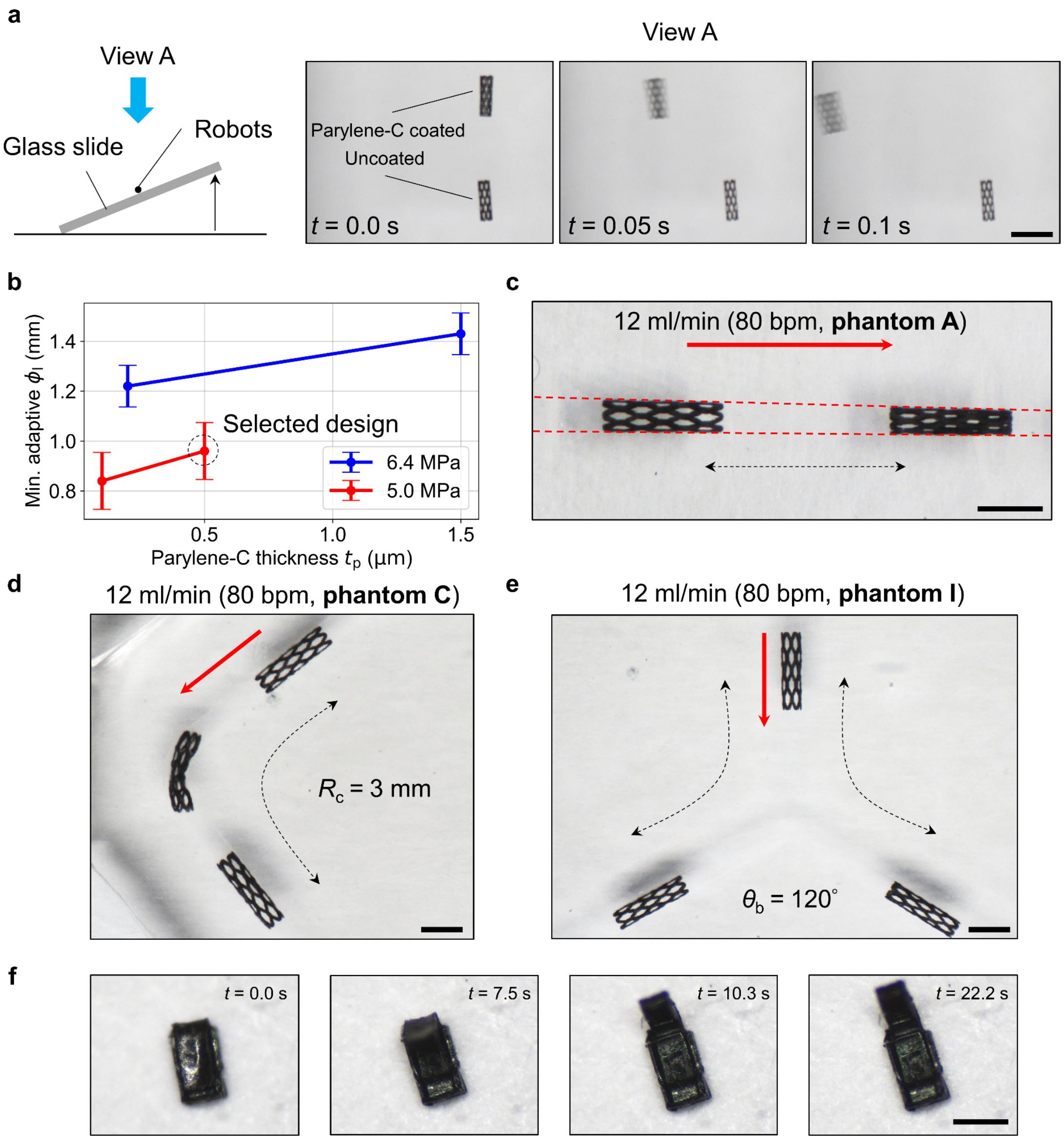

Supplementary Figure 26. Investigations on the effect of Parylene C coating. a Notable surface lubricity after coating on the robot. Robot body Young's modulus E_r = 5.0 MPa, coating thickness t_p = 0.5 μm. Scale bar: 5 mm. **b** Experimental optimization on the proper combination of E_r and t_p realizing the retrievably shape adaptation down to 1 mm lumen. The data are presented as mean values ± standard deviation for n = 5. **c – e** Maintenance of the locomotion capabilities (E_r = 5.0 MPa, t_p = 0.5μm). Scale bars: 3 mm. **f** Response maintenance of the SMP-based foldable structure

when the shape-memory transition temperature of 30 ± 5 °C is reached (t_p = 0.5 μm). Scale bar: 0.5 mm.

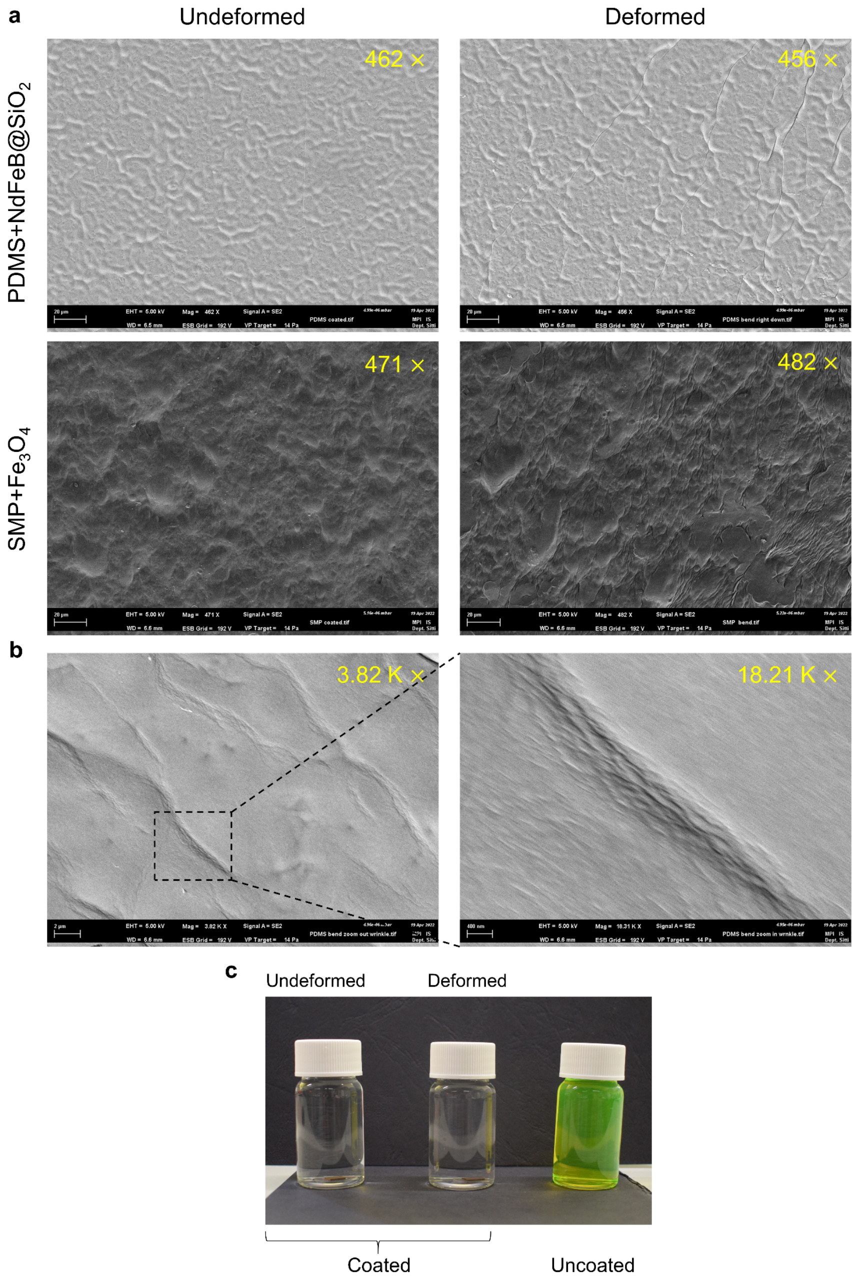

Supplementary Figure 27. Effect of deformation on the Parylene C coating layer. a SEM-based surface morphology inspection for Parylene C-coated PDMS + NdFeB@SiO_2 composite and Parylene C-coated SMP + Fe_3O_4 composite before and after deformation. **b** Zoom-in image of the wrinkles after deformation. **c** Leaching tests of Parylene C coated samples (deformed and undeformed) and uncoated samples in PBS (pH = 7.4) for 5 days. The PDMS + NdFeB composite

was compounded with fluorescein sodium. No visible fluorescence in the solution containing coating samples indicated the impermeability of Parylene C, and the deformation did not expose the substrates. In **a** and **b**, three surface inspections on different locations were conducted on each of three samples in each material group, and similar surface morphologies were observed for the deformed and undeformed samples, respectively. Sample size: 2 mm × 2 mm × 0.1 mm.

Supplementary Note 9. Experimental validation of force modeling

9.1 Magnetic forces

We utilized the widely accepted dipole model here to describe magnetic forces and torques[52] (equations 1 and 2). For experimental validation, first, we placed the handheld gaussmeter (HGM09s, MAGSYS magnet system GmbH, Germany) on the top of the permanent magnet and changed the mutual distance, l_{mag} (Supplementary Figure 28a), to measure the magnetic flux density along the global z-axis, B_z. These measurements agree well with the modeling results (Supplementary Figure 28b).

Then we directly measured the magnetic forces along with the robot attached z_r-axis, $F_{\text{mag,zr}}$, by customized non-magnetic acrylic board-made frame and the precision balance (LPC-313, Readability: 1 mg, VWR International LLC; Supplementary Figure 28c). The modeling results match well with the experimental data, with only a minor discrepancy at a small $l_{\text{mag}} = 40$ mm (Supplementary Figure 28d). This is caused by the discounted accuracy of the dipole approximation at a close location to the magnet center[53]. Given that we only focus on the analyses for $l_{\text{mag}} \geq 50$ mm, this mismatch has no notable negative effects on the modeling. A side note is that the height of the frame is 30 cm. When the robot does not appear, the movement of the permanent magnet within the investigation range of l_{mag}, i.e., 4 to 8 cm, did not lead to notable differences in the scale readings (~10^{-5} N). Given the validated magnetic field and the resultant magnetic forces, the dipole model could be validated.

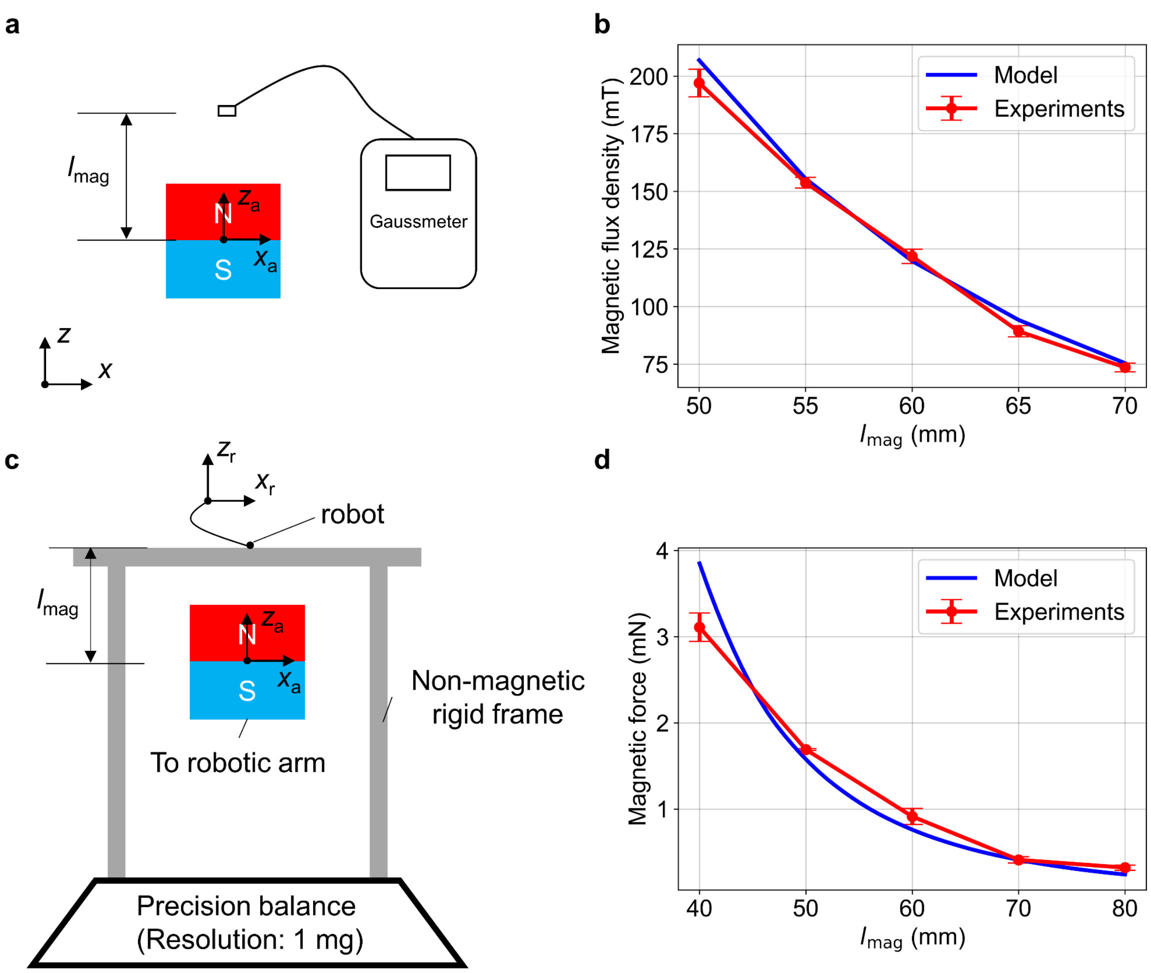

Supplementary Figure 28. Experimental validation for the modeling of magnetic field and magnetic forces. (**a** & **c**) Schematic of the setup to measure the magnetic flux density and magnetic forces, respectively. (**b** & **d**) Consistent modeled results and experimental measurements of magnetic flux density and magnetic force, respectively. The data are presented as mean values ± standard deviation for $n = 5$.

9.2 Radial forces

Besides the FEA model, we have also developed a simplified but effective analytical model based on the cantilever beam bending to understand the forces during radial deformation of the soft stent[54] (Supplementary Figure 29a). According to the force analyses on the simplified geometry, F_n can be expressed as

$$F_{\mathrm{n}} = 10\,\frac{3E_{\mathrm{r}}I_{\mathrm{b}}\Delta l_{\mathrm{c}}}{l_{\mathrm{b}}^{3}}\tan\left(\frac{\pi}{3}\right), \tag{19}$$

where E_r is Young's modulus of the robot material, I_b is the second moment of area for the cross-section of a single beam on the composing diamond-shaped cell, l_b is the length of the single beam, and Δl_c is the change in distance of the two facing beams of the cell under deformation.

To validate these two models, we measured the radial forces F_n by quantifying the robot's kinetic friction in various Φ_l. In phantom A, we connected the robot to the load cell of the mechanical tester's friction measurement setup (Instron 5942, Germany) by fine copper wire (Φ = 0.05 mm; Supplementary Figure 29b). Then, based on the widely accepted Coulomb model of friction and the quantified CoF as summarized in Supplementary Table 2, we acquired F_n as a function of Φ_l (Supplementary Figure 29c). These measurements agree with both FEA and analytical models with minor offset, which could be resulted from batch-to-batch differentiations in the modulus of the fabricated robot.

9.3 Fluidic drag

To validate the modeling of fluidic drag F_{drag}, we first experimentally recorded the flow rate that the maximum static friction $F_{fric,static}$ was overcome, and the robots started to move at each Φ_l, i.e., $F_{fric,static} = F_{drag}$. We computed these forces based on the quantified CoF and the F_n at the corresponding Φ_l. Meanwhile, these experimentally recorded flow rates were imported into the COMSOL simulation to compute F_{drag}, where we simulated the robot body as a smooth hollow cylinder for the convenience of reparametrization of the robot dimension (Supplementary Figure 29d). As shown in Supplementary Figure 29e, these results have shown good consistency. The slightly lower amplitude from the simulation could be explained by the less contribution from the friction drag of a meshed structure for the stent body.

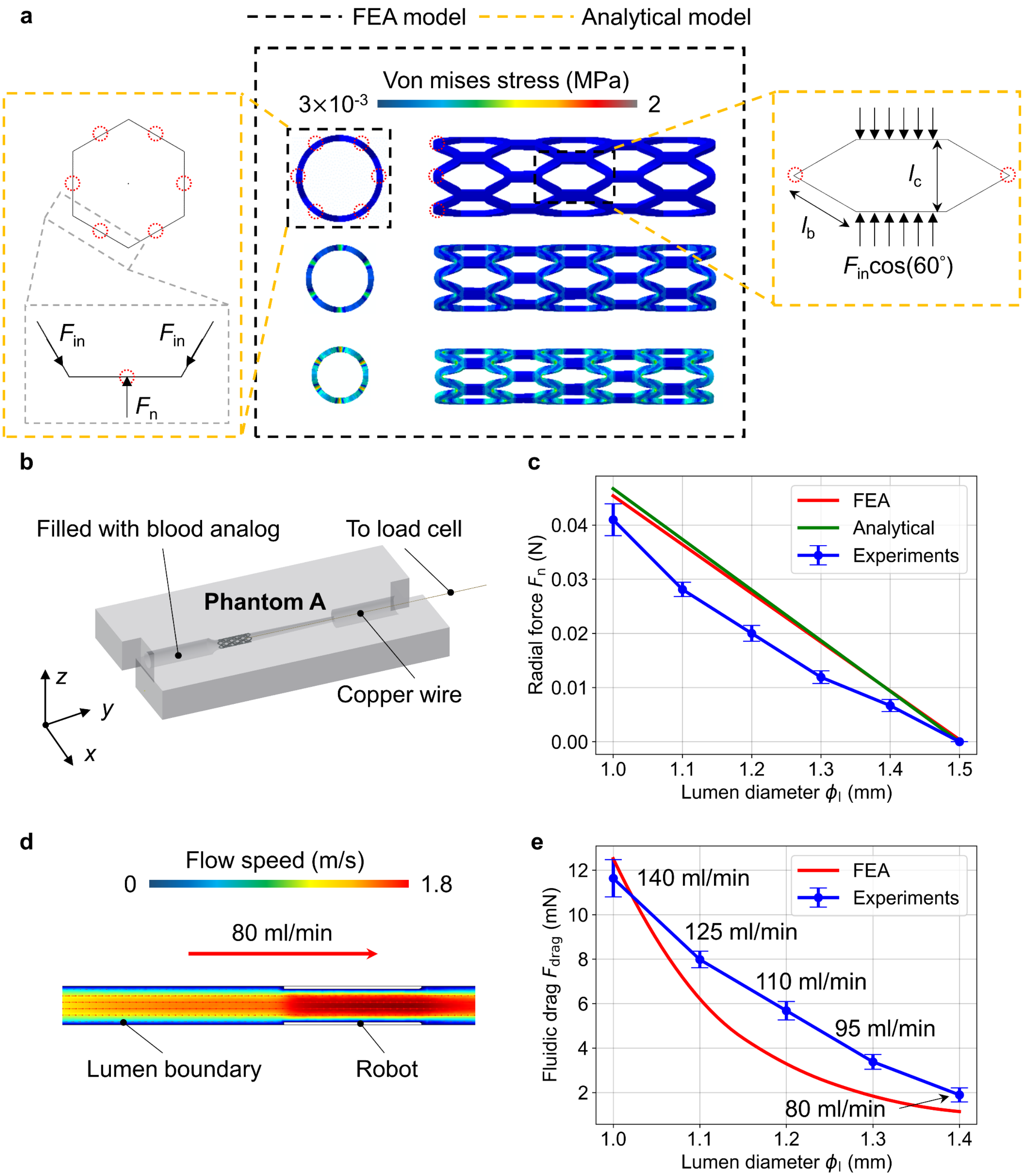

Supplementary Figure 29. Experimental validation for the modeling of radial forces and fluidic drag. a FEA and analytical modeling of the radial forces. The red dotted circles indicate the joints undergoing bending during the deformation. **b** Measurement of kinetic frictions as a function lumen diameter Φ_l to compute radial forces F_n. **c** Comparison of the modeling and experimental results for F_n. **d** A simulation example for the computation of fluidic drag F_{drag} (cross-

sectional view of the lumen). **e** Comparison of the modeling and experimental results for F_{drag}. The experimental-settled flow rate to acquire each F_{drag} is labeled aside. In **c** and **e**, The data are presented as mean values ± standard deviation for $n = 5$.

Supplementary References

1 Zhao, H. *et al.* Large vessel occlusion scales increase delivery to endovascular centers without excessive harm from misclassifications. *Stroke* **48**, 568-573 (2017).

2 Saver, J. L. *et al.* Thrombectomy for distal, medium vessel occlusions: a consensus statement on present knowledge and promising directions. *Stroke* **51**, 2872-2884 (2020).

3 Demchuk, A. M. *et al.* Recanalization and clinical outcome of occlusion sites at baseline CT angiography in the Interventional Management of Stroke III trial. *Radiology* **273**, 202-210 (2014).

4 Grossberg, J. A. *et al.* Beyond large vessel occlusion strokes: distal occlusion thrombectomy. *Stroke* **49**, 1662-1668 (2018).

5 Disorders, N. I. o. N. & Group, S. r.-P. S. S. Tissue plasminogen activator for acute ischemic stroke. *New Engl. J. Med.* **333**, 1581-1588 (1995).

6 Wu, C., Di Wu, J. C., Li, C. & Ji, X. Why not intravenous thrombolysis in patients with recurrent stroke within 3 months? *Aging Dis.* **9**, 309 (2018).

7 Ribo, M. *et al.* Difficult catheter access to the occluded vessel during endovascular treatment of acute ischemic stroke is associated with worse clinical outcome. *J. Neurointerv. Surg.* **5**, i70-i73 (2013).

8 Xavier, A. R. *et al.* Clinical potential of intra-arterial thrombolytic therapy in patients with acute ischaemic stroke. *CNS Drugs* **17**, 213-224 (2003).

9 Güneş, Y., Sincer, İ. & Erdal, E. Catheter-directed intra-arterial thrombolysis for lower extremity arterial occlusions. *Anatol. J. Cardiol.* **22**, 54 (2019).

10 Zhang, S. *et al.* Safety of intra-arterial tirofiban administration in ischemic stroke patients after unsuccessful mechanical thrombectomy. *J. Vasc. Interv. Radiol.* **30**, 141-147 (2019).

11 Ricci, A. *et al.* Cortical aneurysms of the middle cerebral artery: A review of the literature. *Surg. Neurol. Int.* **8**, 117 (2017).

12 Puri, A. S. *et al.* Safety, efficacy, and short-term follow-up of the use of Pipeline™ embolization device in small (< 2.5 mm) cerebral vessels for aneurysm treatment: single institution experience. *Neuroradiology* **58**, 267-275 (2016).

13 Lauzier, D. C. *et al.* Pipeline embolization of MCA aneurysms in the M2-M4 segment: Dual center study and meta-analysis. *Clin. Neurol. Neurosurg.* **212**, 107063 (2021).

14 Al-Schameri, A. R., Lunzer, M., Daller, C., Kral, M. & Killer, M. Middle cerebral artery aneurysm surgery after stent misplacement: A case report. *Interv. Neuroradiol.* **22**, 49-52 (2016).

15 Zhou, G., Su, M., Yin, Y.-L. & Li, M.-H. Complications associated with the use of flow-diverting devices for cerebral aneurysms: a systematic review and meta-analysis. *Neurosurg. Focus* **42**, E17 (2017).

16 Tsai, Y.-H., Wong, H.-F. & Hsu, S.-W. Endovascular management of spontaneous delayed migration of the flow-diverter stent. *J. Neuroradiol.* **47**, 38-45 (2020).

17 Takong, W. & Kobkitsuksakul, C. Delayed Proximal Flow Diverting Stent Migration in a Ruptured Intracranial Aneurysm: A Case Report. *Neurointervention* **15**, 154-157 (2020).

18 Shah, M. N. *et al.* The relationship of cortical folding and brain arteriovenous malformations. *Neurovasc. Imaging* **2**, 13 (2016).

19 Ajiboye, N., Chalouhi, N., Starke, R. M., Zanaty, M. & Bell, R. Cerebral arteriovenous malformations: evaluation and management. *Sci. World J.* **2014**, 649036 (2014).

20 Gross, B. A., Albuquerque, F. C., Moon, K. & McDougall, C. G. Evolution of treatment and a detailed analysis of occlusion, recurrence, and clinical outcomes in an endovascular library of 260 dural arteriovenous fistulas. *J. Neurosurg.* **126**, 1884-1893 (2017).
21 Maus, V. *et al.* Endovascular Treatment of Intracranial Dural Arteriovenous Fistulas: A German Single-Center Experience. *Cerebrovasc. Dis. Extra* **10**, 84-93 (2020).
22 Gould, J. Breaking down the epidemiology of brain cancer. *Nature* **561**, S40-S41 (2018).
23 Huang, R., Boltze, J. & Li, S. Strategies for improved intra-arterial treatments targeting brain tumors: a systematic review. *Front. Oncol.* **10**, 1443 (2020).
24 Joshi, S., Ellis, J. A., Ornstein, E. & Bruce, J. N. Intraarterial drug delivery for glioblastoma mutiforme. *J. Neurooncol.* **124**, 333-343 (2015).
25 Rechberger, J. S., Thiele, F. & Daniels, D. J. Status Quo and Trends of Intra-Arterial Therapy for Brain Tumors: A Bibliometric and Clinical Trials Analysis. *Pharmaceutics* **13**, 1885 (2021).
26 Zarrinkoob, L. *et al.* Blood flow distribution in cerebral arteries. *J. Cereb. Blood Flow Metab.* **35**, 648-654 (2015).
27 Uflacker, R. Atlas of vascular anatomy: an angiographic approach (2nd edition). (2007).
28 Lin, C., Kaper, H. J., Li, W., Splinter, R. & Sharma, P. K. Role of endothelial glycocalyx in sliding friction at the catheter-blood vessel interface. *Sci. Rep.* **10**, 11855 (2020).
29 Pancaldi, L. *et al.* Flow driven robotic navigation of microengineered endovascular probes. *Nat. Commun.* **11**, 6356 (2020).
30 Rafii-Tari, H. *et al.* Objective assessment of endovascular navigation skills with force sensing. *Ann. Biomed. Eng.* **45**, 1315-1327 (2017).
31 Gonzalez-Rodriguez, D. *et al.* Mechanical criterion for the rupture of a cell membrane under compression. *Biophys. J.* **111**, 2711-2721 (2016).
32 Peeters, E., Oomens, C., Bouten, C., Bader, D. & Baaijens, F. Mechanical and failure properties of single attached cells under compression. *J. Biomech.* **38**, 1685-1693 (2005).
33 Goyal, M., Kappelhof, M., Ospel, J. M. & Bala, F. Balloon guide catheters: use, reject, or randomize? *Neuroradiology* **63**, 1179-1183 (2021).
34 Vikner, T. *et al.* Characterizing pulsatility in distal cerebral arteries using 4D flow MRI. *J. Cereb. Blood Flow Metab.* **40**, 2429-2440 (2020).
35 Ball, R. *et al.* A comparison between Chinese and Caucasian head shapes. *Appl. Ergon.* **41**, 832-839 (2010).
36 Lu, H., Lam, L. C. & Ning, Y. Scalp-to-cortex distance of left primary motor cortex and its computational head model: Implications for personalized neuromodulation. *CNS Neurosci. Ther.* **25**, 1270-1276 (2019).
37 Takagi, Y. *et al.* Histopathological characteristics of distal middle cerebral artery in adult and pediatric patients with moyamoya disease. *Neurol. Med. Chir. (Tokyo)* **56**, 345–349 (2016).
38 Rajan, K. P., Al-Ghamdi, A., Parameswar, R. & Nando, G. Blends of thermoplastic polyurethane and polydimethylsiloxane rubber: assessment of biocompatibility and suture holding strength of membranes. *Int. J. Biomater.* **2013**, 240631 (2013).
39 Kim, S. *et al.* A biostable, anti-fouling zwitterionic polyurethane-urea based on PDMS for use in blood-contacting medical devices. *J. Mater. Chem. B* **8**, 8305-8314 (2020).
40 Kovach, K. M., Capadona, J. R., Gupta, A. S. & Potkay, J. A. The effects of PEG-based surface modification of PDMS microchannels on long-term hemocompatibility. *J. Biomed. Mater. Res. A* **102**, 4195-4205 (2014).

41 Xue, P. *et al.* Surface modification of poly (dimethylsiloxane) with polydopamine and hyaluronic acid to enhance hemocompatibility for potential applications in medical implants or devices. *ACS Appl. Mater. Interfaces* **9**, 33632-33644 (2017).
42 Donohue, V., McDonald, F. & Evans, R. In vitro cytotoxicity testing of neodymium-iron-boron magnets. *J. Appl. Biomater.* **6**, 69-74 (1995).
43 Wahajuddin & Arora, S. Superparamagnetic iron oxide nanoparticles: magnetic nanoplatforms as drug carriers. *Int. J. Nanomedicine* **7**, 3445-3471 (2012).
44 Kim, Y., Parada, G. A., Liu, S. & Zhao, X. Ferromagnetic soft continuum robots. *Sci. Robot.* **4**, eaax7329 (2019).
45 Brancato, L., Decrop, D., Lammertyn, J. & Puers, R. Surface nanostructuring of parylene-C coatings for blood contacting implants. *Materials* **11**, 1109 (2018).
46 Kuo, W.-C., Wu, T.-C., Wu, C.-F. & Wang, W.-C. Bioperformance analysis of parylene C coating for implanted nickel titanium alloy. *Mater. Today Commun.* **27**, 102306 (2021).
47 Sasaki, M. *et al.* Effect of Parylene C on the Corrosion Resistance of Bioresorbable Cardiovascular Stents Made of Magnesium Alloy 'Original ZM10'. *Materials* **15**, 3132 (2022).
48 Liu, Y. *et al.* Highly controllable and reliable ultra-thin Parylene deposition. *Micro Nanosyst. Lett.* **6**, 1-7 (2018).
49 Sun, M. *et al.* Reconfigurable Magnetic Slime Robot: Deformation, Adaptability, and Multifunction. *Adv. Funct. Mater.* **32**, 2112508 (2022).
50 Weber, M. *et al.* Blood-contacting biomaterials: in vitro evaluation of the hemocompatibility. *Front. Bioeng. Biotechnol.* **6**, 99 (2018).
51 Wright, D. *et al.* Reusable, reversibly sealable parylene membranes for cell and protein patterning. *J. Biomed. Mater. Res. A* **85**, 530-538 (2008).
52 Abbott, J. J., Diller, E. & Petruska, A. J. Magnetic Methods in Robotics. *Annu. Rev. Control Robot. Auton. Syst.* **3**, 57-90 (2020).
53 Petruska, A. J. & Abbott, J. J. Optimal Permanent-Magnet Geometries for Dipole Field Approximation. *IEEE Trans. Magn.* **49**, 811-819 (2013).
54 Hejazi, M., Sassani, F., Gagnon, J., Hsiang, Y. & Phani, A. S. Deformation mechanics of self-expanding venous stents: Modelling and experiments. *J. Biomech.* **120**, 110333 (2021).
