# Supplementary material for: Adaptive wireless millirobotic locomotion into distal vasculature
Source: Nat Commun. 2022 Aug 1;13:4465. doi: 10.1038/s41467-022-32059-9 (PMC9343456; doi:10.1038/s41467-022-32059-9)
Supplement: Supplementary file 2 — Description of Additional Supplementary Files [file 41467_2022_32059_MOESM2_ESM.pdf]

Description of Additional Supplementary Files

Supplementary Movie 1. Radial shape-adaptive locomotion with pulsatile flow
Supplementary Movie 2. Traversing among the curved route with pulsatile flow
Supplementary Movie 3. Traversing among bifurcations with pulsatile flow
Supplementary Movie 4. Locomotion in the tortuous route with pulsatile flow
Supplementary Movie 5. Locomotion among the sharp turn with pulsatile flow
Supplementary Movie 6. Locomotion among branches with pulsatile flow (2D)
Supplementary Movie 7. Locomotion among branches with pulsatile flow in the cranium simulant (3D)
Supplementary Movie 8. Adaptive locomotion in the porcine heart coronary artery with pulsatile flow
Supplementary Movie 9. Demonstration on cargo releasing
Supplementary Movie 10. Diversion of the pulsatile flow
