# Supplementary material for: Adaptive wireless millirobotic locomotion into distal vasculature
Source: Nat Commun. 2022 Aug 1;13:4465. doi: 10.1038/s41467-022-32059-9 (PMC9343456; doi:10.1038/s41467-022-32059-9)
Supplement: Supplementary file 13 — Reporting Summary [file 41467_2022_32059_MOESM13_ESM.pdf]

nature portfolio

Corresponding author(s): Dr. Wenqi Hu, Prof. Dr. Metin Sitti

Last updated by author(s): Jun 17, 2022

Reporting Summary

Nature Portfolio wishes to improve the reproducibility of the work that we publish. This form provides structure for consistency and transparency in reporting. For further information on Nature Portfolio policies, see our Editorial Policies and the Editorial Policy Checklist.

Statistics

For all statistical analyses, confirm that the following items are present in the figure legend, table legend, main text, or Methods section.

n/a	Confirmed	
☐	☒	The exact sample size (*n*) for each experimental group/condition, given as a discrete number and unit of measurement
☐	☒	A statement on whether measurements were taken from distinct samples or whether the same sample was measured repeatedly
☐	☒	The statistical test(s) used AND whether they are one- or two-sided<br>*Only common tests should be described solely by name; describe more complex techniques in the Methods section.*
☒	☐	A description of all covariates tested
☒	☐	A description of any assumptions or corrections, such as tests of normality and adjustment for multiple comparisons
☐	☒	A full description of the statistical parameters including central tendency (e.g. means) or other basic estimates (e.g. regression coefficient) AND variation (e.g. standard deviation) or associated estimates of uncertainty (e.g. confidence intervals)
☐	☒	For null hypothesis testing, the test statistic (e.g. *F*, *t*, *r*) with confidence intervals, effect sizes, degrees of freedom and *P* value noted<br>*Give P values as exact values whenever suitable.*
☒	☐	For Bayesian analysis, information on the choice of priors and Markov chain Monte Carlo settings
☒	☐	For hierarchical and complex designs, identification of the appropriate level for tests and full reporting of outcomes
☒	☐	Estimates of effect sizes (e.g. Cohen's *d*, Pearson's *r*), indicating how they were calculated

Our web collection on statistics for biologists contains articles on many of the points above.

Software and code

Policy information about availability of computer code

Data collection	Simulations of three-dimensional fluid-structure interaction between the robot and flow were performed using COMSOL Multiphysics 5.4.<br>Solid mechanics simulations of the robot deformation were performed using Abaqus 2019.<br>Camera Blackfly S USB3 was used to collect visual data using SpinView 2.4.0.<br>The actuation and manipulation of the permanent magnet by the robotic arm and step motor was using the code packages compatible with the ROS environment written in Python 2.7.17.<br>Medical ultrasound imaging was collected using a public ROS driver for video stream.<br>X-ray imaging was collected using the commercial software KubtecNC 3.0.0.0.
Data analysis	MATLAB R2018a<br>Python 3.8<br>Image J 1.53

For manuscripts utilizing custom algorithms or software that are central to the research but not yet described in published literature, software must be made available to editors and reviewers. We strongly encourage code deposition in a community repository (e.g. GitHub). See the Nature Portfolio guidelines for submitting code & software for further information.

Data

Policy information about availability of data

All manuscripts must include a data availability statement. This statement should provide the following information, where applicable:

- Accession codes, unique identifiers, or web links for publicly available datasets
- A description of any restrictions on data availability
- For clinical datasets or third party data, please ensure that the statement adheres to our policy

The data evaluating the findings in this study are included in the article and its supplementary information. All data are available from the corresponding author upon reasonable request.

Human research participants

Policy information about studies involving human research participants and Sex and Gender in Research.

Reporting on sex and gender	No human research participants were involved.
Population characteristics	Not relevant.
Recruitment	Not relevant.
Ethics oversight	Not relevant.

Note that full information on the approval of the study protocol must also be provided in the manuscript.

Field-specific reporting

Please select the one below that is the best fit for your research. If you are not sure, read the appropriate sections before making your selection.

[x] Life sciences [ ] Behavioural & social sciences [ ] Ecological, evolutionary & environmental sciences

For a reference copy of the document with all sections, see nature.com/documents/nr-reporting-summary-flat.pdf

Life sciences study design

All studies must disclose on these points even when the disclosure is negative.

Sample size	For the quantitative analyses in the physiologically relevant PDMS-based phantoms fabricated using the unified methods as stated in the paper, at least 2 robot samples (less than 5) were used for at least 3 measurements in the investigation of each variable’s effect to include the variability in robot fabrications. For example, to investigate the effects of magnet translation speed on robot locomotion speed (Fig. 2e), 2 robots were tested 5 times at each magnet translation speed.<br>For the demonstrations in the coronary arteries, 4 robots were tested in 4 fresh porcine coronary arteries ex vivo within 48 hrs after the animals were slaughtered and the hearts were collected from the slaughterhouse. The sample size was determined to include the variability in robot fabrications and the organ tissues.<br>For the biocompatibility and hemocompatibility studies, in each study for each material type, 3 Parylene C-coated samples were used to include the variability in the quality of the coating.
Data exclusions	No data were excluded from the analyses.
Replication	All the experimental findings were replicated with the corresponding sample sizes as indicated in the caption of the relevant figure. All these findings were replicated successfully at different times.
Randomization	For the quantitative analyses based on PDMS-based phantoms, random robot samples were selected in each investigation.<br>For the demonstrations in the coronary arteries, we randomly picked the coronary arteries and robots to demonstrate the locomotion capabilities of the robots.<br>For the biocompatibility and hemocompatibility studies, we have Parylene C-coated more than 10 material samples for each of the 2 material types and randomly selected 3 samples used for each specific study.
Blinding	For the quantitative analyses in PDMS-based phantoms, the specific design parameters of the robots were known before the experiments to investigate their effects on robot locomotion and functions. Therefore, blinding was not relevant.<br>For the tests in porcine coronary arteries, blinding was not relevant since they were used for demonstrations in the current study.<br>For the biocompatibility and hemocompatibility studies, we confirmed the successful coating of Parylene C coating and evaluated its effects on the 2 material types. Therefore, blinding was not relevant.

Reporting for specific materials, systems and methods

We require information from authors about some types of materials, experimental systems and methods used in many studies. Here, indicate whether each material, system or method listed is relevant to your study. If you are not sure if a list item applies to your research, read the appropriate section before selecting a response.

Materials & experimental systems

n/a	Involved in the study	
x		Antibodies
	x	Eukaryotic cell lines
x		Palaeontology and archaeology
x		Animals and other organisms
x		Clinical data
x		Dual use research of concern

Methods

n/a	Involved in the study	
x		ChIP-seq
x		Flow cytometry
x		MRI-based neuroimaging

Eukaryotic cell lines

Policy information about cell lines and Sex and Gender in Research

Cell line source(s)	The murine monocyte-macrophage cell line J774A.1 (ATCC) was used as the model cell line for cell viability studies.<br>The cell line purchased was ATCC catalog # TIB-67.
Authentication	The authentication procedures were three-fold. The first determination was the morphological characteristics of murine macrophages, detected with microscopy. The second determination was cell surface marker characterization, and this was collected with flow cytometry and surface markers CD11b, CD14, CD80, and CD16. The third determination was the capability to undergo phagocytosis of particles or materials. This was determined by timelapse video of seeded cells incubated with small particles, which were uptaken by cells over 24 hour period.
Mycoplasma contamination	It was confirmed as negative for Mycoplasma contamination, as determined by (MycoFluor Detection Kit) (Invitrogen) (Cat# M7006).
Commonly misidentified lines<br>(See ICLAC register)	According to ICLAC this is not a commonly misidentified cell line.
